# Clock-related neuropeptides in *Acyrthosiphon pisum*

**DOI:** 10.3389/fgene.2026.1818975

**Published:** 2026-06-12

**Authors:** Francesca Sara Colizzi, Sander Liessem, Jose Ricardo Morales-Poole, David Martínez-Torres, Charlotte Helfrich-Förster

**Affiliations:** 1 Neurobiology and Genetics, Theodor-Boveri-Institute, Biozentrum, Julius-Maximilians-University of Würzburg, Würzburg, Germany; 2 Institut de Biologia Integrativa de Sistemes (I2SysBio), Universitat de València, Paterna, Spain

**Keywords:** aphid, circadian clock, immunohistochemistry, insect, mass spectrometry, neuropeptides, photoperiodism, transcriptomics

## Abstract

**Introduction:**

The pea aphid (*Acyrthosiphon pisum*) is a strongly photoperiodic insect. Although the involvement of the circadian clock in insect photoperiodism is well established, the neuronal and molecular mechanisms by which clock neurons convey photoperiodic information remain largely unknown. Neuropeptides commonly act as neuromessengers in circadian clock neurons, yet little is known about the neuropeptidergic organization of the circadian clock in the pea aphid.

**Methods:**

Here, we characterized the complete set of neuropeptides in the aphid brain and, using a combination of transcriptomic and peptidomic approaches, and investigated the neuropeptides expressed in the circadian clock neurons using immunohistochemistry.

**Results:**

We confirmed a large number of peptides, including several peptides described for the first time in this species. Furthermore, we found that circadian clock neurons express a variety of these neuropeptides, some of which overlapped within the same clock neuron type. In the lateral clock neurons, we found Pigment-Dispersing Factor, FMRFamide, Orcokinin a and Allatotropin, a neuropeptide profile that closely resembles that of cockroach clock neurons but differs markedly from that of the fruit fly, with the exception of Pigment-Dispersing Factor. In dorsal clock neurons, which are homologous to those that are presynaptic to insulin-producing cells in *Drosophila*, we detected Allatostatin A, Diuretic Hormone 31, FMRFamide, and Myoinhibitory peptide.

**Discussion:**

Together, our findings provide the first comprehensive map of neuropeptides associated with the pea aphid circadian clock, offering new insights into how clock neurons may regulate seasonal responses and establishing a foundation for future functional studies of neuropeptide-mediated photoperiodism

## 1 Introduction

The pea aphid *Acyrthosiphon pisum* is a widely distributed agricultural pest insect in temperate regions, responsible for large-scale economic losses primarily through the transmission of plant viral diseases (Paudel et al., 2018; Rashed et al., 2018). During spring and summer, aphids reproduce asexually through parthenogenesis at high rates and with short generation times. Their reproductive cycle is photoperiod-dependent meaning that in response to shortening of the photoperiod, aphids undergo a drastic shift from asexual to sexual reproduction (Saunders, 2013; Takeda and Suzuki, 2022; Hardie, 2009). This photoperiodic switch is an adaptive strategy to survive winter. Sexual morphs mate, and females lay cold-resistant eggs that enter diapause and hatch in spring, giving rise to new parthenogenetic generations, while adults may die due to harsh winter conditions. Deciphering the molecular mechanisms underlying this photoperiodic response provides insights into seasonal regulation and developmental plasticity. It also offers potential targets for pest control strategies aimed at reducing pesticide resistance and limiting their environmental impact, and may provide a basis for the design of neuropeptide mimetics targeting photoperiodic regulatory pathways to alter aphid physiology.

Circadian clocks are endogenous systems that allow organisms to anticipate daily environmental cycles, including changes in light, temperature, and food availability (Hurd and Ralph, 1998; Ouyang et al., 1998; Pittendrigh and Minis, 1972). Apart from their role in daily rhythms, they are widely recognized as key players in insect photoperiodism (Bünning, 1936; Saunders, 2021), the ability of organisms to adjust their physiology and behavior in response to day length (photoperiod). In aphids, however, the role of the circadian clock in controlling the reproductive mode switch remains debated, and the underlying neuroanatomical and molecular pathways are still largely unresolved (Colizzi et al., 2024).

In animals, the central circadian clock is located in the brain, is entrained by the 24-h light–dark cycle, and regulates physiological rhythms through neuronal, hormonal, and peptidergic pathways (King and Seghal, 2018; Pilorz et al., 2018). Studies across diverse insect species revealed a general conservation in the anatomical position of clock neurons, their functional roles, and the brain areas they innervate (Numata et al., 2015; Beer and Helfrich-Förster, 2020; Colizzi et al., 2021). Here, the circadian clock has been most extensively characterized in *Drosophila melanogaster*, largely due to the availability of powerful genetic tools. In *Drosophila*, clock neurons are organized into distinct clusters, named according to their soma location: dorsal neurons (DNs) and lateral neurons (LNs). These main groups are further subdivided into several subgroups according to their neuropeptide expression (Reinhard et al., 2024).

Each clock neuron expresses a set of core clock genes, *period* (*per*), *timeless* (*tim*), *Clock* (*Clk*), and *cycle* (*cyc*), whose protein products (PER, TIM, CLK, and CYC) interact in transcriptional–translational feedback loops, resulting in the rhythmic expression of downstream clock-output genes (Helfrich-Förster, 2017; Takahashi, 2021). The clock is reset daily via light input from the compound eyes and the blue-light photoreceptor CRYPTOCHROME (CRY) (Helfrich-Förster, 2020). The molecular components of the core clock (PER, TIM and CRY) have also been elucidated in pea aphids (Barberà et al., 2017; Colizzi et al., 2021; Colizzi et al., 2023) but the neuropeptides associated with the circadian clock network remain still largely unknown.

Circadian clock neurons are generally rich of neuropeptides that play a central role in modulating the activity of different neuronal clusters and shaping the overall output of the clock network (Homberg et al., 2003; Lin, 2004; Taghert and Nitabach, 2012; Mezan et al., 2016; Reinhard et al., 2024; Neupert et al., 2025). Among them, Pigment-dispersing Factor (PDF) is the most extensively studied clock-related neuropeptide in insects, serving as a key communication factor within the network (Lin, 2004; Wei et al., 2014). PDF controls behavioral rhythmicity (Reischig and Stengl, 2003; Shafer and Yao, 2014), memory formation (Inami et al., 2021), metabolism (Braco et al., 2022), and seasonal timing (Kotwica-Rolinska et al., 2022; Hasebe et al., 2022) in part by modulating activity of the Insulin-Producing cells (IPCs) that express Insulin-like peptides (ILPs) (Hidalgo et al., 2023; Nagy et al., 2019). In all insects studied so far, PDF is expressed in clock neurons associated with the accessory medulla (AME), a small neuropil at the anterior ventromedial edge of the medulla which is regarded as the location of the circadian pacemaker (Helfrich-Förster, 2009; Shafer and Yao, 2014; Stengl and Arendt, 2016). Other neuropeptides associated with the AME of *Drosophila* are Ion Transport Peptide (ITP), short neuropeptide F (sNPF), neuropeptide F (NPF) and CCHamide1 (Johard et al., 2008; Johard et al., 2009; Fujiwara et al., 2018). In the cockroach, another important insect model for circadian clocks and related peptides, the AME and surrounding cells express other neuropeptides besides PDF, including Allatotropin (AT), Allatostatin A (AstA), FMRFamide (FMRFa), Leucokinin (LK), Orcokinin_a_ (OK_a_), and Myoinhibitory Peptide (MIP, also known as AstB). Notably, AT, OK_a_, PDF, and MIP can induce behavioral phase shifts similar to those triggered by light, suggesting that they may provide parallel pathways for light input to the clock (Stengl et al., 2015; Petri et al., 1995; Petri et al., 2002).

**FIGURE 1:**
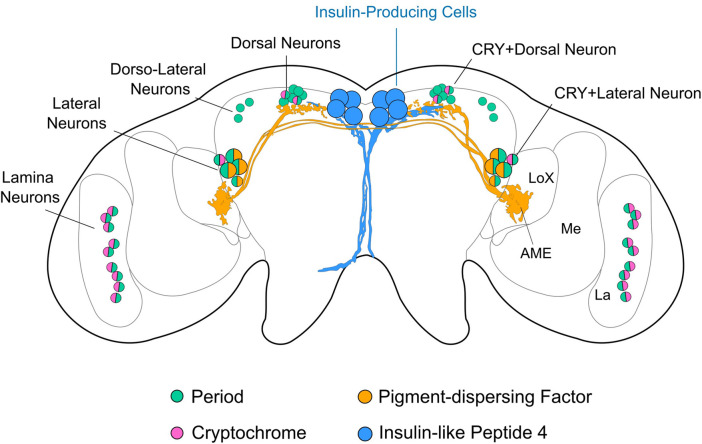
Schematic depiction of the pea aphid clock neurons clusters, some of which express PDF and are set up to contact IPCs expressing ILP4. The pea aphid possesses four clock neurons clusters: the dorsal neurons, the dorso-lateral neurons, the lateral neurons and the lamina neurons. Only the four CRY-negative lateral neurons express the neuropeptide PDF (orange), arborize in the accessory medulla (AME) and project toward the dorsal neurons. The dorsal projections are in close vicinity to the dendrites of Insulin-like Peptide 4-positive insulin-producing cells, ending in punctate immunostaining that suggests that Pigment-dispersing Factor is likely released at this site. La, lamina; Me, medulla; LoX: lobula complex.

In our previous studies, we described the neuroanatomy of the circadian clock of the pea aphid, highlighting the clock clusters containing PERIOD and CRYPTOCHROME and the PDF-immunoreactive clock neurons, whose projections extend toward the aphid IPCs (Colizzi et al., 2021; Colizzi et al., 2023) (Figure 1).

However, the full set of neuropeptides involved in the aphid circadian clock remains unknown. About 40 neuropeptides have been previously described in *A. pisum* using biochemical and genetic tools (Huybrechts et al., 2010), but none of the predicted neuropeptide products have been confirmed by fragmentation analysis. In addition, several novel neuropeptides have been described in other species since then (Kotwica-Rolinska et al., 2020; Boerjan et al., 2010; Hauser et al., 2010; Marciniak et al., 2020; Hummon et al., 2006; Liessem et al., 2018; Jiang et al., 2013; Roller et al., 2016).

In this report we aim at establishing a comprehensive list of the pea aphid brain neuropeptide repertoire. As a first step, we expanded the existing neuropeptide precursor list using publicly available transcriptomes and identified several neuropeptide precursors not previously reported in aphids. Using MALDI-TOF and Orbitrap mass spectrometry analysis, we detected 81 peptide products from 23 different neuropeptide precursors in the aphid central nervous system. 68 of these peptides from 19 neuropeptide precursors could be confirmed via subsequent fragmentation analysis. Among these, 49 mature peptides were confirmed in both the central brain (CB) and optic lobes (OL), while only 29 mature peptides were expressed in the OL.

By performing multiple-labelling immunohistochemistry using antisera directed against neuropeptides confirmed via mass spectrometry, as well as against clock proteins, we show that besides PDF, seven different neuropeptides (AstA, DH31, MIP, OK_a_, FMRFa, CCHamide1 and AT) are present in aphid clock neurons, while others such as ITP, NPF, and AstC, are not. This demonstrates that the neuropeptide composition in the aphid circadian clock neurons resembles more that of cockroaches than that of fruit flies. This study provides new insights into the unique and conserved features of the aphid circadian clock. Adding details to its neuroanatomy, it contributes to a better understanding of the regions potentially involved in receiving light input for daily synchronization, the pathways through which the clock might regulate behavior, and the connections between the clock and the neurosecretory cells, that in turn might modulate hormonal status in a season-dependent manner.

## 2 Materials and methods

### 2.1 Aphid strain and rearing

In this study, we used the LSR1 pea aphid line, originally collected in Ithaca, New York, United States (Caillaud et al., 2002). Aphids were grown on fava beans (*Vicia faba*) in climate chambers (Sanyo/Panasonic MLR-352H series; 18 °C ± 0.5 °C, 80% ± 10% relative humidity) at a 24 h light-dark cycle of 16 h of light and 8 h of darkness (16L:8D).

### 2.2 *De novo* assembly of nucleotide sequences

*De novo* assembly of RNA-Seq data was conducted by using Trinity (v2.2.0) (Grabherr et al., 2011). Assemblies were made from third party data submissions and publicly available SRA of *A. pisum* (SRR063706, PRJNA79725; SRR7037537, PRJNA450900; SRR9209839, PRJNA547535; SRR9209842, PRJNA547535; SRR12776646, PRJNA667506; SRR7037541, PRJNA450900) that include raw sequence reads made from whole body, head, salivary glands, and gut tissues. Assemblies were scanned and filtered using the NCBI Foreign Contamination Screen FCS-GX and uploaded to NCBI (TSAs: SUB15933239, DBNERU000000000; SUB15971964, DBMFTB000000000; SUB15971979, DBMFTC000000000; SUB15972000, DBNERV000000000; SUB15972023, DBMFTD000000000; SUB15972056, DBNERW000000000. The BioProject ID is SUB15956899). All transcriptome assemblies were evaluated using BUSCO v6.0.0 with the eukaryota_odb10 lineage dataset in transcriptome mode. Complete BUSCO scores are listed in Supplementary File S5 and in general had high-quality scores across samples.

### 2.3 Compiling of neuropeptide precursor sequences

Database searches for *A. pisum* neuropeptide and protein precursor sequences were primarily conducted using the tblastn algorithm from the BLAST + suite command line tool (v2.4.0.) (Camacho et al., 2009). Neuropeptide precursor sequences of *A. pisum* (Huybrechts et al., 2010) and other insects were used as reference queries (Habenstein et al., 2021; Liessem et al., 2018; Ragionieri et al., 2022). Aligned and assigned nucleotide sequences were translated into protein sequences using the ExPASy translate tool (http://web.expasy.org/translate/; Swiss Institute of Bioinformatics, Switzerland) (Gasteiger et al., 2003). Precursor sequence signal peptides (SP) were predicted using SignalP 6.0 (Teufel et al., 2022). If no SP could be predicted or no stop codon was present, precursors were considered incomplete.

### 2.4 Mass spectrometry

Animals were cooled at 4 °C for 10 min and transferred into a well containing chilled (approximately 6 °C) physiological phosphate-buffered saline (PBS, Sigma-Aldrich). Brains and individual optic lobes were dissected and adjacent tissues such as fat body, trachea and also pigmented layer of optic lobes were removed. Brain and optic lobes were analyzed separately in order to reduce tissue-specific peptide diversity and facilitate the identification of candidate neuropeptides for subsequent cellular localization studies (see below). For MALDI-TOF MS analysis, samples were then transferred onto a sample plate into a drop of distilled water (∼100 μL), which was removed immediately after the transfer. Samples were covered 0.1–0.2 μL α-cyano-4-hydroxycinnamic acid (CHCA, Sigma-Aldrich). A stock solution of the matrix was prepared with 10 mg/mL CHCA dissolved in 60% ethanol, 36% acetonitrile, 4% HPLC grade water, and 0.5% formic acid (FA). Prior to analysis, CHCA stock solution was diluted 1:3 with 50% methanol/water. Mass fingerprint spectra (MS^1^) were acquired in positive ion mode with an ultrafleXtreme TOF/TOF mass spectrometer (Bruker Daltonics, Fremont, CA, United States). MS acquisitions were made under manual control in reflector positive mode in a detection range of m/z 600–4,000. Instrument settings were optimized for this mass range and calibrated using Peptide Calibration Standard II (8222570, Bruker Daltonics) standard kits (mass accuracy 1.4 ppm). Data were processed with flexAnalysis 3.4 (Bruker Daltonics). Tandem mass spectrometry (MS^2^) experiments were performed with LIFT™ technology without CID and peptide identities were verified by comparison to theoretical (http://prospector.ucsf.edu) and experimentally obtained fragments.

For quadrupole Orbitrap MS with nanoflow HPLC, samples were processed as described elsewhere (Habenstein et al., 2021; Liessem et al., 2018; Predel et al., 2018). In brief, samples were transferred into a 20 μL peptide extraction buffer (50% MeOH in 1% trifluoroacetic acid (TFA)) after dissection. Samples were sonicated for 5 min in an ultrasonic bath, centrifuged for 2 min at 4 °C (15,000 rpm), again treated with multiple, contiguous bursts using an ultrasonic homogenizer (Bandelin Sonopuls HD 200, BANDELIN electronic GmbH & Co. KG, Berlin, Germany), replenished with 20 μL water and finally centrifuged at 15,000 rpm and 4 °C for 15 min. The supernatants were transferred into safe-lock tubes and evaporation of the organic solvent was accelerated using a low speed centrifuge under low pressure vacuum (Hetovac VR-1, Heto Lab Equipment) until the volume of the supernatants was reduced to about 15 μL. Samples were desalted using self-packed Stage Tip C18 (IVA Analysentechnik e. K., Meerbusch, Germany) spin columns9. Peptides were then separated on an EASY nanoLC1000 UPLC system (Thermo Fisher Scientific) using in-house packed RPC18-columns 50 cm (fused Silica tube with ID 50 μm ± 3 μm, OD 150 μm ± 6 μm; Reprosil 1.9 μm, pore diameter 60A°; Dr. Maisch GmbH, Ammerbuch-Entringen, Germany) and a binary buffer system (A: 0.1% FA; B: 80% ACN, 0.1% FA). Running conditions were as follows: linear gradient from 2 to 62% B in 110 min, 62%–75% B in 30 min, and final washing from 75% to 95% B in 6 min (45 °C, flow rate 250 nL/min). Finally, the gradients were re-equilibrated for 4 min at 5% B. The HPLC was coupled to a Q-Exactive Plus (Thermo Scientific, Bremen, Germany) mass spectrometer. MS data were acquired in a top-10 data-dependent method dynamically choosing the most abundant peptide ions from the respective survey scans in a mass range of 300–3,000 m/z for HCD fragmentation. Full MS^1^ acquisitions ran with 70,000 resolution, with automatic gain control target (AGC target) at 3e6 and maximum injection time at 80 ms. HCD spectra were measured with a resolution of 35,000, AGC target at 3e6, maximum injection time at 240 ms, 28 eV normalized collision energy, and dynamic exclusion set at 25 s. The instrument was run with peptide recognition mode (i.e., from two to eight charges); singly charged and unassigned precursor ions were excluded. Raw data were analyzed with PEAKS 10 (PEAKS Studio 10.6, BSI, ON, Canada).

### 2.5 Antibody generation against aphid CCHa1

A polyclonal antiserum against a custom synthetic peptide (CQVLDPRIEYKIMKI) was raised in rabbit by Moravian-Biotechnology Ltd. (Brno; Czech Republic). The custom peptide, also obtained from Moravian-Biotechnology Ltd., corresponds to the 14 C-terminal amino acids of the *A. pisum* CCHa1 precursor, with a cysteine added at the N-terminal for BSA coupling. After rabbit immunisation and animal bleeding, antiserum was obtained by affinity purification column using the synthetic peptide used as immunogen.

### 2.6 Immunohistochemistry

Antibody staining was performed as described in Colizzi et al. (2021). In brief, aphids were fixed in 4% paraformaldehyde (PFA) in 1X phosphate-buffered saline (PBS, Sigma-Aldrich) containing 0.5% Triton-X100 (PBST) for 4 h in the dark at room temperature (RT). After fixation, aphids were washed 3 × 10 min in phosphate-buffered saline (PBS), and the brains were dissected in PBS and washed 6 × 10 min in PBST. Brains were preincubated in blocking solution containing 5% normal goat serum (NGS, in PBST) for 2 h at RT or overnight at 4 °C. Brains were then incubated in the respective primary antisera (Table 1, all dissolved in NGS containing NaN_3_) for 48 h at 4 °C. Next, brains were washed for 6 × 10 min in PBST and incubated in the secondary antibody solution (5% NGS, secondary antibodies 1:200, in PBST) for 4 h in the dark at RT. The secondary antibodies used were the following: Alexa Fluor 488 or 633 goat anti-guinea pig 1:200; Alexa Fluor 488 or Alexa Fluor 647 goat anti-rat 1:200; Alexa Fluor 555 or 633 goat anti-rabbit 1:200 (Thermo Fisher Scientific, Waltham, MA, United States). Finally, brains were washed 4 × 10 min RT in PBST, rinsed two times in PBS, and embedded in Vectashield Antifade mounting medium (H-1000, Vector Laboratories, CA, United States). Since only very few antibodies specific to aphid peptides are currently available (see below), all other antibodies used in this report were developed for other insect species, which have been shown to recognize the corresponding peptides across multiple insect species. To validate our approach, we relied on transcriptomic and mass spectrometry data to confirm the presence of the target neuropeptides prior to performing immunostainings. Furthermore, being aware that peptide sequences can vary across species, we verified that the epitope recognized by each antibody was either identical or highly similar to the corresponding sequence in aphids. All used primary antibodies (Table 1) were well characterized. Several of them were specifically raised against the relevant protein or peptide of *A. pisum* (e.g., anti-PER, anti-PDF, anti-ILP4 (Colizzi et al., 2021; Cuti et al., 2021) and anti CCha1 generated in the present study) others were undertaken rigorous specificity tests (e.g., anti-CRY1; Colizzi et al., 2021), and for still others we performed pre-absorption tests on synthetic *A. pisum* peptides (Shanghai NovoPro Biotechnology Co., Ltd., Shanghai, China). For these pre-absorption controls, the primary antibodies were incubated for 2 days at 4 °C with the corresponding synthetic peptides at concentrations between 10^−3^ M and 10^−7^ M. The preabsorbed antibody solutions were then applied in place of the regular primary antibody in the staining procedure described above. Furthermore, we performed BlastP searches (NCBI), in which we compared the peptide sequences against which the antibodies were generated with the annotated protein database of *A. pisum* to determine whether the peptide sequences in question are also found in other proteins, enzymes, or peptides. We found that pre-absorption resulted in a significant reduction in immunofluorescence, and/or the BlastP search did not yield any further plausible matches in the annotated protein database for *A. pisum*. In addition, for some peptides, we used independent antibodies raised in different host species to further confirm the specificity of the labeling.

**TABLE 1:** Primary antibodies used in this study.

Antibody	Immunogen	Dilution	Host	References	RRID
*Anti*-period (PER)	*A. pisum* PER, residues 969–1,018	1:3,000	Rat	Colizzi et al. (2021)	AB_2920831
*Anti*-Cryptochrome1 (CRY1)	*Drosophila* CRY, polyhistidine-tagged full-length protein	1:1,000	Rabbit	Yoshii et al. (2008)	AB_2314242
*Anti-*pigment dispersing factor (PDF)	Synthetic *A. pisum* PDF-associated peptide (CSLYVPDDNFVIEEQNAPIAT)	1: 5,000	Guinea pig	Colizzi et al. (2023)	​
*Anti*-insulin-like- peptide 4 (ILP4)	Peptide-C of *A. pisum* ILP4 (SEIDFDVWDYKDLEDYNAVDYP), conjugated to BSA	1:8,000	Guinea pig	Cuti et al. (2021)	​
*Anti*-allatostatin A (AstA)	*Diploptera punctata* AstA (APSGAQRLYGFGLamide), conjugated to bovine thyroglobulin	1:300	Rabbit	Vitzthum et al. (1996)	AB_2313972
*Anti*-allatostatin A (AstA)	*Diploptera punctata* AstA (APSGAQRLYGFGLamide), conjugated to bovine serum albumin	1:50	Mouse	Stay et al. (1992)	AB_528076
*Anti-*CCHamide1 (CCHa1)	*Drosophila* CCHa1 (SCLEYGHSCWGAHGKR)	1:1,000	Rabbit	Veenstra and Ida (2014)	​
*Anti-*CCHamide1 (CCHa1)	*A. pisum* CCHa1 (C-QVLDPRIEYKIMKI), conjugated to bovine serum albumin	1:200	Rabbit	This paper	​
*Anti*-diuretic hormone 31 (DH31)	*Drosophila* DH31, full-length peptide	1:1,000	Rabbit	Park et al. (2008)	AB_2569126
*Anti*-orcokinin_a_ (OK_a_)	*Orconectes limosus* Asn13-ORC (NFDEIDRSGFGFN)	1:5,000	Rabbit	Bungart et al. (1994)	AB_2315017
*Anti*-SIFamide (SIFa)	*Drosophila* SIFa (AYRKPPFNGSIFamide)	1:1,000	Rabbit	Terhzaz et al. (2007)	AB_2315327
*Anti*-FMRFamide (FMRFa)	FMRFamide, conjugated to succinylated thyroglobulin	1:2000	Rabbit	Marder et al. (1987)	AB_2314414
*Anti*-allatotropin (AT)	*Manduca sexta* AT (GFKNVEMMTARGFamide), conjugated to thyroglobulin	1:2,500	Rabbit	Veenstra and Hagedorn (1993)	AB_2313973
*Anti*- myoinhibiting peptide (MIP)	*Periplaneta americana* MIP-1 (GWQDLQGGWamide) conjugated to thyroglobulin	1:8,000	Rabbit	Predel et al. (2001)	AB_2314803

### 2.7 Imaging

Immunofluorescent labelling was visualized with a Leica TCS SPE confocal microscope (Leica, Wetzlar, Germany) via Leica Application Suite X (Leica Microsystems) using 20X and 40X objectives (ACS APO 20X/0.6 and 40X/1.15 OIL). Fluorophore signals were detected in serial stacks (1.5–2 μm) and a minimum resolution of 1,024 × 1,024 using each dye’s optimal laser line settings. Image brightness and contrast were adjusted using Fiji ImageJ (Schindelin et al., 2012). Figures were created with Inkscape 1.3.2 (Inkscape project 2023).

## 3 Results

### 3.1 Detailed description of the pea aphid neuropeptide inventory

Several neuropeptides and protein hormone precursor sequences have been previously described in the pea aphid using genome mining (Huygens et al., 2021). However, since then, several novel neuropeptides have been described in other species and our approach required that the list of precursor sequences be as complete as possible.

As a first step, we assembled and analysed several publicly available transcriptomic datasets from different body parts of the pea aphid to complement incomplete or missing neuropeptide precursors (BLAST searches). Using already described pea aphid precursor sequences (Colizzi et al., 2023; Huybrechts et al., 2010), as well as those of other insects (Habenstein et al., 2021; Predel et al., 2018; Ragionieri et al., 2022), we compiled a list of 31 neuropeptides and 14 protein hormone sequences (Supplementary Files S1,S2), some of which have been described for the first time in the pea aphid. For example, we identified homologous precursors for HanSolin and Carausius Neuropeptide-like Precursor (CNP1), which have been recently discovered in stick insects (Liessem et al., 2018) as well as other insects (Veenstra, 2019; Bläser and Predel, 2020; Veenstra, 2023). In aphids, the HanSolin precursor contained an 8 AA long predicted HanSolin peptide with the typical C-terminal motif (GQPLRWa). Similarly, to other insects, the mature peptide is N-terminally flanked by a monobasic and C-terminally dibasic Arg cleavage site. The CNP1 precursor is 239 AA long and contains several peptides flanked by mono and dibasic cleavage sites, including two C-terminally amidated peptides. Another neuropeptide currently referred to as PeaNPLP1, has recently been identified in *Periplaneta americana* (Zeng et al., 2021) and *Bombus terrestris* (Ragionieri, 2025) and was detected in *corpora cardiaca* and *corpora allata*, which suggests its role as a neurohormone. In aphids, the PeaNPLP precursor contains a 11 AA long peptide (KK.SIDWSNYFGYD.RK), flanked on both ends by typical cleavage sites, however, the aphid precursor does not have a signal peptide, suggesting that the peptide is unlikely to be processed through the classical secretion pathway. We also identified precursors for RYa (Hauser et al., 2010; Roller et al., 2016), IDL-containing (Hummon et al., 2006; Kotwica-Rolinska et al., 2020; Marciniak et al., 2020), natalisin (Jiang et al., 2013), NVP-like peptide (Boerjan et al., 2010; Hummon et al., 2006; Jiang et al., 2013; Kotwica-Rolinska et al., 2020; Marciniak et al., 2020), and PDF in our BLAST searches. The latter mentioned was identified using the most recently described sequence for PDF of the pea aphid, since conventional BLAST searches using the PDF sequence of closely related hemiptera species did not result in identification of PDF, probably due to considerable sequence variation of the aphid precursor (Colizzi et al., 2023). Similar variations in the PDF sequence have also been observed, for example, in coleopteran species (Veenstra, 2019). We also complemented precursors for CCHamide1 (CCHa1), CCHamide2 (CCHa2) and Pyrokinin (PK), or missing neuropeptide precursors (Supplementary File S1). In general, all precursor sequences were very consistent to the ones described by (Huybrechts et al., 2010). Precursors for ET, AT, AST-CC, CCAP, and Agatoxin showed slight amino acid variations, however, those were outside of the mature peptide coding sequence.

We did not find neuropeptide precursors for Corazonin, Sulfakinin, Adipokinetic hormone/corazonin related peptide (ACP), Calcitonin A and B, CNMamide, Elevenin, IMFamide, OK_b_, Tryptopyrokinin, RFLamide, and pyrokinin-like, suggesting their absence in pea aphids. For ILPs, 11 precursor sequences have been proposed by genome mining (Huygens et al., 2021). In our transcriptomic analysis, we did not find transcripts for ILP7-10 which were predicted by Huybrechts et al. (2010) with the exception of an incomplete fragment of the ILP7 precursor (SRR9209839, Supplementary File S2). Moreover, transcripts for ILP2 and ILP3 were missing from our assemblies. Hence, those precursor sequences are not listed in Supplementary File S2.

Together, these transcriptomic analyses complemented the set of aphid neuropeptide precursors necessary for subsequent validation of mature peptide products using mass spectrometry.

### 3.2 Confirmation of expressed mature neuropeptides in the aphid brain via mass spectrometry

**FIGURE 2:**
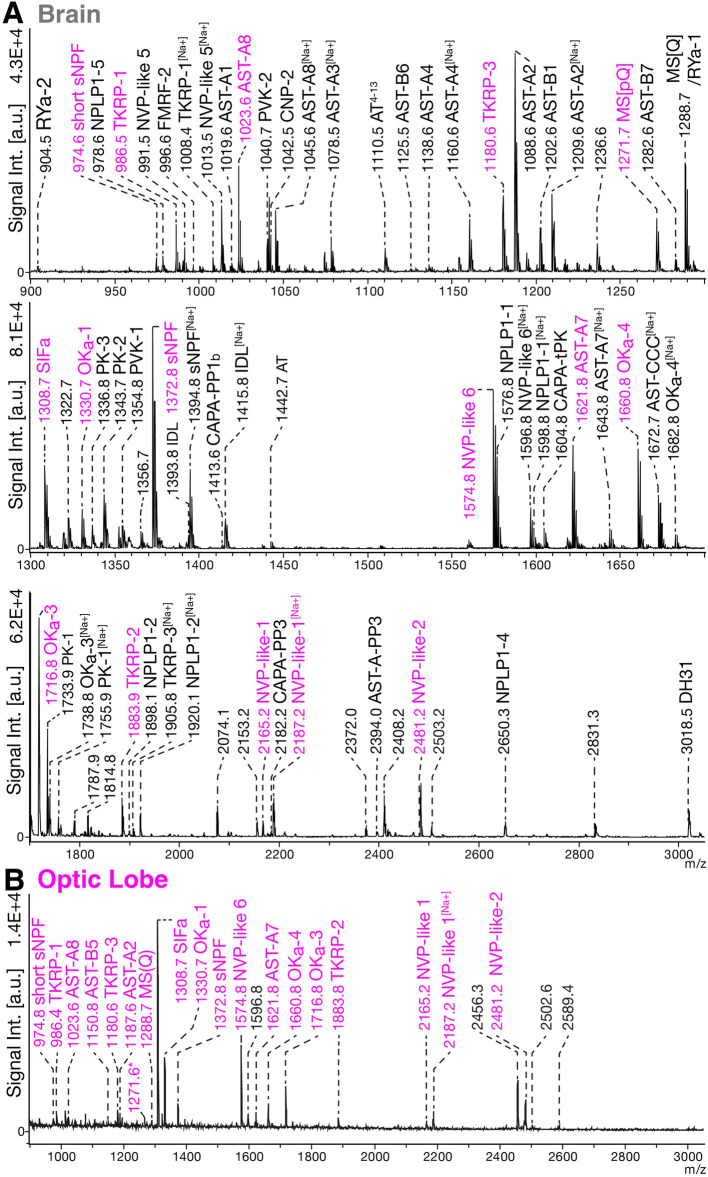
MALDI-TOF MS^1^ spectra obtained by direct tissue profiling of **(A)** brain and **(B)** optic lobe in the mass range *m/z* 900–3,100. Peptides that are abundant in Optic Lobe are labeled in magenta. For detailed comparison, see Supplementary File S3. Unless otherwise indicated, all marked ion signals represent single-charged peptide products ([M + H]^+^) from neuropeptide precursors. Uncapitalized letters following the peptide label indicate that peptides are from different precursor transcripts. [M + Na]^+^ and [M + K]^+^ labels salt adduct ions. *: *m/z* 1271.6 pyroglutamate form (pQ) of MS.

From our transcriptomic analysis we predicted 92 mature neuropeptides and 21 further precursor peptides (PP). To determine the actual set of processed neuroactive compounds including post-translational modifications, we analyzed brain extracts of the pea aphid by Q-Exactive Orbitrap MS and individual neuronal tissue samples by direct tissue profiling using MALDI-TOF MS, which combination has previously been successfully used to characterize the neuropeptidome of other insects (Liessem et al., 2018; Habenstein et al., 2021; Predel et al., 2018; Ragionieri and Predel, 2020). Using this approach, we were able to identify and confirm 49 mature bioactive neuropeptides by fragmentation analysis (Supplementary Files S3,S4), which was accompanied by the confirmation of 19 further PPs. These experiments confirmed the presence of mature products from the majority of neuropeptide genes (Supplementary Files S3,S4). For most of the precursors containing a single neuropeptide (AKH, AT, DH31, Myosuppressin (MS), sNPF, and SIFa) and other precursors containing multiple neuropeptides (ASTA, MIP, CNP-1, CAPA, Neuropeptide-like precursor 1 (NPLP-1), NVP-like, OK_a_, PK, and TKRP) most, if not all, of the predicted and likely also bioactive neuropeptides could be identified. For some precursors containing multiple copies of peptides such as FMRFa only a few peptides could be confirmed via fragmentation. Sequence identifications of mature bioactive peptides for 15 precursors (AKH, AST-CC, AST-CCC, CCHa1, CCHa2, CRF-DH54, Crustacean cardioactive peptide (CCAP), Ecdysis-triggering hormone (ETH), HanSolin, IDL-containing, LK, Neuropeptide F (NPF), Proctolin, RYa, and PDF) and all precursors containing larger protein hormones (bursicon alpha and beta, EH, GPA, GPB, ILP1-10, ITP and ITP-like) could not be confirmed from the brain extracts using mass spectrometry. The latter is likely due to the fact that they need further enzymatic digestion before detection or are simply outside of the analysis range. In the next step, we performed direct tissue profiling of neuropeptides by MALDI-TOF MS which is particularly suitable for a fast assessment of abundant neuropeptides in a specific tissue and, with it, provides first hints for the functional relevance of these peptides in that tissue. To that end, we performed our analysis on the central brain and optic lobes, the latter housing the lamina and lateral aphid clock neurons, to localize peptides identified by Q Exactive Orbitrap MS experiments to these compartments. This approach allowed us to identify the peptides present in these two macro regions of the aphid brain (protocerebrum and optic lobes) and to generate a list of candidate peptides for being clock-related. As expected, a large number of ion signals of neuropeptides could be detected in mass spectra from preparations of brain tissue (Figure 2A). In total, the resulting peptidomics data revealed 92 potential bioactive peptides (not including larger protein hormones) and 19 PPs by mass matches. Moreover, 68 peptides were confirmed by subsequent fragmentation analysis (Supplementary Files S3,S4), hence confirming the identity of these peptides. In contrast to a previous mass spectrometric analysis of the pea aphid CNS in which none of the peptides have been confirmed by fragmentation, our analysis revealed the presence of an additional 45 peptide mass matches. Direct tissue profiling of the OL revealed peptide products from the sNPF, TKRP, AstA, SIFa, OK_a_, and NVP-like precursor (Figure 2B), some of which can also be detected in the brain (magenta). Moreover, in our Q Exactive Orbitrap MS experiments several peptides from the CNP, MIP, MS, and NPLP precursors were confirmed. Some peptides, such as AT, showed very low signal intensities in brain samples despite confirmation by fragmentation. In the optic lobes, however, an amidated AT fragment was confirmed (Supplementary File S3), suggesting that AT may also be abundant in that part of the brain.

Together, this expanded and curated neuropeptide inventory provides a comprehensive basis for all subsequent peptidomic validation and anatomical localization of neuropeptide expression in circadian clock neurons.

### 3.3 Confirmation of expressed neuropeptides in the clock neurons of aphids by immunohistochemistry

The results from mass spectrometry showed that several neuropeptides found in the clock neurons of fruit flies are completely absent in aphids (e.g., ITP, NPF, AstC), while aphids possess several neuropeptides associated with the clock of cockroaches (e.g., MIP, OK_a_, FMRFa, and AT), but not with the clock of flies. Therefore, we used immunohistochemistry to investigate the presence of the latter neuropeptides in the clock neurons of aphids. In addition, we used antisera against typical fly clock neuropeptides that appear present in aphid brains such as AstA, DH31 and CCHa1. To pinpoint their location (or their absence) in the lamina (LaNs), lateral (LNs) and dorsal clock neurons (DNs), we performed fluorescent double labelling with the relevant neuropeptide antisera and antisera against the clock proteins PER and/or CRY as well as the neuropeptide PDF. As an internal reference and to assess the overall conservation of neuropeptide expression and antibody specificity in aphid neurons, we additionally used an antiserum against the highly conserved neuropeptide SIFa, which is consistently expressed in non-clock neurons of the central nervous system across insect species.

#### 3.3.1 The lamina clock neurons contain AT and CCHa1

**FIGURE 3:**
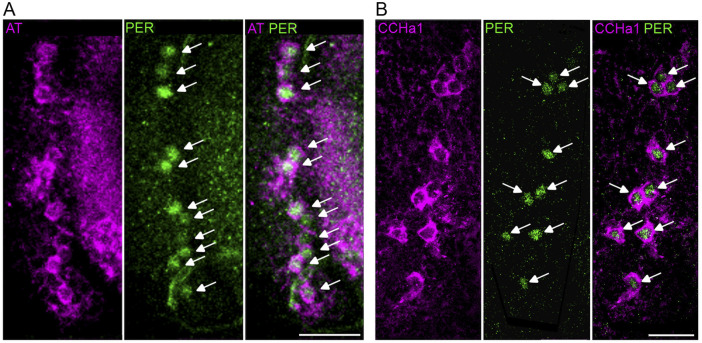
The lamina neurons contain AT and CCHa1. **(A)** Colocalization between AT (magenta) and PER (green) in all LaNs. **(B)** Colocalization between CCHa1 (magenta) and PER (green) in all LaNs. White arrows indicate the neurons in which the signals from both channels overlap. Maximum projection of eight image stacks. Scale bars 20 µm. AT, Allatotropin; CCHa1,CCHamide-1; PER, Period.

In aphids, the LaNs represent a distinct subset of clock neurons located in the optic lobes. Immunohistochemical analyses revealed that all LaNs express the neuropeptides AT and CCHa1 (Figure 3). Consistent with the peptidomic data, AT was detected by mass spectrometry in the central brain, and AT fractions were confirmed in the optic lobes (Supplementary File S3). Immunostaining revealed numerous AT-positive neurons in these regions. Notably, all LaNs were consistently AT-immunoreactive (Figure 3A), identifying AT as a prominent neuropeptide marker of this clock neuron population. In addition to LaNs, AT expression was also observed in PDF-positive LNs (see below). In contrast, CCHa1-derived peptides could not be confirmed by mass spectrometry in either brain or optic lobe extracts. Nevertheless, robust CCHa1 immunoreactivity was detected in all LaNs (Figure 3B), confirming the localization of CCHa1 in LaNs.

#### 3.3.2 Subsets of PDF-positive lateral neurons contain additionally AT, OK_a_, and FMRFa

**FIGURE 4:**
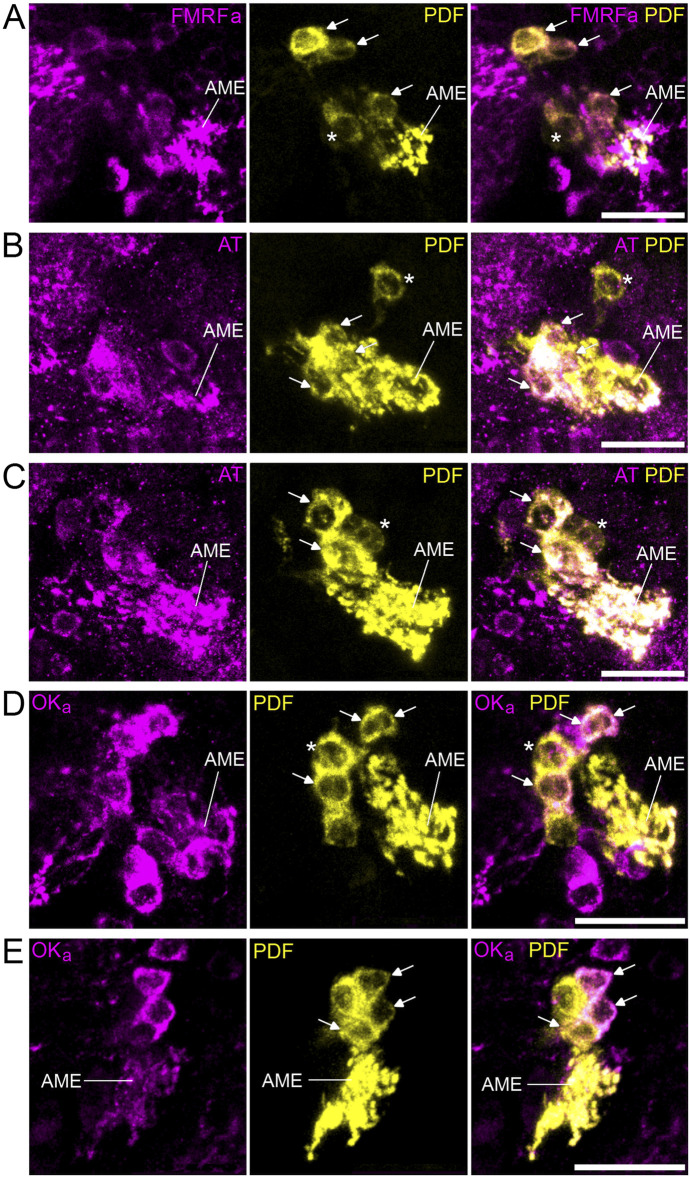
FMRFa, AT, and OK_a_ are colocalized with PDF in the lateral neurons and accessory medulla. **(A)** Colocalization between FMRFa (magenta) and PDF (yellow) in three LNs. **(B,C)** Two representative examples of colocalization between PDF (yellow) and AT (magenta) in three LNs. **(D,E)** Two representative examples of colocalization between PDF (yellow) and OK_a_ (magenta) in two and three LNs, respectively. White arrows indicate the LNs in which the signals from both channels overlap. Neurons stained only for PDF are marked by an asterisk. Maximum projection of 5–7 image stacks. AME: Accessory medulla. Scale bars: 20 µm. FMRFa, FMRFamide; PDF, Pigment-dispersing Factor; OKa, Orcokinin a; LNs, Lateral Neurons.

The four PDF-positive LNs of aphids appear to be extraordinarily rich in neuropeptides. 3 to 4 were detected using an antibody against FMRFa, three using an antibody against AT and three using an antibody against OK_a_ (Figure 4). We conclude that all four LNs contain PDF and FMRFa, but it is unclear whether the three OK_a_-positive LNs are identical with the three AT-positive LNs, because both antisera were raised in rabbits making a double-labeling difficult. Nevertheless, at least two of the LNs must contain PDF, FMRFa, AT and OK_a_. For the same reason, we also do not know whether the one CRY-positive, but PDF-negative LNs contains OK_a_ or FMRFa. This is possible, because more than three OK_a_ or FMRFa-positive neurons are present close to the AME (Figures 4A,D,E).

In the cockroach *Rhyparobia maderae*, OK_a_ is expressed together with FMRFa in three medium-sized PDF-positive clock neurons (Soehler et al., 2008). AT is also present in about 25 AME-neurons of the cockroach, but it is neither co-localized with PDF nor with OK_a_ (Hofer and Homberg, 2006). As the cockroach possesses many more putative clock neurons that are associated with the AME as compared to the aphid (Stengl and Arendt, 2016) it is possible that the aphid compensates for the fewer neurons by co-expressing more neuropeptides in individual cells.

**FIGURE 5:**
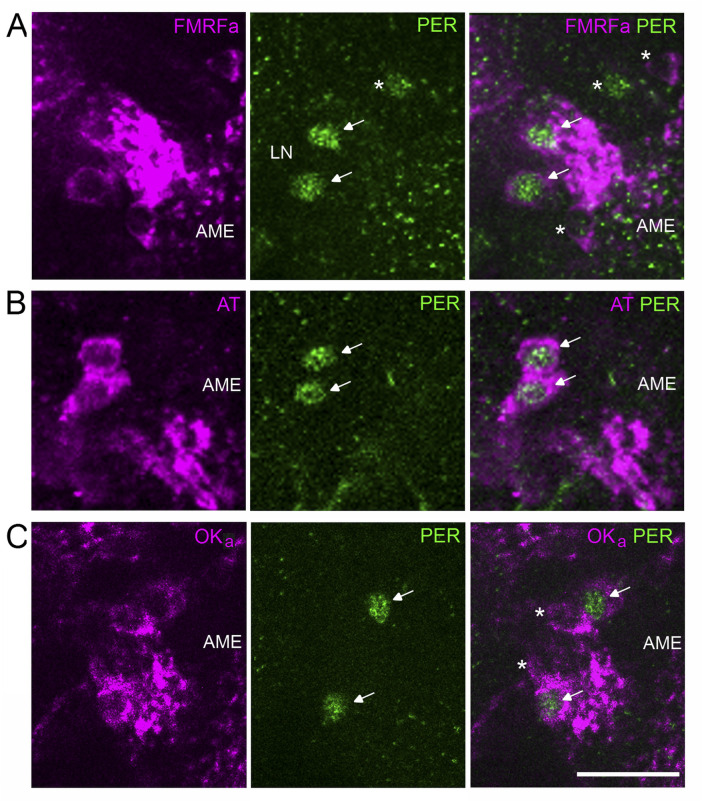
FMRFa, AT, and OK_a_ are colocalized with PER in at least two lateral neurons. **(A)** Colocalization between PER (green) and FMRFa (magenta) in two LNs. **(B)** Colocalization between PER (green) and AT (magenta) in two LNs. **(C)** Colocalization between PER (green) and OK_a_ (magenta) in two LNs. Only two or three of the five PER-positive neurons are visible at the time point of the staining. White arrows indicate the LNs in which the signals from both channels overlap. Neurons stained only for PER or only for the relevant neuropeptide are marked by an asterisk. Maximum projection of three confocal stacks are shown. AME: Accessory medulla. Scale bar: 20 µm.

Labeling with anti-PER did not reveal all LNs, most probably because PER oscillates in a daily manner (Colizzi et al., 2021) and has already decreased at the collection time of the present samples (ZT21-23). Therefore, the double-labeling with antibodies against the neuropeptides FMRFa, AT, and OK_a_ were not optimal to reveal a putative colocalization. Nevertheless, we could reliably detect two of the four LNs with anti-PER, and these two neurons were consistently double-labeled with the relevant neuropeptide antibody (Figure 5).

#### 3.3.3 The dorsal neurons contain FMRFa, MIP, AstA, and DH31, with AstA and DH31 restricted to CRY-negative neurons

**FIGURE 6:**
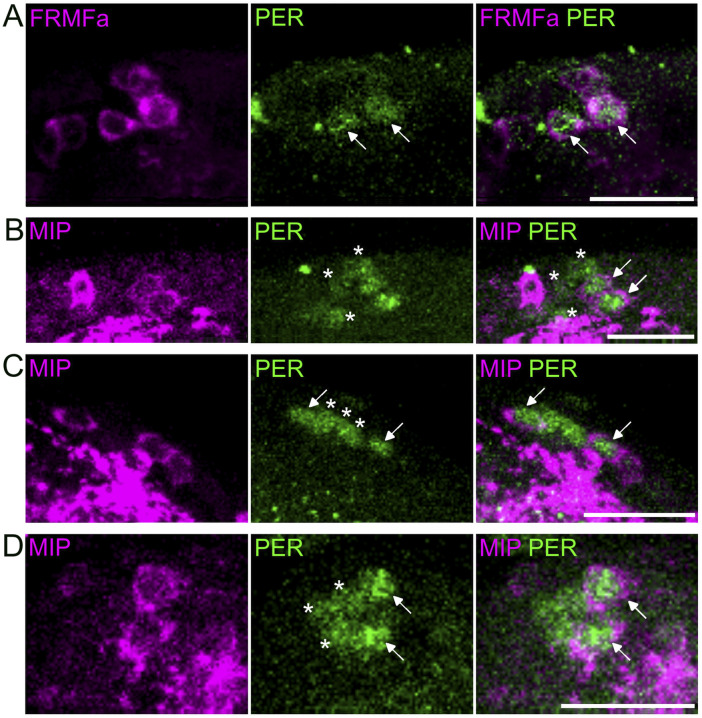
Two dorsal neurons contain FMRFa and MIP. **(A)** Colocalization between PER (green) and FMRFa (magenta) immunostaining in two DNs. **(B–D)** Representative examples of PER and MIP (magenta) double-labeling in the DNs of three separate brains. Neurons stained only for PER are marked by an asterisk. White arrows indicate the double-labeled neurons. Maximum projection of 3–5 confocal stacks. Scale bars: 20 µm. MIP, Myoinhibiting peptide.

FMRFa was detected not only in the PDF-positive LNs of the aphid, but also in at least two DNs (Figure 6A). Similarly, MIP colocalized with PER in two DNs (Figures 6B–D). However, because the primary antibodies, including anti-CRY, were all raised in rabbit, it was not possible to determine whether FMRFa and MIP are co-expressed in the same neurons or whether these neurons are CRY-positive. Nevertheless, we showed that neither FMRFa nor MIP do overlap with AstA in the DNs (see below). Based on this mutually exclusive labeling and the number and position of the cells, we therefore assume that FMRFa and MIP are colocalized in the same two CRY-positive neurons. Most interestingly, MIP was recently also detected in CRY-positive DNs of *Drosophila* (Reinhard et al., 2024), whereas in the Madeira cockroach it is colocalized with PDF in the AME neurons (Schulze et al., 2012).

**FIGURE 7:**
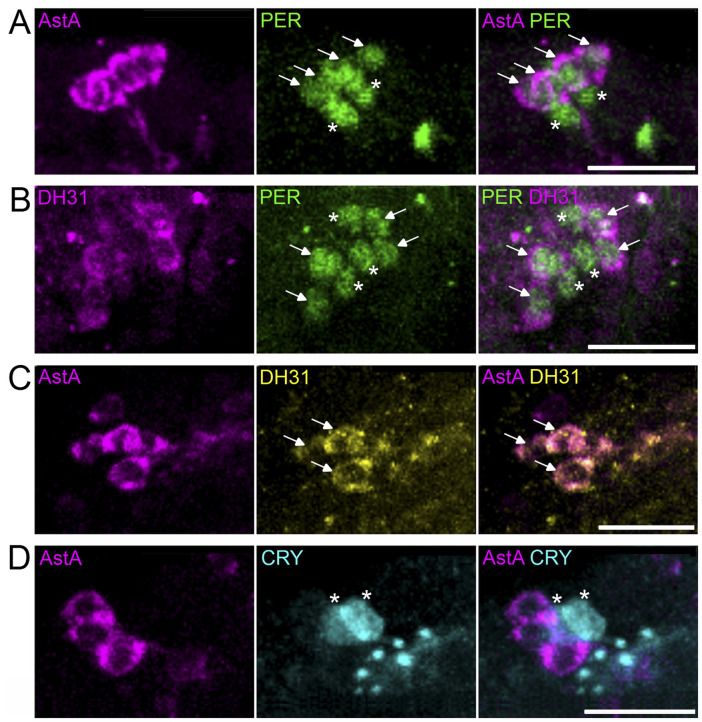
AstA and DH31 are present in the CRY-negative dorsal neurons: **(A–D)** Details of the DNs differentially labeled. **(A,B)** Colocalization between PER (green) and AstA or DH31 (magenta). **(C)** Colocalization between AstA (magenta) and DH31 (yellow). The two antisera label the same subset of DNs. **(D)** Colocalization between AstA (magenta) and CRY (cyan) shows that the anti-CRY antisera do not label the AstA-positive DNs. Neurons stained only for PER or only for CRY are marked by an asterisk. White arrows indicate PER-positive DNs not labelled by AstA or DH31 antisera. Projection of 3-6 image stacks. Scale bars 30 µm. AstA, Allatostatin A; DH31, Diuretic Hormone-31; CRY, Cryptochrome.

AstA and DH31 are both expressed in the fly clock neurons known as the lateral posterior neurons (LPNs), with DH31 also present in one group of the dorsal neurons (the DN_1p_) (Kunst et al., 2014; Reinhard et al., 2022). In aphids, we confirmed the presence of both neuropeptides in the central brain and optic lobes by mass spectrometry (Figure 2) and found by immunohistochemistry that both peptides are present in up to six DNs (Figure 7). For AstA, we used two antibodies raised in different host species, one in mouse and one in rabbit (Table 1). Both antibodies revealed the same distribution of AstA-positive cells, supporting the specificity of the AstA signal. In addition, the availability of the mouse anti-AstA antibody allowed us to perform double-labelling experiments with other rabbit-raised primary antibodies while avoiding overlap between antibody signals. To test whether DNs are CRY-positive, we conducted double-labelling with anti-AstA and anti-CRY antisera, which revealed that the AstA- and DH31-positive neurons are all CRY-negative (Figure 7D). These findings suggest that the DNs cluster is composed of two CRY-positive clock neurons that also contain MIP and FMRFa, and a group of five to six CRY-negative neurons that are positive for AstA, and DH31. Taken together, the neuropeptide composition of the dorsal neurons in the aphid shows similarities to that reported for dorsal clock neurons in the fruit fly.

#### 3.3.4 AstA-positive dorsal neurons project toward the insulin-producing cells

**FIGURE 8:**
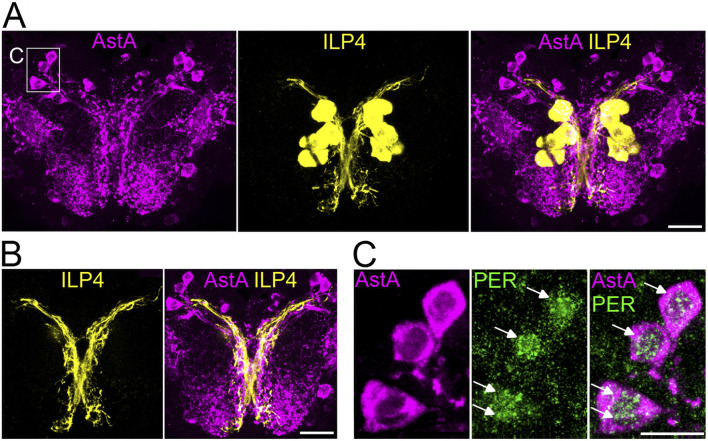
AstA-positive DN project towards the ILP4-positive IPC. **(A)** ILP4- and AstA-immunostaining in the *pars intercerebralis*. Maximum projections of 30 image stacks. Scale bar: 20 µm. **(B)** The lateral arborizations of the ILP4-positive cells (yellow) run very close to the AstA-positive axons (magenta). IPC: Insulin-Producing Cells. Maximum projection of eight image stacks. Scale bar: 20 µm. **(C)** Details of three DNs squared in panel **(A)**. Maximum projection of three image stacks. Scale bar 10 µm. ILP4, Insulin-like Peptide-4.

In *Drosophila*, AstA-positive neurons were shown to coordinate metabolism and feeding via IPCs in the *pars lateralis* (Hentze et al., 2015) and activation of AstA neurons affect IPC activity (Held et al., 2025). To investigate a potential link between AstA neurons and IPCs in aphids, we performed double-labeling experiments with AstA antisera with an antibody against ILP4, which has previously been shown to label aphid IPCs (Cuti et al., 2021; Colizzi et al., 2023). Our results revealed that the ILP4-positive and AstA-positive cell arborizations are in very close vicinity to each other, and some of the AstA-positive arborizations originate from the AstA-positive DNs (Figures 8A–C).

#### 3.3.5 CCHa1 is absent from the aphid dorsal clock neurons

In *Drosophila*, CCHa1 is present in dorsal neurons that are closely associated with the accessory medulla and it has an important role in the circadian clock (Fujiwara et al., 2018; Reinhard et al., 2025). Therefore, we examined its presence in the lateral and dorsal protocerebrum of the aphid by employing two different antisera, one directed against *Drosophila* CCHa1 and one against the precursor of aphid CCHa1 (Table 1). Both CCHa1 antisera yielded in principle similar results, however, the aphid antiserum labeled fewer neurons and showed a lower background, indicating higher specificity. We therefore base the following description on the stainings obtained with the aphid antiserum.

**FIGURE 9:**
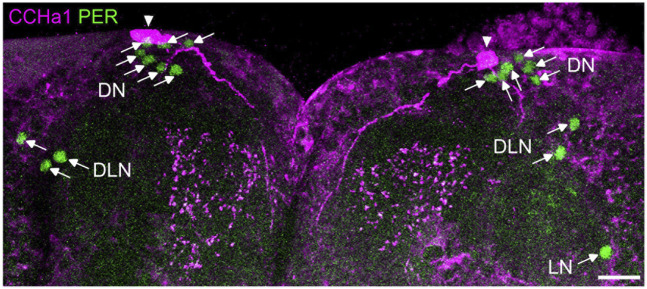
CCHa1 is absent from the DNs. The two CCHa1-positive neurons (magenta) are close to the DNs but do not colocalize with the DNs, DLNs, or LNs. White arrowhead indicates the CCHa1-positive neurons. Maximum projection of eight image stacks. Scale bars: 20 µm.

We found immunostaining for CCHa1 in 1-2 neurons in the dorsal brain, but there was no co-localization with the circadian clock neurons (Figure 9). The PER-positive neurons were in close vicinity to the CCHa1 positive neurons but were not identical with them.

In summary, we found clear differences but also similarities in the localization of neuropeptides in the clock neurons between flies, cockroaches, and aphids.

#### 3.3.6 SIFa immunoreactivity is conserved in aphids, and it labels four neurons in the *pars intercerebralis* that are distinct from those that are ILP4-positive

**FIGURE 10:**
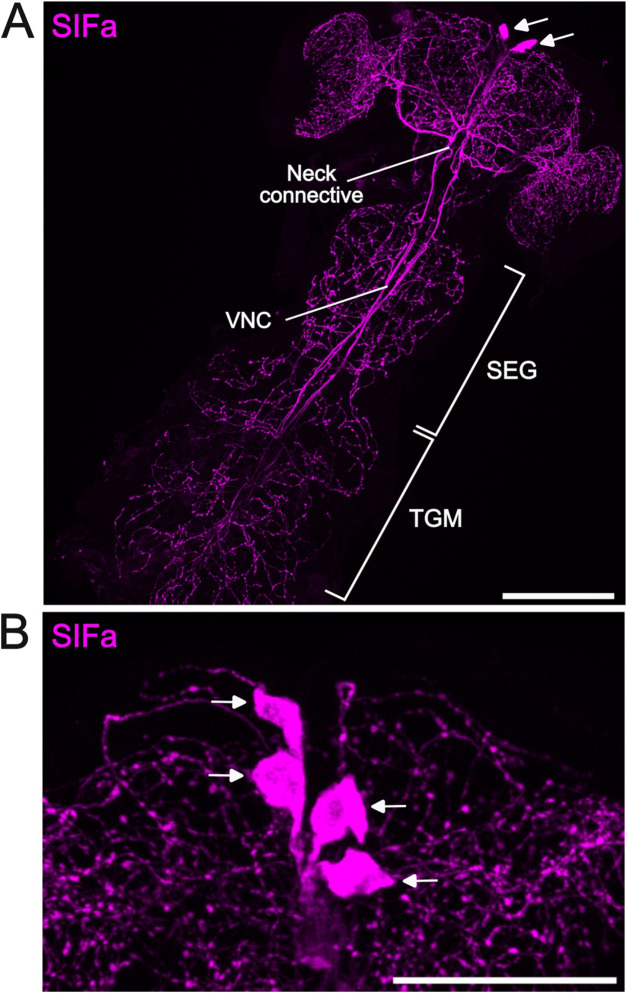
SIFa immunoreactivity in *A. pisum*. **(A)** Overview of SIFa immunoreactivity and 3D reconstruction of the major axons. White arrows indicate the SIFa-positive cell bodies. VNC: Ventral Nerve Cord. SEG: Subesophageal Ganglion. TGM: Thoracic Ganglion Mass. Maximum projection of 40 image-stacks. Scale bar: 100 µm. **(B)** Detail of the four immunoreactive cells located in the *pars intercerebralis*. White arrows indicate the SIFa-positive cell bodies. Maximum projection of 20 image-stacks. Scale bars: 50 µm. SIFa, SIFamide.

Given the pronounced differences observed in the neuropeptide composition of circadian clock neurons, we wanted to test whether other neuropeptides, such as SIFa, which have a rather conserved function, also show a different cellular distribution in aphids. SIFa is associated with the regulation of feeding, sexual behavior, and sleep by integrating hunger signals and modulating sensory circuits to promote appetite behavior and food intake (Nagata, 2016; Martelli et al., 2017; Ayub et al., 2020; Huang et al., 2021). In all insects tested, SIFa is consistently and exclusively contained in four neurons located in the *pars intercerebralis* (Carlsson et al., 2010; Heuer et al., 2012; Terhzaz et al., 2007). These neurons arborize extensively throughout the central brain, but do not innervate the neurohemal organ, *corpora cardiaca*. Consistent with other insects, the SIFa antisera labelled four neurons in aphids (two neurons per hemisphere) located within the *pars intercerebralis* region and vastly project throughout the entire brain (Figure 10A). Their arborizations branch laterally extending toward the optic lobes (Figure 10B), project posteriorly into the neck connective, innervating the ventral nerve cord, the subesophageal ganglia (SEG) and the thoracic ganglionic mass (TGM). SIFa immunoreactivity is very similar to that seen in other insects (Arendt et al., 2016; Carlsson et al., 2010; Heuer et al., 2012; Terhzaz et al., 2007) confirming the high degree of conservation of this neuropeptide in both distribution and peptide sequence.

**FIGURE 11:**
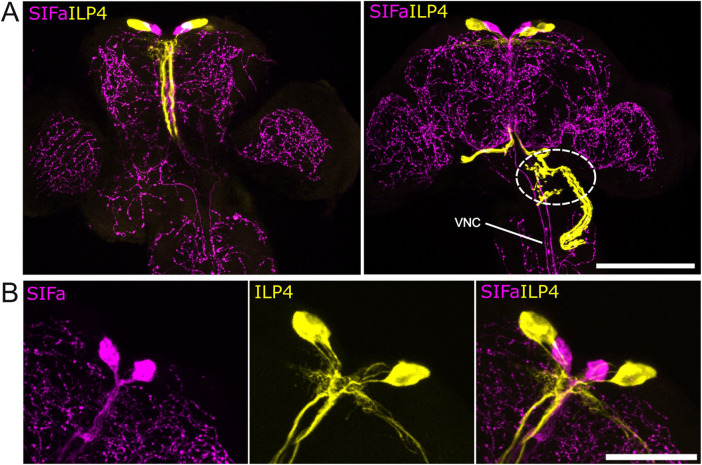
SIFa and ILP4 immunoreactivity in the central brain. **(A)** Ventral and dorsal stacks, left and right respectively, of SIFa (magenta) and ILP4 (yellow) immunoreactivity show that ILP4-ir cells run ventrally and exit dorsally towards the *corpora cardiaca*, while SIFa neurons run within the ventral nerve cord. The white circle indicates the region corresponding to the expected location of the corpora cardiaca, as previously described by Cuti et al. (2021). VNC: Ventral Nerve Cord. Projection of 25 image stacks. Scale bar: 100 µm. **(B)** ILP4- and SIFa-immunoreactive cells in the *pars intercerebralis*. Maximum projection of 12 image stacks. Scale bar: 50 µm.

In our previous study, and consistent with Cuti et al. (2021), we showed that eight IPCs expressing ILP4 are situated in the *pars intercerebralis*, which is the same region where we found SIFa-positive cells. To test whether SIFa and ILP4 are colocalized in the same neurons, we performed a double-labeling experiment and found that the two populations differ in both shape and location (Figure 11). SIFa-positive cells are smaller and positioned closer to the brain midline than the ILP4-positive neurons. The axons of ILP4-positive neurons first run ventrally and posteriorly; at the posterior end of the protocerebrum, they turn dorsally and exit the brain to innervate the corpora cardiaca, as supported by (Cuti et al., 2021). In contrast, the axons of SIFa-positive neurons extend posteriorly into the ventral nerve cord (Figure 11A).

## 4 Discussion

### 4.1 The neuropeptide inventory and absence of distinct neuropeptide signalling pathways in pea aphids

Neuropeptides are crucial modulators of connectivity within the clock networks and between the clock and the downstream pathways, including those involving neurosecretory cells that control photoperiodic responses (Helfrich-Förster et al., 2011; Wei et al., 2014; Stengl and Arendt, 2016; Taghert and Nitabach, 2012; Selcho et al., 2017; Hidalgo et al., 2023; Reinhard et al., 2024). To investigate neuropeptide function and localization, it is first necessary to identify the complete set of neuropeptides expressed (Neupert et al., 2025). In a previous study, some of these sequences have already been described in the pea aphid (Huybrechts et al., 2010), however, they were based on the genome, partially incomplete, and most importantly, novel neuropeptides have been described since then. Here, we compiled the complete set of pea aphid neuropeptides and protein hormone sequences based on similarity searches to other insects. Considering that aphid clock clusters are located in the central brain and in the lamina of the optic lobes (Colizzi et al., 2021), we also conducted mass spectrometry analysis (Q- Exactive Orbitrap MS and direct tissue profiling by MALDI- TOF MS) on these parts to characterize bioactive peptides processed from the predicted precursor proteins using two distinct mass spectrometric approaches.

In total, we compiled a list of 41 neuropeptides (Supplementary File S1) and 15 protein hormone precursor sequences (Supplementary File S2), some of which have been described for the first time in the pea aphid, such as RYamide (Rya), IDL-containing (Hummon et al., 2006; Kotwica-Rolinska et al., 2020; Marciniak et al., 2020), Natalisin (Jiang et al., 2013), NVP-like peptide (Boerjan et al., 2010; Hummon et al., 2006; Kotwica-Rolinska et al., 2020; Marciniak et al., 2020), HanSolin (Liessem et al., 2018) and CNP1 (Liessem et al., 2018; Marciniak et al., 2022; Veenstra, 2023; Bläser and Predel, 2020; Habenstein et al., 2021) since their discovery in other insect species. The localization of these peptides within the CNS and their posttranslational modification suggests their functionality and that they are physiologically relevant, however, their modulatory action has yet to be determined. We did not find any of these peptides in the OL. In general, we did not detect mature peptide products for HanSolin which are expressed in the brain of stick insects and termites, as well as termite ovaries (Veenstra, 2023).

These novel peptides such as HanSolin were initially found using a novel approach described in (Liessem et al., 2018) without the need of typical similarity searches, but based on the combination of transcriptomics and mass spectrometry. Using the same pipeline, we confirmed all precursor sequences with expressed gene products that we identified using traditional BLAST searches, but no novel precursor sequences were found. Hence, we are confident that our list of precursor sequences is as complete as possible. However, it is well established by now that neuropeptide precursors are often duplicated or lost during insect evolution (Bläser and Predel, 2020; Li et al., 2008). In our transcriptomic analysis we did not find neuropeptide precursors transcripts for 10 known insect neuropeptide genes (Corazonin, Sulfakinin, Adipokinetic hormone/corazonin related peptide (ACP), Calcitonin A and B, CNMamide, Elevenin, IMFamide, Orcokinin_b_ (OK_b_), Tryptopyrokinin, RFLamide, and Pyrokinin-like). This absence of precursors could hardly be explained by variations in their spatial and temporal expression patterns, because our assembled transcriptomic database consisted of several body parts (whole body, brain, and gut) and developmental stages. This suggests the complete absence of these precursors in *A. pisum*. Furthermore, their absence is supported by analysis of the pea aphid genome (Huybrechts et al., 2010) and loss of neuropeptides such as Corazonin (Veenstra and Ida, 2014), Sulfakinin (Liessem et al., 2021), ACP (Ragionieri and Predel, 2020), Calcitonin (Li et al., 2008), CNMamide (Bläser and Predel, 2020), elevenin (Bläser and Predel, 2020), IMFamide (Veenstra, 2019), Tryptopyrokinin (Veenstra, 2019), and RFLa (Bläser and Predel, 2020) is commonly observed throughout insects. For each of these neuropeptides that appeared to be absent and with a confirmed receptor, we conducted additional BLAST searches and confirmed the concomitant absence of their G-protein coupled receptors except for cognate receptors for ACP (LOC114119520) and calcitonin (LOC100166340). This could either mean that the sequence variation of the ACP or calcitonin neuropeptide precursor is too high to be detected by conventional homology searches (as was the case for the aphid PDF precursor (Colizzi et al., 2023) or that ACP and calcitonin signalling might have recently been lost in this species. Interestingly, we did not detect transcripts for the OK_b_ precursors. OK_b_ is a result of alternative splicing of the *ok* gene and OK_a_ peptides were confirmed in our analysis. Unfortunately, the cognate OK_b_ receptor has not been described in any species, hence we were unable to conduct searches for this receptor in our transcriptomic database. The absence of OK_b_ in the pea aphid has already been suggested (Sterkel et al., 2012). Additional effort in conducting BLAST searches for OK_b_ in other transcriptomic and genomic databases revealed that the loss of OK_b_ could even be a general feature in Aphididae.

Two Allatostatin C-type precursors have already been described in aphids (AST-C and AST-CC) (Huybrechts et al., 2010). However, we found that the precursor designated AST-C is more similar to AST-CCC, because it has an *Ala* residue instead of a *Pro* residue in the disulfide bridge and a C-terminal amination site (Veenstra, 2016). We could not find any transcript matching the AST-C precursor, suggesting its absence in aphids. We also changed the designation from the CCHa1 precursor to CCHa2 and *vice versa* based on later publications (Ida et al., 2012; Veenstra and Ida, 2014). For the genes encoding MIP, AST-CC, AT, CAPA, CCHa1, CRF-DH54, NVP-like, and RYa, allelic differences and alternatively spliced mRNAs were found (see Supplementary File S2). For Insulin-like Peptides (ILP), a total of six precursor sequences were identified including ILP11, described as gonadulin (Veenstra, 2020; Huygens et al., 2022), which is in accordance to the genome annotation at NCBI (Annotation Release 103, 24 May 2021) (Li et al., 2019) and the list of ILPs by Huygens et al., (2022). We did not find transcripts for ILP2-3 and ILP7-10 which were predicted by Huybrechts et al. (2010) with the exception of an incomplete fragment of the ILP7 precursor (Supplementary File S2). So far, ILP2 and ILP3 have only been found in two transcriptomic datasets of pea aphids that were not included in our analysis. For example, ILP2-3 were detected only in males and ILP3 was confined to the salivary glands (Huygens et al., 2022).

By comparing the calculated peptide fragments with the resulting fragmentation pattern generated by MS^2^ of the ion of interest (see Supplementary Material), we identified the majority of the predicted, and likely bioactive, neuropeptides for most of the above described prepropeptide genes. In our mass spectrometric analysis, we were able to predict and identify 92 neuropeptides and 21 precursor-related peptides, from which more than half were confirmed by subsequent fragmentation. Several of the detected peptides such as PDF, sNPF, AstA, MIP, DH31, FMRFa, OK_a_, and AT have already been associated with the clock system of *Drosophila* and cockroaches (Soehler et al., 2008; Peschel and Helfrich-Förster, 2011; Arnold et al., 2020; Reinhard et al., 2022; Neupert et al., 2025). For some neuropeptide precursor (AKH, AST-CC, AST-CCC, CCHa1, CCHa2, CRF-DH54, CCAP, ETH, HanSolin, IDL-containing, LK, NPF, proctolin, RYa, and PDF) and all precursors containing larger protein hormones (bursicon alpha and beta, EH, GPA, GPB, ILPs, ITP and ITP-like), no mature products were confirmed by fragmentation analysis. This can be due to several reasons. One explanation is that most of the precursor products (e.g., NPF, bursicon) did not yield positive hits because their predicted ion mass is outside of the detection range, used in our experiments. Also, their production can be restricted to specific developmental time windows (e.g., EH (Horodyski et al., 2001; Truman and Riddiford, 1970), restricted to specific tissues (AKH (Gäde and Goldsworthy, 2003; Van Der Horst et al., 2001), ETH (Roller et al., 2010; Žitňan et al., 1996), or further sample processing is necessary for detection, for example, a reduction of disulphide bonds (ILPs, GPA and GPB) (Boerjan et al., 2010; Ida et al., 2012; Liessem et al., 2021; Marciniak et al., 2020; Ragionieri and Predel, 2020; Veenstra, 2016; Veenstra and Ida, 2014).

Mass spectrometry analysis revealed clear differences in the neuropeptidomic profiles of the optic lobe and the central brain. The central brain exhibited a higher number of detected ion signals, indicating greater neuropeptide diversity compared to the optic lobe. All neuropeptides identified in the optic lobe were also detected in the central brain, whereas several peptides were exclusively present in the central brain, hence, the optic lobe expresses a restricted subset of the aphid neuropeptides. This complexity is consistent with the role of insect neuropeptides and peptide hormones as modulatory signals in the central brain regulating a variety of physiological states and behaviors, as well as the modulation of sensory processing (Nässel, 2025; Nässel and Winther, 2010; Nässel and Zandawala, 2019). In contrast, the optic lobe mainly processes visual information, where rapid neurotransmission predominates, and neuromodulation appears to involve a restricted set of neuropeptides, consistent with the expression of a restricted subset of neuropeptides detected in this tissue (Nässel and Winther, 2010; Homberg, 2020).

It should be noted that neuropeptide profiles obtained from the central brain and the optic lobes are expected to reflect peptides expressed by DNs and LaNs, respectively. The accessory medulla and the closely associated LNs lie at the boundary between the optic lobe and the central brain; therefore, it is difficult to clearly assign peptides from this population to one tissue fraction or the other.

### 4.2 Neuropeptide distribution in pea aphid lateral clock neurons largely resembles that of cockroaches

**FIGURE 12:**
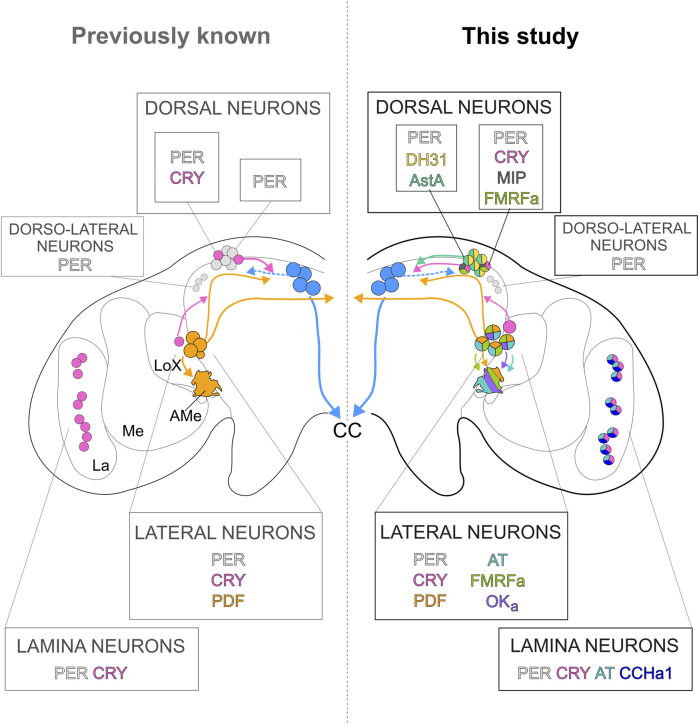
Schematic representation of neuropeptide inventory within different clusters of clock neurons in the aphid brain. Dorso-lateral clock neurons do not contain any of the neuropeptide tested.

Our final map of the neuropeptides in the aphid clock cells (Figure 12) has a unique pattern, while still sharing similarities with the established models of *Drosophila* and cockroaches.

Like in most other insects, the LNs of aphids express PDF and possess fibers that project to the AME (Colizzi et al., 2023), a crucial regulator of behavioral rhythms across insect species (Helfrich-Förster, 2004; Reischig and Stengl, 2003). Very similar to cockroaches, where the AME is well described and neuropeptides such as PDF, FMRFa, OK_a_, and AT are expressed in neurons and fibers associated with this neuropile (Arnold et al., 2020; Petri et al., 1995; Petri et al., 2002; Stengl et al., 2015) we also detected FMRFa, OK_a_, and AT-immunoreactivity in this region in aphids. However, the LNs of aphids do not express ITP, and NPF that are typical neuropeptides in the LNs of *Drosophila*.

Consequently, the composition of neuropeptides in the aphid LNs resembles much more the neuropeptide composition in the cockroach than the fly circadian system - and there are even more similarities: cockroaches, crickets and beetles possess Accessory Laminae (ALA) in addition to the AME (Stengl et al., 2015; Arnold et al., 2020; Fleissner et al., 2001; Tomioka, 2014; Helfrich-Förster, 2020). These ALA are located at the dorsal and ventral margin of the lamina and are - similarly to the AME - invaded by small clusters of neuropeptidergic lamina clock neurons. In cockroaches, the lamina neurons contain PDF, FMRFa and AT (Stengl et al., 2015; Arnold et al., 2020). Although aphids do not have PDF in the LaNs, they do show strong immunoreactivity for AT and CCHa1, and sparse immunoreactivity for FMRFa. Considering also that these neurons intrinsically express CRY1, it’s tentative to suggest that the LaNs in aphids are photoreceptive and send signals to the CRY1 negative clock neurons of the AME. Unfortunately, we could not see the projections of the aphid LaNs, but in cockroaches it was shown that the PDF, FMRFa and AT-positive lamina neurons project to the medulla and accessory medulla and are part of the light-input pathway to the circadian clock (Arnold et al., 2020). Further experiments will be crucial in aphids to verify these assumptions about the role of the LaNs in photic entrainment, and to define in more detail the neuroanatomy of the AME and ALA.

The DNs of aphids show more similarities in the peptidergic inventory with the fly DNs (Reinhard et al., 2024) than was the case for the LNs, while in cockroaches the dorsal clock neurons have not been identified making a comparison impossible. *Drosophila* possesses about 206 DNs, in which nine neuropeptides are expressed with maximally three to four neuropeptides co-expressed in the same cell (Reinhard et al., 2024). The latter we also observe in aphids, but aphids possess only seven DNs and express four different neuropeptides in them. Common to the DNs of both species are AstA, DH31 and MIP. Aphids have additionally FMRFa in their DNs, which is not present in flies, while flies express in addition AstC, DH44, CCHa1, CNMa, sNPF, and Proc, most of which appear absent in aphids.

In comparison to other insects, aphids generally possess much fewer clock neurons (Beer et al., 2018; Saifullah and Tomioka, 2003; Sandrelli et al., 2008). The aphid clock seems unusually compact, but whether this reflects a simpler circadian system (linked to aphid ecology, for example) or whether it is just a result of the limitation of the current investigation tools, remains an open question.

We show here that all DNs project towards the IPCs in the *pars intercerebralis*, which has long been established as a key center for photoperiodic regulation in aphids (Cuti et al., 2021; Lees, 1964), and our previous work has shown that the PDF-positive and the CRY-positive LNs project to this area (Colizzi et al., 2023). These findings, together with the identification of the neuropeptides present in the clock neurons and the advances made since our previous study, are summarized in the final schematic (Figure 12). Furthermore, the PDF-positive LNs show photoperiod-dependent changes in the length and staining intensity of their fibers in aphids and flies, suggesting that they convey photoperiodic information to the IPCs and modulate their activity in a season-dependent manner (Colizzi et al., 2023; Hidalgo et al., 2023). It is likely that the DNs contribute to seasonal regulation as well. At least in flies, the insulin-producing cells express the AstA receptor in addition to the PDF receptor (Reinhard et al., 2024). Future studies have to show whether the IPCs of aphids do also express the AstA and PDF receptors as well as the receptors for MIP, FMRFa, DH31.

## 5 Conclusion

This work represents a crucial step forward in the neurobiology of aphids, a field of research which remains largely understudied despite extensive research on the species in pest management. Functional studies with CRISPR-Cas9 to manipulate specific genes are now being established in aphids (Le Trionnaire et al., 2019; Fu et al., 2025; Shigenobu et al., 2025), providing the potential to investigate the role of these peptides in behavior and seasonal adaptation. In addition, by identifying neuropeptidergic pathways involved in circadian and photoperiodic regulation, these findings provide a basis for the design of neuropeptide mimetics to manipulate reproductive timing, for instance by inducing the production of sexual morphs outside their natural seasonal context, thereby potentially limiting the rapid population growth characteristic of aphids. Hence, by establishing the neuropeptidergic architecture of the aphid circadian clock, this study provides an essential foundation for future functional analyses and supports the development of targeted, peptide-based strategies for pest management (Konopińska et al., 2025) aimed at interfering with seasonal timing in this major agricultural pest.

## Data Availability

The original contributions presented in the study are publicly available. This data can be found in the NCBI SRA repository with the following accession numbers: SRR063706, PRJNA79725; SRR7037537, PRJNA450900; SRR9209839, PRJNA547535; SRR9209842, PRJNA547535; SRR12776646, PRJNA667506; SRR7037541, PRJNA450900, and at https://doi.org/10.6084/m9.figshare.32411367.
