# Supplementary material for: Clock-related neuropeptides in Acyrthosiphon pisum
Source: Front Genet. 2026 Jun 12;17:1818975. doi: 10.3389/fgene.2026.1818975 (PMC13302288; doi:10.3389/fgene.2026.1818975)
Supplement: Supplementary file 1 [file DataSheet1.pdf]

Supplementary Material

1 Supporting Information S1:

List of sequences of neuropeptide and neuropeptide-like precursors from *Acyrthosiphon pisum* transcriptome data. Underlined sequences are confirmed by MS^2. Varying amino acids at specific positions, that were not confirmed by mass spectrometry, are listed in parentheses. Otherwise the respective precursors are listed back to back and amino acid substitutions are marked with red letters. Different transcripts are marked with subscript characters (e.g. AST-B_a, AST-B_b). Blue, predicted signal peptide; yellow, predicted bioactive neuropeptide; green, predicted C-terminal glycine amidation site of neuropeptide; red, predicted cleavage site of neuropeptides; orange, predicted Cys-bridge site; '-', stop codon.

Adipokinetic hormone (AKH)

MRTLLLLAVFMLCACIAVGQVNFTPTWGQGKRNAPASDECKSMDTLIYIYKLVQVKYVFR
VFSLLIS-

Agatoxin-like Peptide

MKYFVTIFMVFHISLILLQKTSTASPLDNYLDKDSIEENGVELLTDIEEYMDKDADDNGFALD
FPDVQKRKLSSHRIFRRYCVPRGENCDHRPKYCCNSSSCRCNLWGVNCKCQRMGFFQRWG
K-

Allatostatin A (AST-A)

MHHSCCMWILVIATAVWTDAITGHEDKVGIKSQQAQQQQSDIMQTMVDGGGHQSIQMTS
PAESYFNDPLGPLGYLAKRAHKQYGFGLGKRLYRQYEFGLGKRSASKQYGFGLGKRAALK
QYEFGLGKRASPTFYSFGLGRRASPQYSFGLGKRVSHPSFLNVDDRESDYTYNDLSEEKKRT
ADDMGHGQRFAFGLGKRGAGAEWDDGDGDGDDAAPIWHPAVRRARLQYGFGLGKRADR
DYDATTGTEYTDTLQLADDAADINN-

Allatostatin B a (AST-B a, MIP)

MYSSFKRYNLFKHILFILLLKKNPSLCSNDIICFIILFNSDYTRSFDEDQEEKRAWRDLQTAGW
GKRGWQNLKTTWGKRAQDWQNLHSSWGKRQGWQKLHGGWGKRGWKDMQSGGWGKR
FKDQPASSQLSQFDEYLDKYEEENPNEAEKRSWDNFQGSWGKRAADWTSFRGSWGKRNPV
DYMNEYSGYGDNDNYKAYIFPPGYNSYLPNFQTEYEK-

Allatostatin B b (AST-B b, MIP)

MQNVLGRIAATLVILCPVIVFTIPESAIQASSIKSSQTEQDNSDYTRSFDEDQEEKRAWRDLQT
AGWGKRGWQNLKTTWGKRAQDWQNLHSSWGKRQGWQKLHGGWGKRGWKDMQSGGW

GKRFKDQPASGQLSQFDEYLDKYEEENPNEAEKRSWDNFQGSWGKRAADWTSFRGSWGK
RNPVDYMNEYSGYGDNDNYKAYIFPPGYNSYLPNFQTEYEK-

Allatostatin CC a (AST-CC)

MVLWNKIVLFLISLCMVALCDSAFQKPLDPFTLLRYDKRTPEDQPSNNEKNISVEFDEYPVIV
PKRTALLLDRLMVALQKAVDGNNSGNMKGYYPERSIPISGAPRPSPGVSMELQRRNQQKGR
LYWRCYFNAVSCFK-

Allatostatin CC b (AST-CC)

MVLWNKIVLFLISLCMVALCDSAFQKPLDPFTLLRYDKRTPEDQPSNNEKNISVEFDEYPVIV
PKRTALLLDRLMVALQKAVDGNNSGNMKGYYPERSIPISGAPRPSPGMELQRRNQQKGRLY
WRCYFNAVSCFK-

Allatostatin CCC (AST-CCC)

MATQMGFLSYGIILTLTVLTVLLPLLTTGNVIDQRVLQRELGENSPEIMSMSDKDVFRDIEEP
AIDDFTIKTKSEDQNLEIALIDYLFAKQMMNRIRARTDSFRAQKKRSYWKQCAFNAVSCFG-

Allatotropin a (AT a)

MAVNNNIMVRLLVIEITFLILAVVNSYPAFEDSEFKHKHRDKGRTIRGFKNMDLSTARGFGK
RTDHYMNLMPLDLFVDNKEDSFNQNIPMEVSLEKILKNKYKHFIEKLIDVNHDGYISGEELL
LSIDGES-

Allatotropin b (AT b)

MAVNNNIMVRLLVIEITFLILAVVNSYPAFEDSEFKHKNRDKGRTIRGFKNMDLSTARGFGK
RTDHYMNLMPLDLFVRSEERRVGKEC-

Calcitonin-like Diuretic Hormone (DH31)

MFTGNMMVGASVACGLLVVILVCTIPASLSAPYPLLQGQNNAYLSSENDGDPEVMLELLAR
IGQNIMRVNELENSKRGLDLGLSRGYSGTQAAKHLMGMAAANFAGGPGRRRRSDMLPKLL
TP-

***Carausius* Neuropeptide-like Precursor (CNP1)**

MYWNAVLLMAWSLATVSAILDSTRSQRYWDDTKPDEAGRYPNDNMLKTYMVRGGGGGN
KHFVTSVFPEMDARGFHEYVFDGLQPDFQWPSIKFKRNYYPQGITSRGFSDDIFHQSFGMFEP
LKRSVSRTSPSVANMLRRLYPNTGGIRLRQPDEHAPGSTAGKRRPEMDANGFHGDSFTGGF
DRFDTMKRRPEMDETGFEGESFTGFGGFETMKRNDGKNPARPASSAHAGRMSGARQE-

CAPA a

MKNLQTQIAAALLLTLTFFFTHALRHGDSEYSDEYKRDNSRDRRESAVAGLIPFPRVGRSGIN
SALQMDNLYETQRELRSHKREGLIPFPRIGRRSESKNTALWFGPRLGRSVVIPESYDTSYLDS
DTPTIIKTLIEMKSKNFGDEDDSLM-

CAPA b

MKNLQTQIAAALLLTLTFFFTHALRHDSEYSDEYKRDNSRDRRESAVAGLIPFPRVGRSGINS
ALQMDNLYETQRELRSHKREGLIPFPRIGRRSESKNTALWFGPRLGRSVVIPESYDTSYLDSD
TPTIIKTLIEMKSKNFGDEDDSLM-

CCHamide-1 a (CCHa-1 a)

MHKFFVQIYIFVLIIWAVEKSDCKQGAACLNYGHSCWGAHGKRNVPNDLDSLIRYRMAVFK
KSGHRDSFINPNADQSQEDIPNYYNIFKHYSKINSVKTNNDDTVDTWSLEPSNNLPSGGSYYE
DQVLDPRIEYKIMKI-

CCHamide-1 b (CCHa-1 b)

MHKFFVQIYIFVLIIWAVEKSDCKQGACLNYGHSCWGAHGKRNVPNDLDSLIRYRMAVFKK
SGHRDSFINPNADQSQEDIPNYYNIFKHYSKINSVKTNNDDTVDTWSLEPSNNLPSGGSYYED
QVLDPRIEYKIMKI-

CCHamide-2 (CCHa-2)

MYSITMPLRPQIATKCYFCMVLLAIVLIFTVDNGAAKRGCAMFGHSCYGAHGKRSFQIPIQQ
PARDWPATRREEETQIDDAINKYFKIKHFTPSPFWQKMVQMYNERKNNHLNNDSNM-

Corticotropin releasing factor-like DH54 a (CRF-DH54 a)

MRILTIAWILVAVTWCCDSAVIYDPVVQNTRYYEPRIQDLELLDKNFFDMASIEKRNGAMQ
GESPRSRPSLSIVNSLDVLRQKLMYEVARRHVDENQKVLSQNHQILKNLGKRSLFPFIEVPRR
YNVFRI-

Corticotropin releasing factor-like DH54 b (CRF-DH54 b)

MRILTIAWILVAVTWCCDSAVIYDPVVQNTRYYEPRIQDLELLDKNFFDMASIEKRNGAMQ
GESPRSRPSLSIVNSLDVLRQKLMYEVARRHVDENQKVLSQNHQILKNLGKRSLFPFIEVPRR
F-

Crustacean cardioactive peptide a (CCAP)

MFAQGHSSTAMNPSILTLVWMSILVSLVQTVFADDVIMQKRYFDNDNPVAEPIRRKKPFCN
AFTGCGRKRSDESMATLVDLRSEPAVEEISRQIMSEAKLWEAIQEARLELIRQQRQNKAERM
DVKPYPIGLRRKRRSLATSDKC-

Crustacean cardioactive peptide b (CCAP)

MNPSILTLVWMSILVSLVQTVFADDVIMQKRYFDNDNPVAEPIRRKKPFCNAFTGCGRKRSD
ESMATLVDLRSEPAVEEISRQIMSEAKLWEAIQEARLELIRQQRQNKAERMDVKPYPIGLRR
KRRSLATSDKC-

Ecdysis-triggering hormone (ETH)

MSGYLAIIVLVCLQILRVMSINEFPEKKVQNIWLADLDDKQIASRIERSDQFETASDVLMKDA
SVYPKITRRGFAGEEFFLKASKSVPRIGRRNNDIQETPKRSLSKLILGGFIYV-

Extended FMRFamide (FMRFa)

MLLCLLPVTLTLAALVTDGTGDTAAADKRFALRPVDPLTRRSAMDKNFMRFGRAFDCSWT
GPSTSAVAKRRDPSSAVGRRVDSNFIRFGRRDSNFIRFGRGEVYTPGDNKIPRRHYDVDVDG
LEVRFGRSAGSIDRSPFAAALPPPYDDRR-

HanSolin

MTLQWALLLVLVTTLIAAEKGSYEETGNDLLRWLDNSGRGFGTWRSPQEDEDEELEYPANI
FKNENRKRALSFLTHWRPWNQLSGNGRHVIRSPFSSFFPDTDIPSKGNRPVGQPLRWGRRRR
R-

IDL-containing

MARQQQLILTAAMILCCCGGGLIVSALDLNKLYGHIHAKRNIGEPCHPYEPFKCPGNGACISI
QYLCDGAPDCPDGYDEDARLCTAAKRPPVEETASFLQSLLASHGPNYLEKLFGSKARDALE
PLGGVEKVAITLSESQTIEDFGAALHLMRSDLEHLRSVFIAVENGDIGMLKSLGIKDSELGDV
KFFLEKLVNTGFLD-

Leucokinin (LK)

MVKIGLPWLLLVLMKLTNREIKSDEIISIQEVYDICQAEPNTDICDLLEKSSSLVDLNKLDSPE
KRRQKTVFSSWGGKRQSTYPYGGKRPAFSSWGGKRASDKHGRPKQTFSSWGGKRSDYDG
YDNGEMDEHQMDKRELNGIKQDKNNYRNKMTKGIHALFTIFSDWSRDPEEKKGIRYAGIKS
MRRSSDFFPWGGKRFTGDAK-

Myosuppressin (MS)

MSTYRTMLTSVLVLLVVASVCDCRINNLPTRCMPGLLEDAPPKVREACLTLSTIRTLSNAIET
FIQDKQFPMPMPYSRMSDGLVDTAMQNDKRQDLDHVFLRFGRRRR-

Natalisin

MELMDARTKLYLISVAYFVQLINGDARISRADISAVLGENDDPNFWPSRGRRNSPEPKFKQY
LERKYNMNTLNGNLQKPLYVDEPMWLTIDRRAEGDDDYFWVTRGRRGNSWKHPTSARLS
TNSNYNYRDESDNKIK-

Neuropeptide F (NPF)

MVRVVVYIGFVIYVMVLATTCCCHPVTSTEVESIARPTRPKTFGSPDELRSYLDQLGQYLAV
VSRPRFGKRKPTFPVIPSAITTPRLQQYIDHQRMLNNIINREDEEAYKLQYKPAAIRNSKDLYD
MLFTTRRENGNNDRHQYYLMQQDITNADIGLD-

Neuropeptide-like precursor 1 (NPLP1)

MKAATILAALACLLEVSHAFPFNFNKRNLDSLARTGQLPEYKRSLASLAKSGQLPEKLVQEK
RMLDVLEQASDANSKAIQDQKNRVIEEIKNLALTGLSKRQAATEPDFRDEFKSDLRSILDQFL
EKFGTTYDPEKVELFLTNMADEVFENHGVVSLDHIRFLIETGYFQPEMREVDDVKPADGDSS
PDDYI(T/A)KRFMASLARNDNMPFWYNSPRYVAKRYISSLLRQGRLPYGFQPTTESSTKNQN
WNLQDGQKPKRSDRPYQNSEEFIPVMQGSKTLQSIIDDLTQEPMQKRYLAGTSRILSGHLKN
SGGNTSSRRQQQNRRKQTAG-

NVP-like a

MNWFTSLVLSVAVAVVVTHMAHGLPAPGKADAAKIQKNMAKNKAKKTQEVLQFGNQQN
RQADTRNYARAEKRQAPDDHHDPEPVPEVNNALEHYPQSARHEKITDLFYNPDSYGIQSLD
ADDRYKRNAYHFGSPVKRSGSRVYGSYLDSGRAKRDLPFDPEELLALMSILEANKARDKSR
RPAQNYGQSYGQNYGHDTIPYEENEIPEEDDEDDVQEVWEERPVVLAPSPKDFYINNEFQRK
QRAQHGSKNNYFQGLNHYQEEPQYYRLSDFYRYYNY-

NVP-like b

MNWFTSLVLSVAVAVVVTHMAHGLPAPGKADAAKIQKNMAKNKAKKTQEVLQFGNQQN
RQADTRNYARAEKRQAPDDHHDPEPVPEVNNALEHYPQSARHEKITDLFYNPDSYGIQSLD
ADDRYKRNAYHFGSPVKRSGSRVYGSYLDSGRAKRDLPFDPEELLALMSILEANKARDKSR
RPAQNYGQSYGQNYGHDTIPYEENEIPEEDDEDDVQEVWEERPVVLAPSPKDFYINNEFQRK
QRAQHGSKGLNHYQEEPQYYRLSDFYRYYNY-

Orcokinin$_a$ (OK$_a$)

MASSSTMIVAVASALCVHTILAYPTSIERVSGDNNYLPLRNSPSRDLDRFIEGENLLRDLEILR
DRAEYFARQSRHINSLDGVGFGQSKRFDTLSGVSFGGQKRNFDEIDRSGFDRFVKKNFDEID
RSGFDRFVKKNFDEIDRSAFNSFVKRPNKVPAANLE-

Proctolin

MAGKFSALFLVGFVAAVVVAPYMMAEARYLPTRGNDDRLTRLKELLTDLLDSGAQPNLEM
ERPYVDVNGDFSRLRPREYNIPEKSIMELFNPTVPHHQRPRS-

Pyrokinin (PK)

MCTVCYLWFILACSLLMTNANILNDLQEAQKFMEQLDYDYSAVVADAAAAAAQQRPVAD
LLWYDYGGGSGISGGVGRGESGGGSSGAGYFGGGFPVTGVQATPLFGADKRGGTTQSSNG

GIWFGPRLGRRKRRGGSPFSGSVVHPVDGNAIGPAASSLLQQNPLAAGSTSAAAEQAAVSDL
INNVPWVLVPIIDNSLYNQIQMKQNARNGRSSEEDDDDVASRSRHSARSPPYSPPFSPRLGRQ
AVMAQPQVPRLGRETLLYRRDARNALYQQSNATLLKQQRQQQQQQQHSPQTAAESAAA
RRQAV-

RYamide a (RYa a)

MTMSVTITILCILGSTFLLIMPDNTTASDKFFQTGGRFGKRHDEHIPGEHTIDIRYAAMVKTRS
VDNVPPRIERGFYISRYGKRSTNSITDPYYFTLCLPSYGIYCDFTGLPNLLRCKRIQPGACSNL
NYVNEKTQMKPDNDIII-

RYamide b (RYa b)

MTMSVTITILCILGSTFLLIMPDNTTASDKFFQTGGRFGKRHDEHIPDIRYAAMVKTRSVDNV
PPRIERGFYISRYGKRSTNSITDPYYFTLCLPSYGIYCDFTGLPNLLRCKRIQPGACSNLNYVN
EKTQMKPDNDIII-

Short neuropeptide F (sNPF)

MKSIAAVVCTLLLVSTLISAAPSYMDYENAKDLYELLLQRDLIDSMGNSLIDPNQHRMVRK
NQRSPSLRLRFGRRSDPALYQATDYECVSV-

SIFamide (SIFa)

MNFKCTVAVFLLMVVLMFATDSTNGFRKPPFNGSIFGKRTISYPEYENPGKTIYTMCEIASDA
CQNWFPTSVEKK-

Tachykinin-related peptide (TKRP)

MPHKINVGLVALAALAAAVLADPSVDRRASMGFMGMRGKKDRDQGGGGSGGDETSAAV
DLDKRTMVFRRPMFDGGSRPAVFGGGSAEGFKRASMGFMGMRGKKDYYSNNKGSAAGFF
GMRGKKVPSADAFYGVRGKKWPDHEDAVDADVQLSPIYILYRIIDELKSELSDRERNLVAA
KFDEEREMR-

Pigment dispersing factor (PDF)

MKLLGHIHHLLLICAYLTYLIIMTNGYPLRTTYCSLYVPDDN(S/F)VIEEQNAPIATKPVLFFG
KRHVEDDSSNDLIKPSESNIILKKKNSEIISALMGLRRAMSNKYDARHIHN-

2 Supporting Information S2:

List of precursor sequences containing protein hormone sequences from *Acyrthosiphon pisum*. Blue, signal peptide; yellow, predicted bioactive neuropeptide; green, predicted C-terminal amidation site of neuropeptide; red, predicted cleavage site of neuropeptides; orange, predicted Cys-bridge site; underlined sequence, confirmed by MS^2.

Bursicon alpha

MSTINQEFFRYLTVLAMCSMAFADNGNGVVVTARSSDDCQVTPVIHVLQYPGCVPKPIPSFA
CTGRCSSYLQVSGSKIWQMERSCMCCQESGEREASVSLFCPKAKQGEKKFRKVTTKAPLEC
MCRPCTGIEESAVIPQEMSNYAADEPPINGHFSKSI-

Bursicon beta

MYTTQLFIISIAIIIVHNNVLAEDNPEECETLPSELHIIKEEFDELGRLQRTCNGDIAVNKCEGA
CNSQVQPSVITPSGFLKECYCCRETFLRERMIALTHCYDPDGVRLTSDKLATLEVKLKEPAD
CKCFKCGDFSG-

Eclosion hormone 1 (EH1)

MMNTP(Q/S)KKITFLAAVALVLIGIVGYTTADMADVAMCIRNCAQCKKMLGDYFEGPLCAD
TCVKFKGKMIPDCENIDSIAPFLNKLE-

Eclosion hormone 2 (EH2)

MNTVSRKIALLAAALVLIAIVGYAVADMADVGLCIRNCAQCKKMLGAYFEGPLCADACVK
FKGKMIPDCENIDSVAPFLNKLE-

Glycoprotein hormone alpha (GPA)

MILIFILAVLPSPLATGSNTWQKPGCHKVGHTRKISIPNCVEFPITTNACRGYCESWAVPSPAD
TVMINPHQRITSVGQCCNIMETENVCEQLYRIELEKCMRALHKKR-

Glycoprotein hormone beta (GPB)

MVCMAVLCAVSSMIVWSSVAGYDMLDCNRQLSTFQVSNTDENGRTCSDEIDVMSCWGRC
DSNEVSDWRFPFKRSHHPVCIHDGQVLTKFMLKNCEEGVQPGTEFYVFPQAMSCKCHTCKS
SEAACEGYRSKDFPFISPNQV-

Insulin-like peptide 1 (ILP1)

MKINIYLSSLLLTLVIKFVTSDLKLPPQQYCGSRLADIMQVVCKNRYNTPPPPIGQKRNKMDS
EVWDYKDLEDYNAIDYPYQPRQDSMSFMPTRILRSSKRTIIDECCRRPCLISELKSYCAN-

Insulin-like peptide 4 (ILP4)

MKISMYLSVILLALVIKVVTANVRLQRSPQQYCGSKLADIMKALCNTKYNVPKGHKRSEIDF
DVWDYKDLEDYNAVDYPYIKKEDISFMPSRFRRSVKRSIIDECCRRPCYLSELKSYCASQ-

Insulin-like peptide 5 (ILP5)

MNSAVVLVIVMLLASHVSSSPIINFSWDTPRHFCGSQLANVLALICSNGYNFHPATDDVTVPS
RRRKRVIVEECCENTCTPHHLKAYCWENRRR-

Insulin-like peptide 6 (ILP6)

MHRHYQNVVVIILTFNLMVDCAPRAPEVIEWHTQNHFCGSAIPIIMGLICKVPERIPPKQVED
TNRRVRRNIVDECCKAPCSLIYMQSYCHEDLEK-

Insulin-like peptide 7 (ILP7)

…IDNYCGSRLANELEVICKGKYNELPAGEEQISIGQRRRLRLPVIVDECCTLPCNRRTLKMY
CAPDA-

Insulin-like peptide 11 (ILP11)

MTKFTIVALVQAAVMFVMQVSVESCGDEFIKNMIVNVCGMGGIKRSFPDNSRPDSSLGLHF
RGMVTDVEREFKHLVGDIEEVDLLLKTSNNKNKLKEPNVTPLWLRPINLPGFRDDRSRHPRI
RGDSGKATIIKRELMNDFRECCNKNCSLKDLKRICGKK-

Ion transport peptide (ITP)

MGYEPALVLVATLIAIMAVSVVAVPAAHHLHHHSGGHHRMMSSGGAAGSSSPSLSGIDHPL
SKRSFFDIQCKGVYDKTIFARLDRVCEDCYNLFREPQLHSLCSRSTCFNTVYFKACLESLQML
MEETQYNQMVEFLGKK-

Ion transport-like peptide a (ITP-like)

MGYEPALVLVATLIAIMAVSVVAVPAAHHLHHHSGGHHRMMSSGGAAGSSSPSLSGIDHPL
SKRSFFDIQCKGVYDKTIFARLDRVCEDCYNLFREPQLHSLCSRKNCFTTDFFKGCLDVLLLE
DEVEKIQKWIKQLHGADPVGVRA-

Ion transport-like peptide b (ITP-like)

MGYEPALVLVATLIAIMAVSVVAVPAAHHLHHHSGGHHRMMSSGGAAGSSSPSLSGIDHPL
SKRSFFDIQCKGVYDKTIFARLDRVCEDCYNLFREPQLHSLCRKNCFTTDFFKGCLDVLLLED
EVEKIQKWIKQLHGADPVGVRA-

3 Supporting Information S3:

Summary of neuropeptides/neuropeptide-like peptides and additional precursor products from *A. pisum* which were identified by MS analyses (direct tissue profiling with MALDI-TOF MS including MS^2; extract analysis with Q Exactive Orbitrap MS). CB, central brain. OL, optic

lobes. Peptides from different transcripts are marked with subscript characters (e.g. CCHa1a, CCHa1b). Underlined letters, half of disulfide bridges. @, MS^1 or MS^2 obtained from salt adduct. Post-translational modifications such as pyroglutamate (pQ) and amidation (a) are indicated.

Designation	Sequence	m/z [M + H^+]	MALDI-TOF MS^1		MALDI-TOF MS^2	Q Exactive Orbitrap	
			CB	OL	CB/OL	CB	OL
**AKH**							
AKH	qQVNFTPTWGQa	1159.55	-	-		-	-
**AST-A**							
AST-A1	AHKQYGFGLa	1019.54	+	-		+	+
AST-A2	LYRQYEFGLa	1187.62	+	+	+	+	+
AST-A3	SASKQYGFGLa	1056.55	-	-		+	+
AST-A4	AALKQYEFGLa	1138.63	+	-		+	+
AST-A5	ASPTFYSFGLa	1088.54	+	-		+	+
AST-A6	ASPQYSFGLa	968.48	-	-		+	+
AST-A7	TADDMGHGQRFAFGLa	1621.75	+	+	+	+	+
AST-A8	ARLQYGFGLa	1023.57	+	+	+[@]	(+)	-
AST-A-PP1	VSHPSFLNVDD-OH	1229.58	-	-		+	-
AST-A-PP2	ESDYTYNDLSEE-OH	1464.56	-	-		+	-
AST-A-PP3	GAGAEWDDGDGDGDDAAPIWHPAV-OH	2393.99	+	-		+	-
**AST-B**							
AST-B1	AWRDLQTAGWa	1202.60	+	-		+	+
AST-B2	GWQNLKTTWa	1189.61	-	-		+	-
AST-B3	AQDWQNLHSSWa	1370.62	-	-		+	+
AST-B4	QGWQKLHGGWa	1195.61	-	-		-	-
AST-B4 (pQ)	pQGWQKLHGGWa	1178.59	-	-		-	-
AST-B5	GWKDMQSGGWa	1150.51	-	+		+	+
AST-B6	SWDNFQGSWa	1125.47	+	-		+	-
AST-B7	AADWTSFRGSWa	1282.60	+	+		-	-
**AST-CC**							
AST-CC	NQQKGRLYWRCYFNAVSCF-OH	2381.12	-	-		-	-
**AST-CCC**							
AST-CCC	SYWKQCAFNAVSCFa	1650.74	+[@]	-		-	-
**AT**							
AT	GFKNMDLSTARGFa	1442.72	+	-		-	-
AT^{4-14}	NMDLSTARGFa	1110.54	+	-		+	-
**CAPA**							

PVK-1	ESAVAGLIPFPRVa	1354.78	+	-		-	-
PVK-2	EGLIPFPRIa	1040.63	+	-		-	-
CAPA-tPK	SESKNTALWFGPRLa	1604.85	+	-		+	-
CAPA-PP1$_a$	LRHGDSEYSDEY-OH	1470.61	-	-		-	-
CAPA-PP1$_b$	LRHDSEYSDEY-OH	1413.60	+	-		+	+
CAPA-PP1$_b^{3-11}$	HGDSEYSDEY-OH	1201.43	-	-		+	-
CAPA-PP2	SGINSALQMDNLYETQREL-OH	2182.05	+	-		+	-
**CCAP**							
CCAP	PFCNAFTGCa	956.4	-	-		-	-
CCAP-PP1	YFDNDNPVAEPIR-OH	1549.73	-	-		+	-
CCAP-PP1$^{1-12}$	YFDNDNPVAEPI-OH	1393.63	+	-		+	+
CCAP-PP2	SDESMATLVDL-OH	1180.54	-	-		+	-
CCAP-PP3	QNKAERMDVKPYPIGL-OH	1858.99	-	-		+	-
CCAP-PP3(pQ)	pQNKAERMDVKPYPIGL-OH	1841.96	-	-		+	-
**CCHa-1**							
CCHa1$_a$	KQGAACLNYGHSCWGAHa	1799.78	-	-		-	-
CCHa1$_b$	KQGACLNYGHSCWGAHa	1728.75	-	-		-	-
**CCHa-2**							
CCHa2	KRGCAMFGHSCYGAHa	1621.7	-	-		-	-
**CNP1**							
CNP-1	NYYPQGITS-OH	1042.48	+	-		+	+
CNP-2	LRQPDEHAPGSTAa	1377.69	-	-		+	+
CNP-3	SVSRTSPSVANML-OH	1348.69	-	-		+	+
CNP-4	PEMDANGFHGDSFTGGFDRFDTM-OH	2551.03	-	-		+	+
CNP-5	NDGKNPARPASSAHAa	1491.74	-	-		(+)	-
**CRF-DH54**							
CRF-DH54	NGAMQGESPRSRPSLSIVNSLDVLRQKLMYEVARRHVDENQKVLSQNHQILKNLa	6195.3	-	-		-	-
**DH31**							

DH31	GLDLGLSRGYSGTQ AAKHLMGMAAANFA GGPa	3018.50	+	-		+	-
DH31-PP	SDMLPKLLTP-OH	1114.62	+	-		+	-
**ETH**							
ETH	GFAGEEFFLKASKS VPRIa	1982.10	-	-		-	-
**FMRF**							
FMRF-1	SAMDKNFMRFa	1245.59	-	-		+	-
FMRF-2	VDSNFIRFa	996.53	+	-		+	-
FMRF-3	DSNFIRFa	897.46	-	-		-	-
FMRF-4	HYDVDVDGLEVRFa	1562.76	-	-		-	-
**HanSolin**							
HS	PVGQPLRWa	951.55	-	-		-	-
**IDL**							
IDL	LDLNKLYGHIHA-OH	1393.76	+	-	+@	+	-
**LK**							
LK-1	QKTVFSSWGa	1038.54	-	-		-	-
LK-1(pQ)	pQKTVFSSWGa	1021.51	-	-		-	-
LK-2	QSTYPYGa	814.37	-	-		-	-
LK-2(pQ)	pQSTYPYGa	925.41	-	-		-	-
LK-3	QTFSSWGa	811.37	-	-		-	-
LK-3(pQ)	pQTFSSWGa	922.41	-	-		-	-
LK-4	SSDFFPWGa	941.42	-	-		-	-
**MS**							
MS(Q)	QDLDHVFLRFa	1288.68	+	-		+	+
MS(pQ)	pQDLDHVFLRFa	1271.65	+	+		+	-
**Natalisin**							
Natalisin-1	ADISAVLGENDDP NFWPSRa	2101.99	+	-		+	-
Natalisin-2	AEGDDDYFWVTR a	1472.64	+	-		-	-
Natalisin-PP1	GNSWKHPTSA	1084.52	-	-		+	-
**NPF1**							
NPF1	HPVTSTEVESIARP TRPKTFGSPDELRS YLDQLGQYLAVVSR PRFa	5099.68	-	-		-	-
**NPLP1**							

NPLP1-1	NLDSLARTGQLPEY-OH	1576.80	+	-		+	+
NPLP1-2	SLASLAKSGQLPEKLVQE-OH	1898.10	+	-		+	+
NPLP1-3	MLDVLEQASDANSKAIQDQKNRVIEEIKNLALTGLS-OH	3955.08	(+)	-		-	-
NPLP1-4	FMASLARNDNMPFWYNSPRYVA-OH	2650.23	+	-		-	-
NPLP1-5	YISSLLRQa	978.57	+	-		-	-
NPLP1-6	LPYGFQPTTESSTKNQNWNLQDGQKP-OH	2978.43	-	-		+	-
NPLP1-7	SDRPYQNSEEFIPVMQGSKTLQSIIDDLTQEPMQ-OH	3924.86	+	-		-	-
NPLP1-8	YLAGTSRILSGHLKNSGGNTSS-OH	2220.14	-	-		-	-
**NVP-like**							
NVP-like-1	LPAPGKADAAKIQKNMAKNKA-OH	2165.22	+	+		+	+
NVP-like-2	TQEVLQFGNQQNRQADTRNYA-OH	2481.19	+	+		+	+
NVP-like-3	pQAPDDHHDPEPVPEVNNALEHYPQSA-OH	2889.27	-	-		+	+
NVP-like-4	HEKITDLFYNPDSYGIQSLDADDRY-OH	2975.37	-	-		-	-
NVP-like-5	NAYHFGSPV-OH	991.46	+	+	+	+	+
NVP-like-6	SGSRVYGSYLDSGRA-OH	1574.76	+	+		+	+
NVP-like-7	DLPFDPEELLALMSILEANKARDKS-OH	2815.46	-	-		-	-
NVP-like-8	PAQNYGQSYGQNYGHDTIPYEENEIPEEDDEDDVQEVWEERPVVLAPSPKDFYINNEFQ-OH	6946.07	-	-		-	-
NVP-like-9$_a$	QRAQHGSKNNYFQGLNHYQEEPQYYRLSDFYRYYNY-OH	4640.12	-	-		-	-
NVP-like-9$_b$	QRAQHGSKGLNHYQEEPQYYRLSDFYRYYNY-OH	3973.84	-	-		-	-
**OK$_a$**							
OK$_a$-1	HINSLDGVGFGQS-OH	1330.64	+	+		+	+

OK$_a$-2	FDTLSGVSFGGQ-OH	1214.57	-	-		+	+
OK$_a$-3	NFDEIDRSGFDRFV-OH	1716.80	+	+		+	+
OK$_a$-4	NFDEIDRSAFNSFV-OH	1660.76	+	+	+	+	+
OK$_a$-5	PNKVPAANLE-OH	1052.57	-	-		+	+
**Proctolin**							
Proctolin	RYLPT-OH	649.37	+	-		-	-
**PK**							
PK-1	GGTTQSSNGGIWFGPRLa	1733.87	+	-		+	-
PK-2	SPPYSPPFSPRLa	1343.71	+	-		+	+
PK-3	QAVMAQPQVPRLa	1336.75	+	-		+	-
PK-PP1	SSEEDDDDVASRSRHSA-OH	1862.77	-	-		+	-
**RYa**							
RYa-1	SDKFFQTGGRFa	1288.64	+	(+)		-	-
RYa-2	GFYISRYa	904.47	+	-		+	-
RYa-PP1	HDEHIPDIRYAAMVKT-OH	1895.94	-	-		+	-
RYa-PP2	SVDNVPPRIE-OH	1125.59	+	-		+	-
**sNPF**							
sNPF	NQRSPSLRLRFa	1372.79	+	+	+	-	-
short sNPF	SPSLRLRFa	974.59	+	+	-	-	-
**SIFa**							
SIFa	FRKPPFNGSIFa	1308.72		+	(+)	-	-
**TKRP**							
TKRP-1	ASMGFMGMRa	986.43	+	+	+	+	-
TKRP-2	DYYSNNKGSAAGFFGMRa	1883.85	+	+	+	+	-
TKRP-3	VPSADAFYGVRa	1180.61	+	+		+	+
TKRP-PP1	DRDQGGGGSGGDETSAAVDLD-OH	1978.82	-	+		+	+
TKRP-PP2	PMFDGGSRPAVFGGGSAEGF-OH	1942.88	-	-		+	-
**PDF**							
PDF-PP	HVEDDSSNDLIKPSESNIIL-OH	2225.09	-	-		+	+
PDF	NSEIISALMGL-OH	1147.6	-	-		-	-
**EH1**							

EH1	DMADVAMCIRNCAQCKKMLGDYFEGPLCADTCVKFKGKMIPDCENIDSIAPFLNKLE-OH	6338.92	-	-		-	-
**EH2**							
EH2	DMADVGLCIRNCAQCKKMLGAYFEGPLCADACVKFKGKMIPDCENIDSVAPFLNKLE-OH	6218.93	-	-		-	-

4 Supporting Information S4:

Fragmentation spectra of peptides detected in Orbitrap analysis. Upper panels, intensity of fragments. Middle panels, mass error range. Lower panels, Confirmed b and y fragments (colored) and general information about the MS scan.

4.1 AST-A

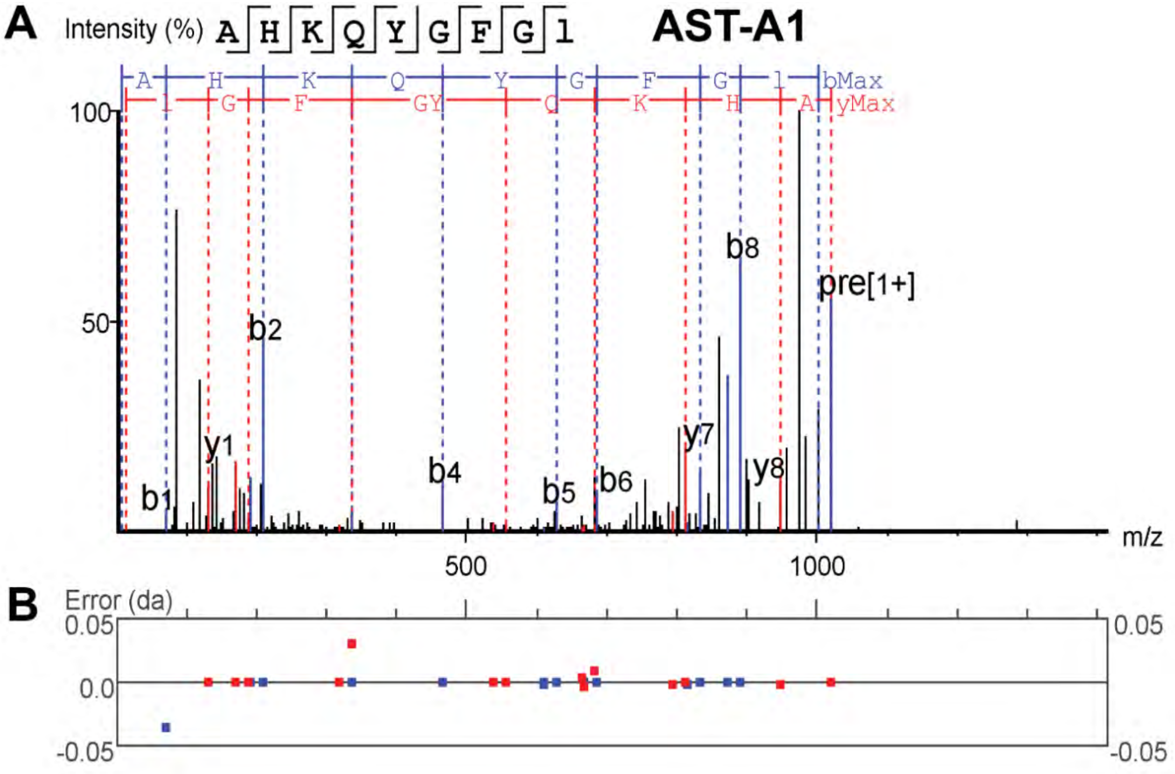

C **Ion Table**

#	b	b-H2O	b-NH3	b (2+)	Seq	y	y-H2O	y-NH3	y (2+)	#
1	72.08	54.03	55.02	36.52	A					9
2	209.10	191.09	192.08	105.05	H	948.51	930.49	931.48	474.75	8
3	337.20	319.19	320.17	169.10	K	811.45	793.44	794.42	406.22	7
4	465.26	447.25	448.23	233.13	Q	683.34	665.34	666.33	342.18	6
5	628.32	610.31	611.29	314.66	Y	555.29	537.28	538.27	278.15	5
6	685.34	667.33	668.32	343.17	G	392.23	374.22	375.20	196.61	4
7	832.41	814.40	815.39	416.71	F	335.18	317.20	318.18	168.10	3
8	889.43	871.42	872.41	445.22	G	188.14	170.13	171.11	94.57	2
9					L(-.98)	131.12	113.11	114.09	66.06	1

-10lgP: 61.78; Mass: 1018.5348; Length: 9; ppm: 0.0; m/z: 510.2747; z: 2; RT: 40.28 ; Scan: 7703;

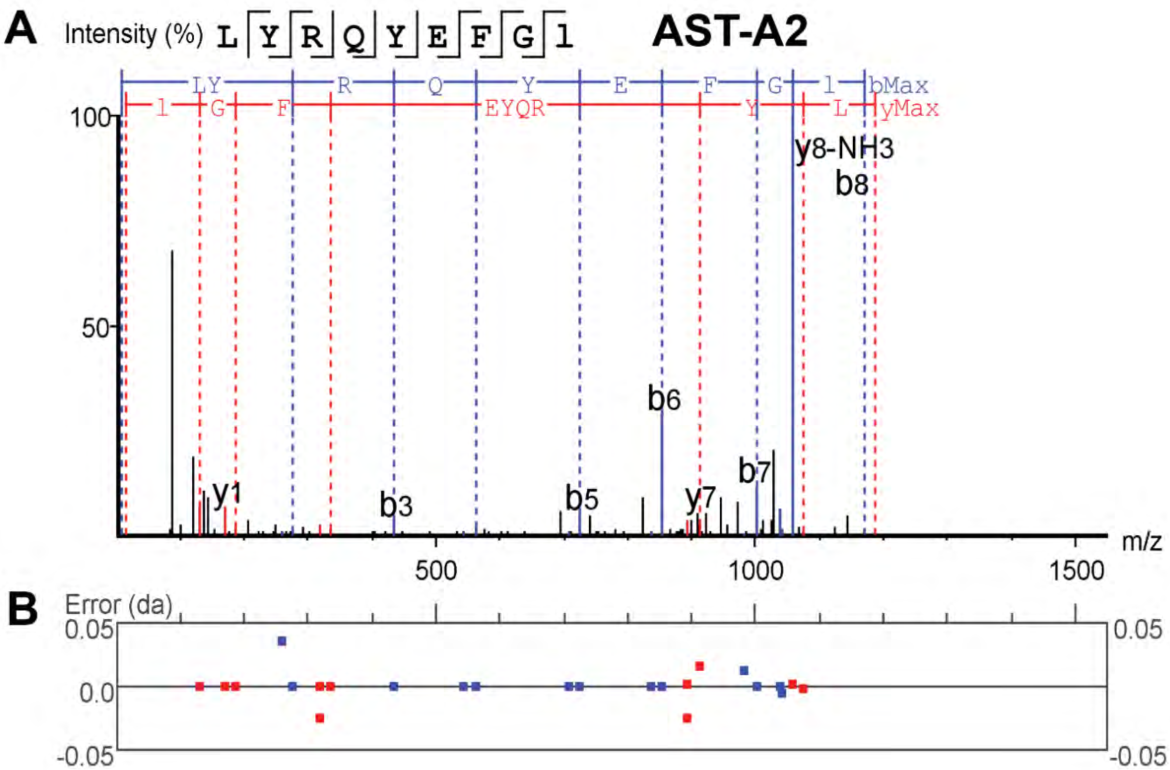

C Ion Table

#	b	b-H2O	b-NH3	b (2+)	Seq	y	y-H2O	y-NH3	y (2+)	#
1	114.09	96.08	97.06	57.55	L					9
2	277.15	259.11	260.13	139.08	Y	1074.54	1056.53	1057.51	537.77	8
3	433.26	415.25	416.23	217.13	R	911.46	893.49	894.44	456.24	7
4	561.31	543.30	544.29	281.16	Q	755.37	737.36	738.35	378.19	6
5	724.38	706.37	707.35	362.69	Y	627.31	609.30	610.29	314.16	5
6	853.42	835.41	836.39	427.21	E	464.25	446.24	447.22	232.63	4
7	1000.49	982.47	983.46	500.74	F	335.21	317.22	318.18	168.10	3
8	1057.51	1039.50	1040.49	529.26	G	188.14	170.13	171.11	94.57	2
9					L(-.98)	131.12	113.11	114.09	66.06	1

-10lgP: 53.50; Mass: 1186.6134; Length: 9; ppm: -0.2; m/z: 594.3138; z: 2; RT: 53.04 ; Scan: 10453;

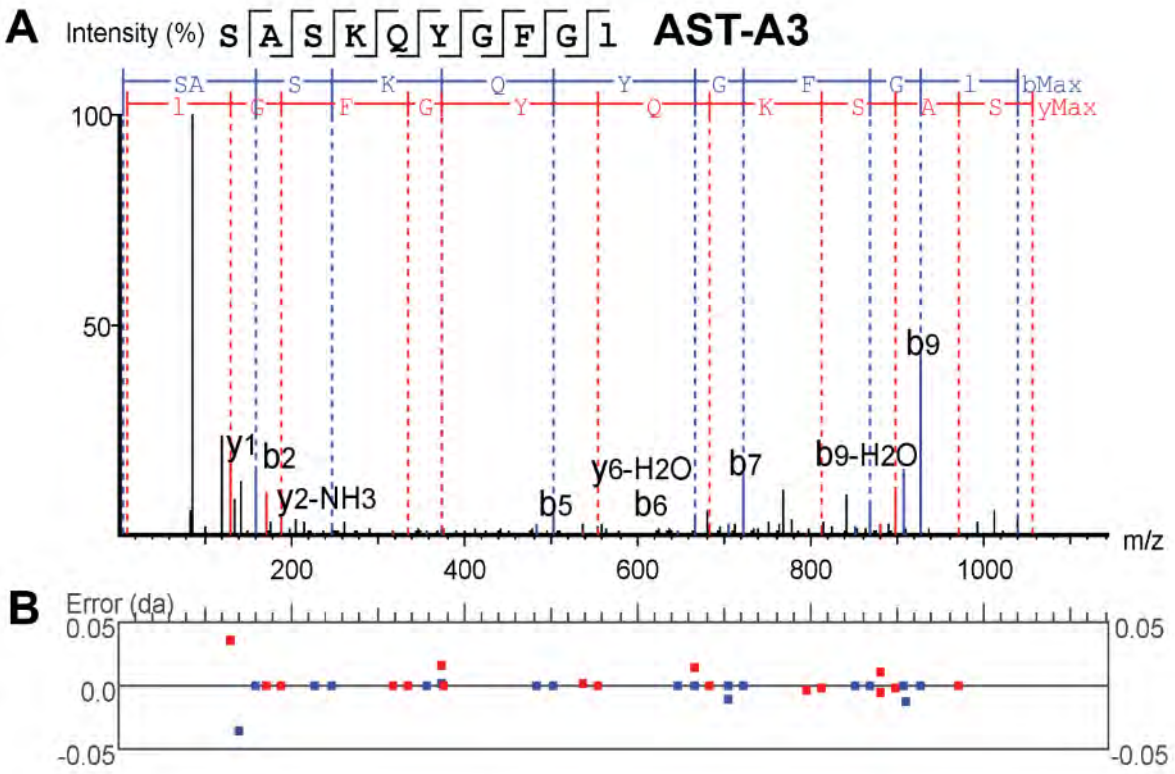

C

Ion Table

#	b	b-H2O	b-NH3	b (2+)	Seq	y	y-H2O	y-NH3	y (2+)	#
1	88.04	70.03	71.01	44.52	S					10
2	159.08	141.10	142.05	80.04	A	969.52	951.50	952.49	485.26	9
3	246.11	228.10	229.08	123.55	S	898.48	880.46	881.46	449.74	8
4	374.20	356.19	357.18	187.60	K	811.45	793.44	794.42	406.22	7
5	502.26	484.25	485.24	251.63	Q	683.35	665.33	666.32	342.18	6
6	665.33	647.31	648.30	333.16	Y	555.29	537.28	538.26	278.15	5
7	722.35	704.34	705.33	361.67	G	392.23	374.20	375.20	196.61	4
8	869.41	851.41	852.39	435.21	F	335.21	317.20	318.18	168.10	3
9	926.44	908.43	909.42	463.72	G	188.14	170.13	171.11	94.57	2
10					L(-.98)	131.08	113.11	114.09	66.06	1

-10lgP: 55.78; Mass: 967.4763; Length: 9; ppm: -0.1; m/z: 484.7454; z: 2; RT: 55.18 ; Scan: 10980;

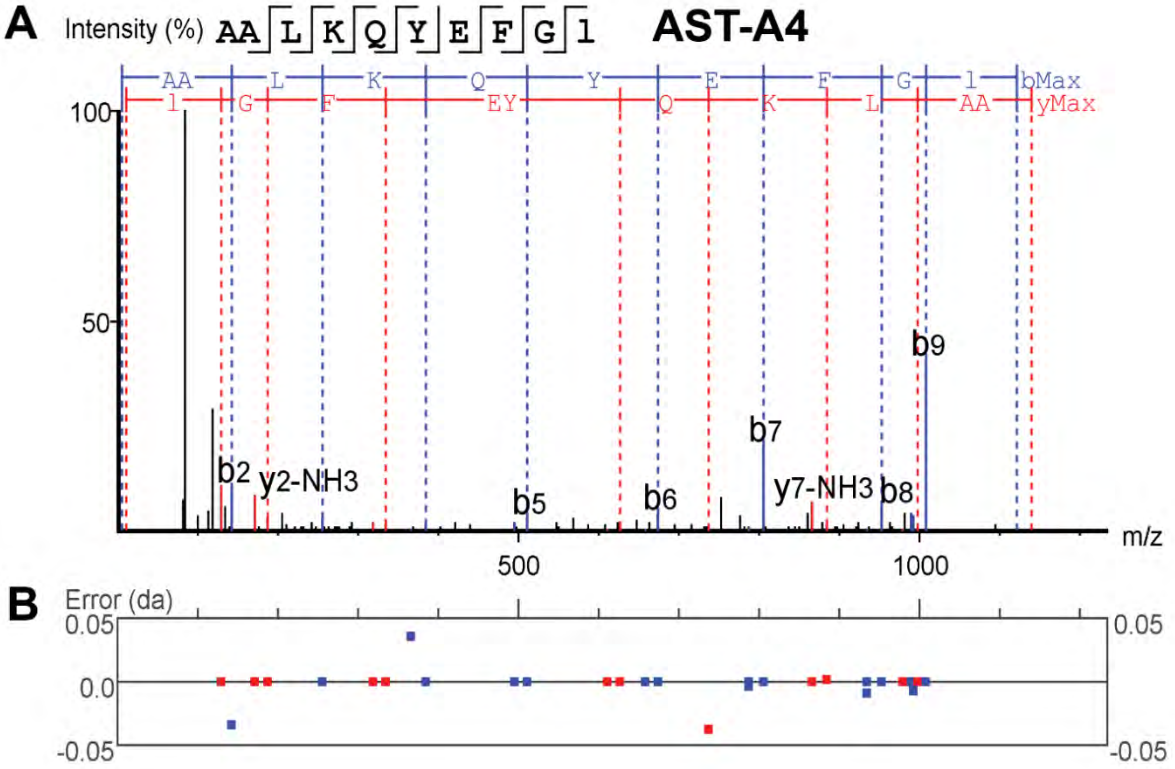

C

Ion Table

#	b	b-H2O	b-NH3	b (2+)	Seq	y	y-H2O	y-NH3	y (2+)	#
1	72.04	54.03	55.02	36.52	A					10
2	143.12	125.07	126.06	72.04	A	1067.59	1049.58	1050.56	534.29	9
3	256.17	238.16	239.14	128.58	L	996.55	978.54	979.52	498.78	8
4	384.26	366.25	367.20	192.63	K	883.46	865.46	866.44	442.23	7
5	512.32	494.31	495.29	256.66	Q	755.37	737.36	738.38	378.19	6
6	675.38	657.37	658.36	338.19	Y	627.31	609.30	610.29	314.16	5
7	804.42	786.41	787.40	402.71	E	464.25	446.24	447.22	232.63	4
8	951.49	933.48	934.48	476.25	F	335.21	317.20	318.18	168.10	3
9	1008.51	990.50	991.50	504.76	G	188.14	170.13	171.11	94.57	2
10					L(-.98)	131.12	113.11	114.09	66.06	1

-10lgP: 62.85; Mass: 1137.6182; Length: 10; ppm: -0.6; m/z: 569.8160; z: 2; RT: 52.98 ; Scan: 10442;

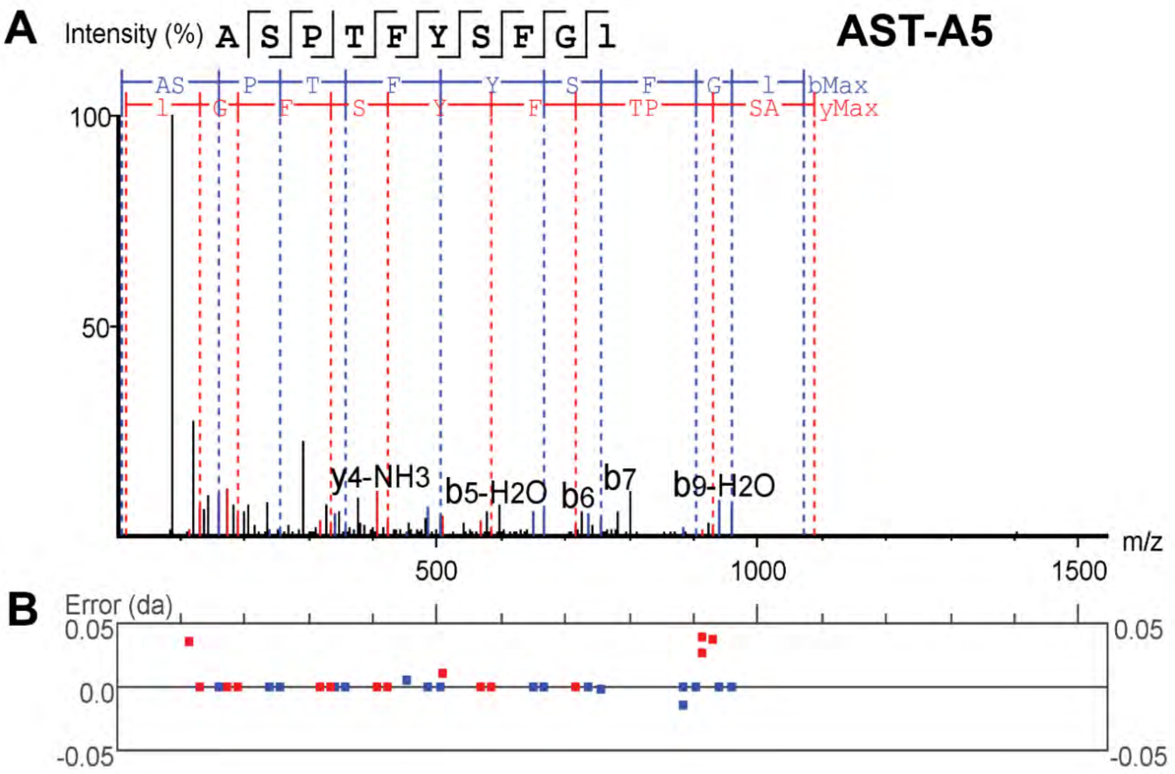

C

Ion Table

#	b	b-H2O	b-NH3	b (2+)	Seq	y	y-H2O	y-NH3	y (2+)	#
1	72.04	54.03	55.02	36.52	A					10
2	159.08	141.07	142.05	80.04	S	1017.50	999.49	1000.48	509.24	9
3	256.13	238.12	239.10	128.56	P	930.43	912.42	913.42	465.74	8
4	357.18	339.17	340.15	179.09	T	833.42	815.41	816.39	417.21	7
5	504.25	486.24	487.22	252.62	F	732.37	714.36	715.35	366.69	6
6	667.31	649.30	650.28	334.15	Y	585.30	567.29	568.28	293.15	5
7	754.34	736.33	737.31	377.67	S	422.24	404.23	405.21	211.62	4
8	901.41	883.40	884.40	451.20	F	335.21	317.20	318.18	168.10	3
9	958.43	940.42	941.40	479.72	G	188.14	170.13	171.11	94.57	2
10					L(-.98)	131.12	113.07	114.09	66.06	1

-10lgP: 48.20; Mass: 1087.5338; Length: 10; ppm: 2.7; m/z: 544.7756; z: 2; RT: 66.85 ; Scan: 13460;

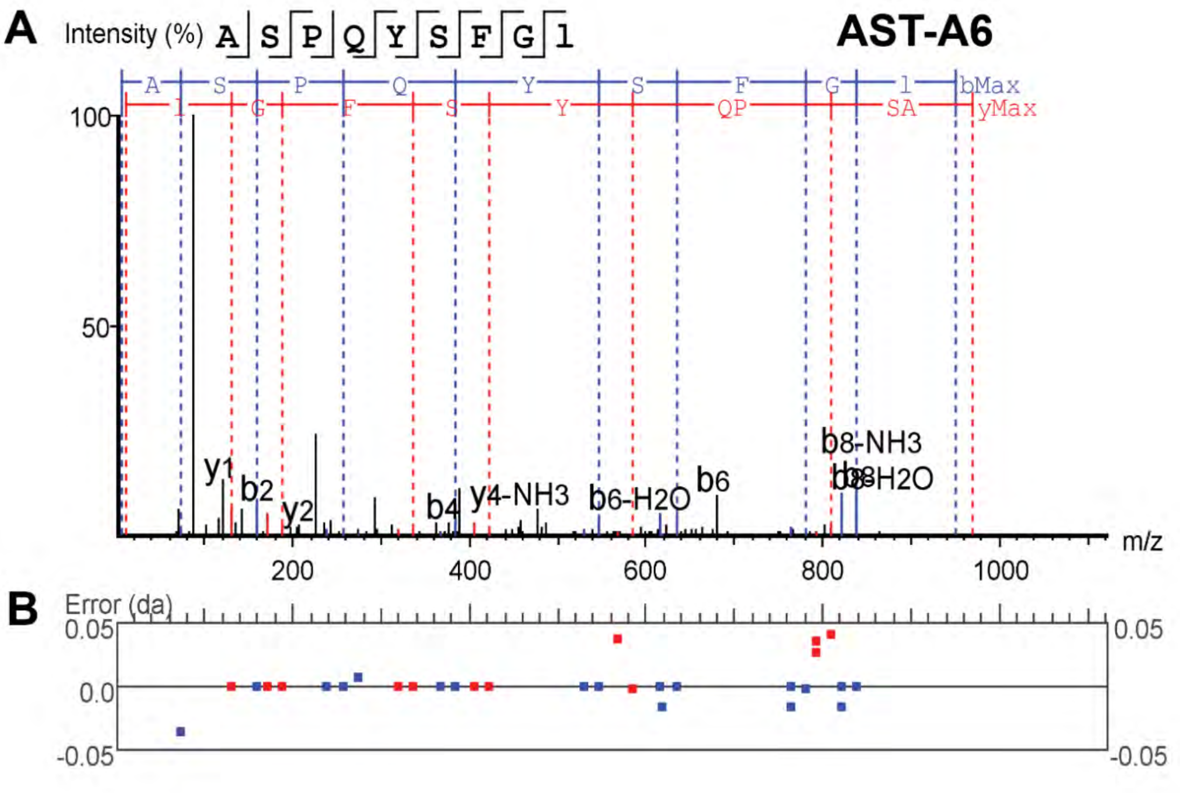

C

Ion Table

#	b	b-H2O	b-NH3	b (2+)	Seq	y	y-H2O	y-NH3	y (2+)	#
1	72.08	54.03	55.02	36.52	A					9
2	159.08	141.07	142.05	80.04	S	897.45	879.44	880.42	449.22	8
3	256.13	238.12	239.10	128.56	P	810.37	792.37	793.36	405.71	7
4	384.19	366.18	367.16	192.59	Q	713.36	695.35	696.33	357.18	6
5	547.25	529.24	530.22	274.12	Y	585.31	567.29	568.24	293.15	5
6	634.28	616.27	617.27	317.64	S	422.24	404.23	405.21	211.62	4
7	781.35	763.34	764.34	391.18	F	335.21	317.20	318.18	168.10	3
8	838.37	820.36	821.36	419.69	G	188.14	170.13	171.11	94.57	2
9					L(-.98)	131.12	113.11	114.09	66.06	1

-10lgP: 55.78; Mass: 967.4763; Length: 9; ppm: -0.1; m/z: 484.7454; z: 2; RT: 55.18 ; Scan: 10980;

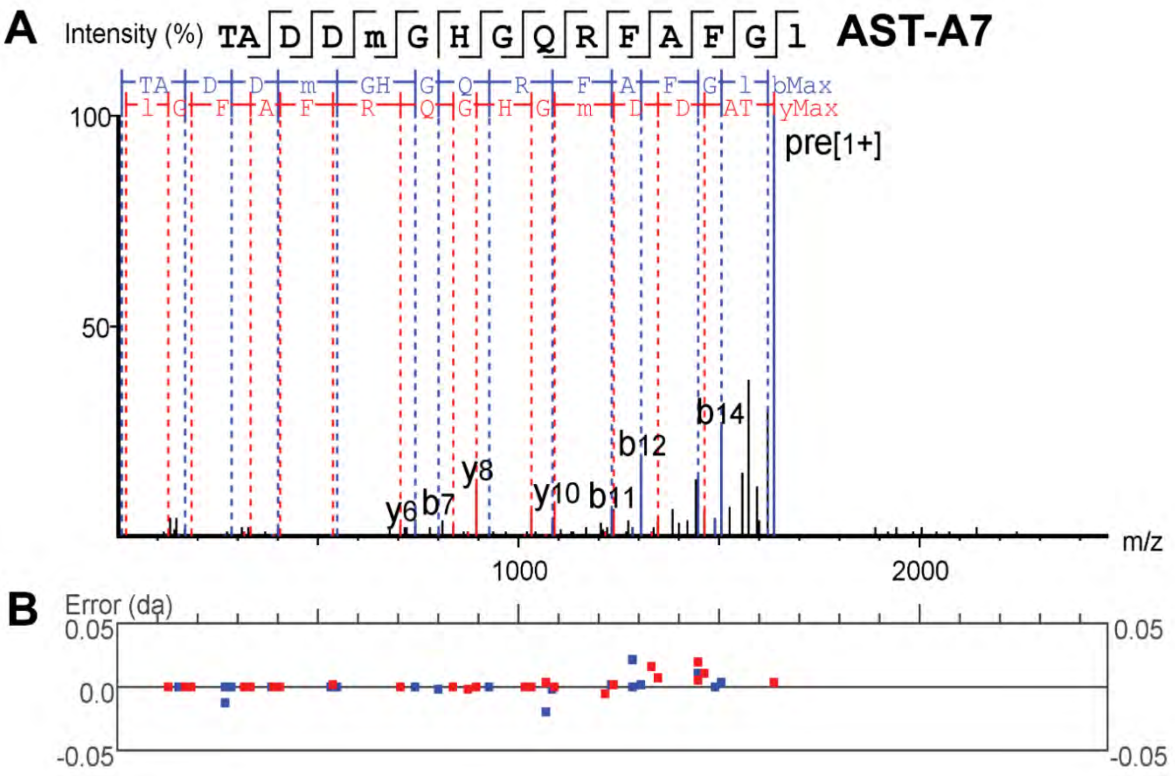

C

Ion Table

#	b	b-H2O	b-NH3	b (2+)	Seq	y	y-H2O	y-NH3	y (2+)	#
1	102.06	84.04	85.03	51.53	T					15
2	173.09	155.08	156.07	87.05	A	1536.70	1518.69	1519.67	768.85	14
3	288.12	270.11	271.11	144.56	D	1465.65	1447.63	1448.63	733.33	13
4	403.15	385.14	386.12	202.07	D	1350.63	1332.61	1333.61	675.82	12
5	550.18	532.17	533.15	275.59	M(+15.99)	1235.61	1217.60	1218.59	618.31	11
6	607.20	589.19	590.18	304.10	G	1088.58	1070.56	1071.54	544.79	10
7	744.26	726.25	727.24	372.63	H	1031.55	1013.54	1014.53	516.28	9
8	801.29	783.27	784.26	401.14	G	894.49	876.48	877.47	447.75	8
9	929.34	911.33	912.32	465.17	Q	837.47	819.46	820.45	419.24	7
10	1085.45	1067.45	1068.42	543.22	R	709.41	691.40	692.39	355.21	6
11	1232.51	1214.50	1215.48	616.76	F	553.31	535.30	536.28	277.16	5
12	1303.55	1285.54	1286.50	652.27	A	406.25	388.23	389.22	203.62	4
13	1450.60	1432.61	1433.59	725.81	F	335.21	317.20	318.18	168.10	3
14	1507.63	1489.63	1490.61	754.32	G	188.14	170.13	171.11	94.57	2
15					L(-.98)	131.12	113.11	114.09	66.06	1

-10lgP: 87.55; Mass: 1636.7416; Length: 15; ppm: 1.7; m/z: 819.3795; z: 2; RT: 50.90 ; Scan: 10007;

A

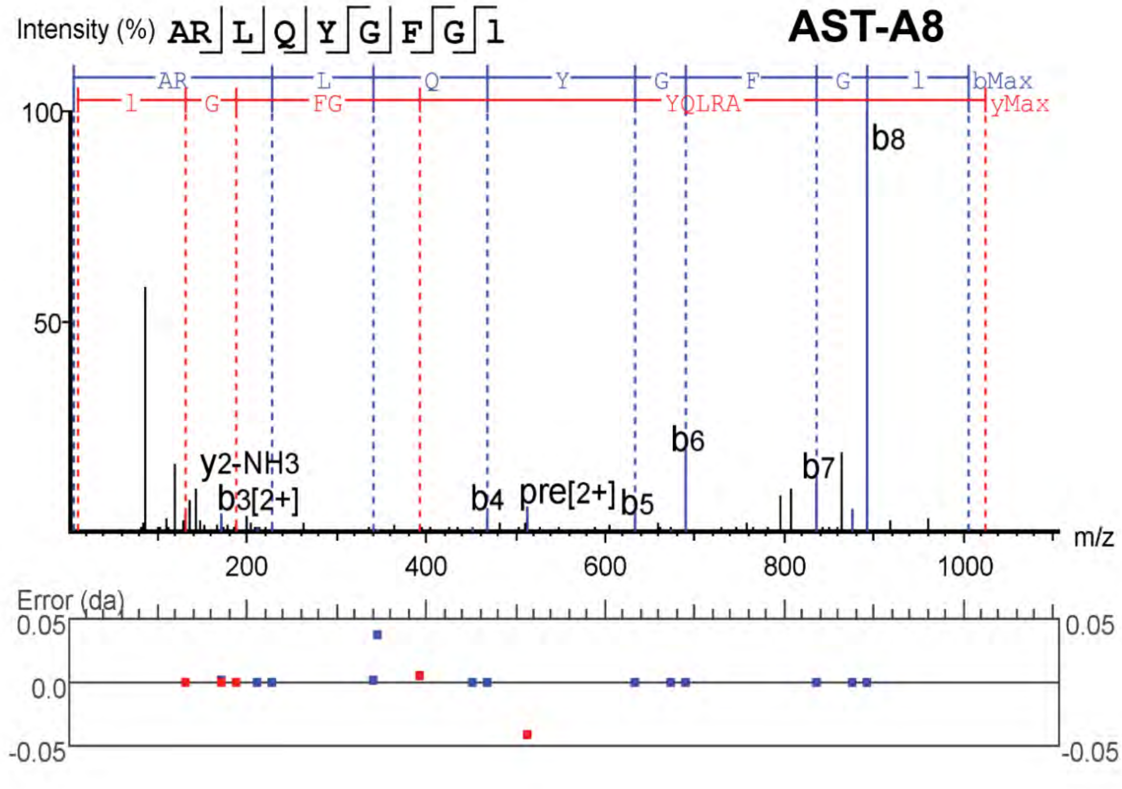

C

Ion Table

#	b	b-H2O	b-NH3	b (2+)	Seq	y	y-H2O	y-NH3	y (2+)	#
1	72.04	54.03	55.02	36.52	A					9
2	228.15	210.14	211.12	114.57	R	952.54	934.53	935.51	476.77	8
3	341.23	323.22	324.20	171.11	L	796.44	778.42	779.41	398.72	7
4	469.29	451.28	452.26	235.14	Q	683.35	665.34	666.32	342.18	6
5	632.35	614.34	615.33	316.68	Y	555.29	537.28	538.27	278.15	5
6	689.37	671.36	672.35	345.15	G	392.22	374.22	375.20	196.61	4
7	836.44	818.43	819.41	418.72	F	335.21	317.20	318.18	168.10	3
8	893.46	875.45	876.44	447.23	G	188.14	170.13	171.11	94.57	2
9					L(-.98)	131.12	113.11	114.09	66.06	1

-10lgP: 33.50; Mass: 1022.5661; Length: 9; ppm: 3.7; m/z: 512.2922; z: 2; RT: 54.17 ; Scan: 10610;

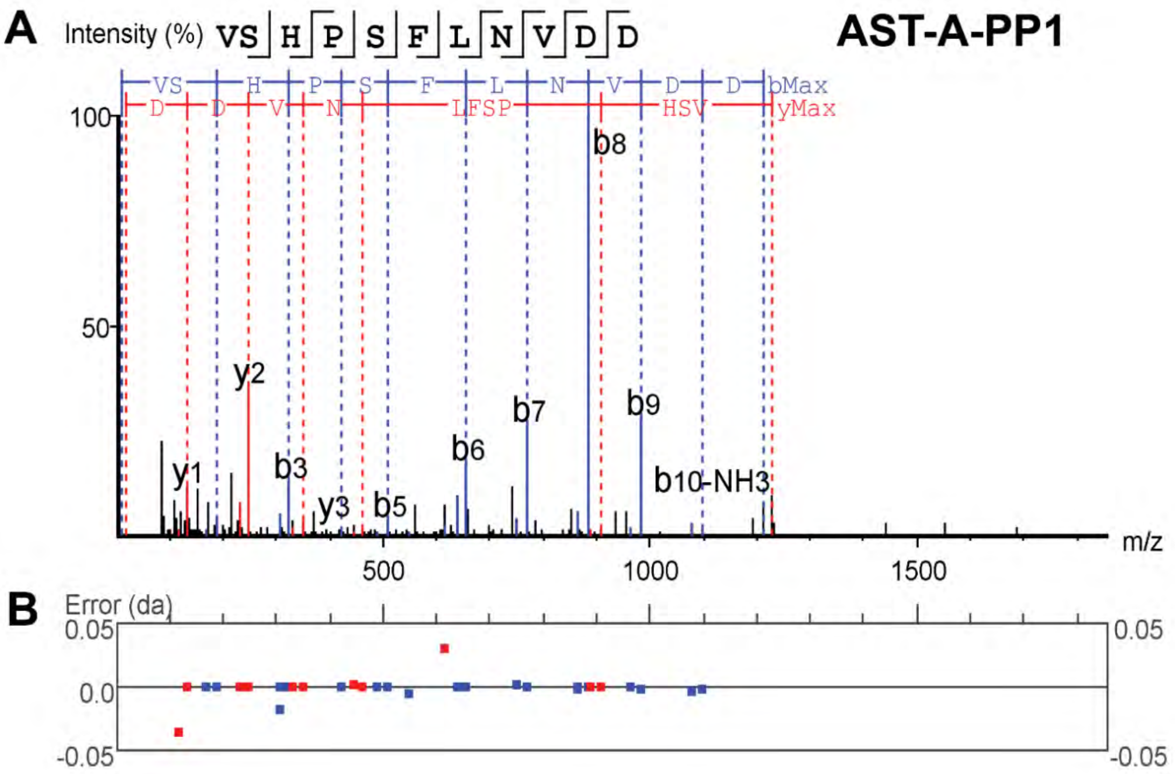

C Ion Table

#	b	b-H2O	b-NH3	b (2+)	Seq	y	y-H2O	y-NH3	y (2+)	#
1	100.08	82.07	83.05	50.54	V					11
2	187.11	169.10	170.08	94.05	S	1130.51	1112.50	1113.48	565.76	10
3	324.17	306.16	307.16	162.58	H	1043.48	1025.47	1026.45	522.24	9
4	421.22	403.21	404.19	211.11	P	906.42	888.41	889.39	453.71	8
5	508.25	490.24	491.22	254.63	S	809.37	791.36	792.34	405.18	7
6	655.32	637.31	638.29	328.16	F	722.34	704.32	705.31	361.67	6
7	768.40	750.39	751.38	384.70	L	575.27	557.26	558.24	288.13	5
8	882.45	864.44	865.42	441.72	N	462.18	444.17	445.15	231.59	4
9	981.52	963.51	964.49	491.26	V	348.14	330.13	331.11	174.57	3
10	1096.55	1078.53	1079.52	548.78	D	249.07	231.06	232.04	125.04	2
11					D	134.04	116.07	117.02	67.52	1

-10lgP: 71.46; Mass: 1228.5724; Length: 11; ppm: 2.3; m/z: 615.2949; z: 2; RT: 50.68 ; Scan: 9957;

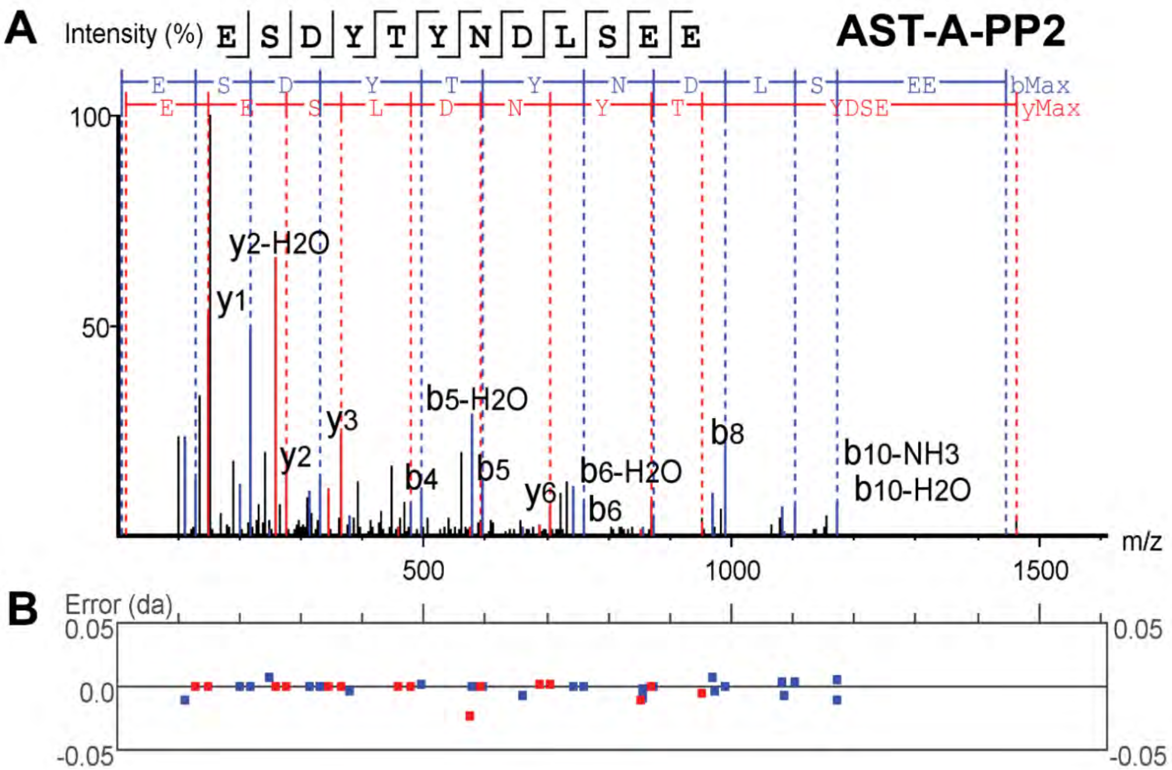

C

Ion Table

#	b	b-H2O	b-NH3	b (2+)	Seq	y	y-H2O	y-NH3	y (2+)	#
1	130.05	112.05	113.02	65.53	E					12
2	217.08	199.07	200.06	109.04	S	1335.52	1317.51	1318.50	668.26	11
3	332.11	314.10	315.08	166.55	D	1248.49	1230.48	1231.46	624.75	10
4	495.17	477.16	478.15	248.08	Y	1133.46	1115.45	1116.44	567.23	9
5	596.22	578.21	579.19	298.61	T	970.40	952.40	953.37	485.70	8
6	759.28	741.27	742.26	380.15	Y	869.35	851.35	852.33	435.18	7
7	873.33	855.32	856.31	437.16	N	706.29	688.28	689.26	353.64	6
8	988.35	970.33	971.33	494.68	D	592.25	574.24	575.24	296.62	5
9	1101.43	1083.42	1084.42	551.22	L	477.22	459.21	460.19	239.11	4
10	1188.47	1170.45	1171.45	594.73	S	364.14	346.12	347.11	182.57	3
11	1317.51	1299.50	1300.49	659.26	E	277.10	259.09	260.08	139.05	2
12					E	148.06	130.05	131.03	74.53	1

-10lgP: 66.89; Mass: 1463.5576; Length: 12; ppm: 1.9; m/z: 732.7875 ; z: 2; RT: 43.01 ; Scan: 8421;

A

B

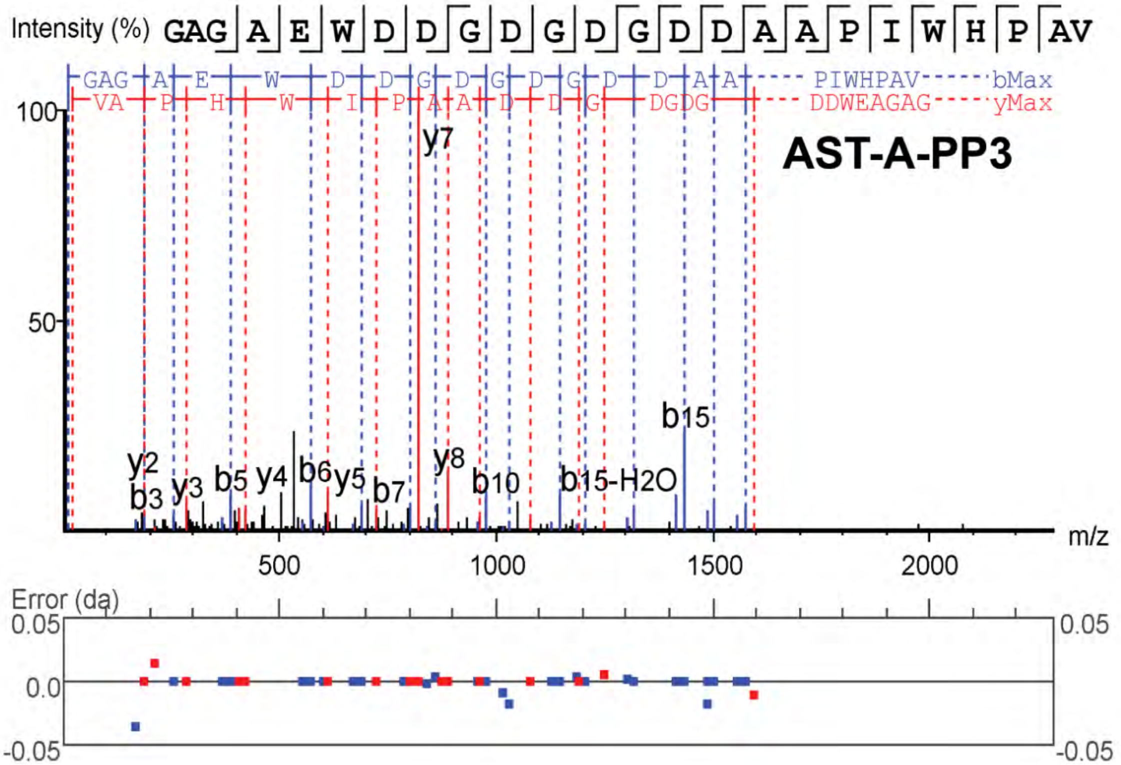

C

Ion Table

#	b	b-H2O	b-NH3	b (2+)	Seq	y	y-H2O	y-NH3	y (2+)	#
1	58.03	40.02	41.00	29.51	G					24
2	129.07	111.06	112.04	65.03	A	2336.97	2318.96	2319.94	1168.98	23
3	186.09	168.08	169.10	93.54	G	2265.93	2247.92	2248.90	1133.47	22
4	257.12	239.11	240.10	129.06	A	2208.91	2190.90	2191.88	1104.96	21
5	386.17	368.16	369.14	193.58	E	2137.87	2119.86	2120.85	1069.44	20
6	572.25	554.24	555.22	286.62	W	2008.83	1990.82	1991.80	1004.92	19
7	687.27	669.26	670.25	344.14	D	1822.75	1804.74	1805.72	911.88	18
8	802.30	784.29	785.27	401.65	D	1707.72	1689.71	1690.70	854.36	17
9	859.32	841.31	842.30	430.16	G	1592.71	1574.69	1575.67	796.85	16
10	974.35	956.34	957.32	487.67	D	1535.68	1517.67	1518.65	768.34	15
11	1031.39	1013.37	1014.34	516.19	G	1420.65	1402.64	1403.62	710.82	14
12	1146.40	1128.39	1129.37	573.70	D	1363.63	1345.62	1346.60	682.31	13
13	1203.42	1185.40	1186.39	602.21	G	1248.59	1230.59	1231.57	624.80	12
14	1318.45	1300.43	1301.42	659.72	D	1191.58	1173.57	1174.55	596.29	11
15	1433.47	1415.46	1416.45	717.24	D	1076.55	1058.54	1059.53	538.78	10
16	1504.51	1486.50	1487.50	752.76	A	961.53	943.51	944.50	481.26	9
17	1575.55	1557.54	1558.52	788.27	A	890.49	872.48	873.46	445.74	8
18	1672.60	1654.59	1655.57	836.80	P	819.45	801.44	802.42	410.23	7
19	1785.68	1767.67	1768.66	893.34	I	722.40	704.39	705.37	361.70	6
20	1971.76	1953.75	1954.74	986.38	W	609.31	591.30	592.29	305.16	5
21	2108.82	2090.81	2091.80	1054.91	H	423.24	405.22	406.21	212.10	4
22	2205.88	2187.86	2188.85	1103.44	P	286.18	268.17	269.15	143.59	3
23	2276.91	2258.90	2259.89	1138.96	A	189.12	171.11	172.10	95.06	2
24					V	118.09	100.08	101.06	59.54	1

-10lgP: 82.81; Mass: 2392.9832; Length: 24; ppm: -0.4; m/z: 798.6680 ; z: 3; RT: 60.97; Scan: 12211;

4.2 AST-B

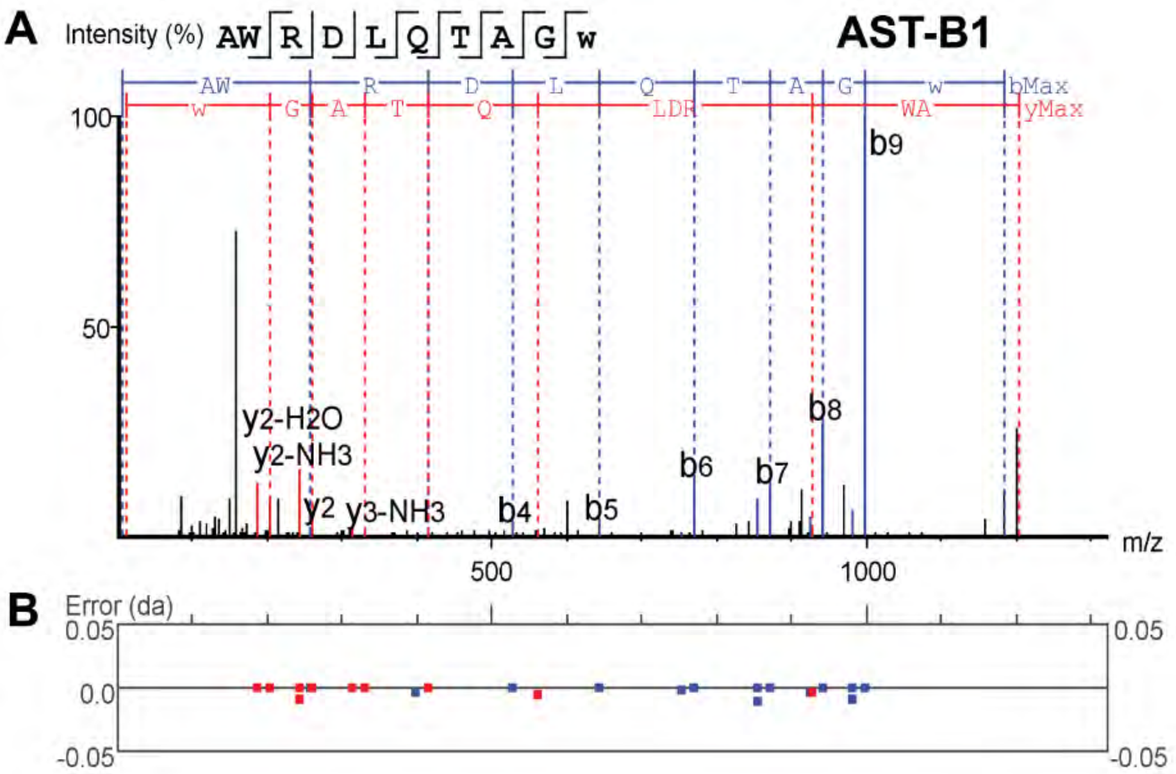

C

Ion Table

#	b	b-H2O	b-NH3	b (2+)	Seq	y	y-H2O	y-NH3	y (2+)	#
1	72.04	54.03	55.02	36.52	A					10
2	258.12	240.11	241.10	129.56	W	1131.57	1113.56	1114.54	566.28	9
3	414.23	396.21	397.20	207.61	R	945.49	927.48	928.47	473.25	8
4	529.25	511.24	512.23	265.13	D	789.39	771.38	772.36	395.19	7
5	642.34	624.33	625.31	321.67	L	674.36	656.35	657.34	337.68	6
6	770.39	752.38	753.37	385.70	Q	561.28	543.27	544.25	281.14	5
7	871.44	853.43	854.43	436.22	T	433.22	415.21	416.19	217.11	4
8	942.48	924.47	925.45	471.74	A	332.17	314.16	315.15	166.59	3
9	999.50	981.49	982.48	500.25	G	261.13	243.13	244.11	131.07	2
10					W(-.98)	204.11	186.10	187.09	102.56	1

-10lgP: 46.53; Mass: 1201.5992; Length: 10; ppm: 1.6; m/z: 601.8079 ; z: 2; RT: 52.14; Scan: 10265;

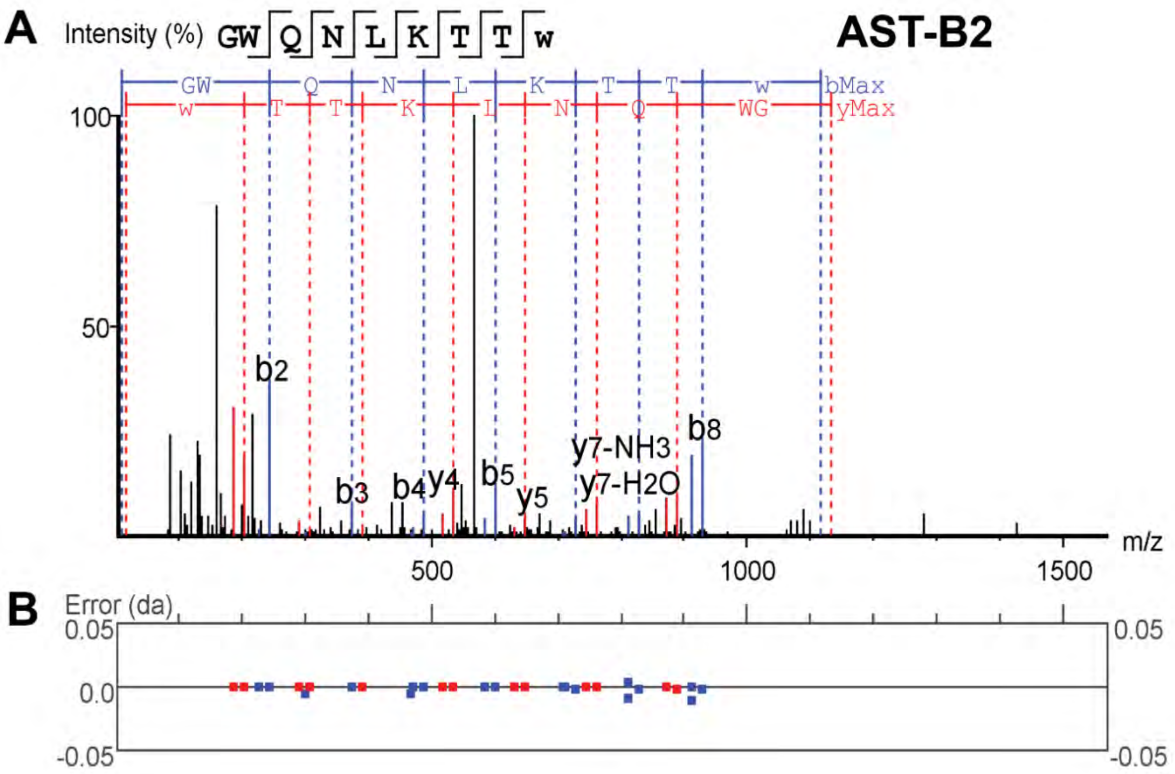

C

Ion Table

#	b	b-H2O	b-NH3	b (2+)	Seq	y	y-H2O	y-NH3	y (2+)	#
1	58.03	40.02	41.00	29.51	G					9
2	244.11	226.10	227.08	122.55	W	1075.57	1057.56	1058.54	538.28	8
3	372.17	354.16	355.14	186.58	Q	889.49	871.48	872.46	445.24	7
4	486.21	468.20	469.18	243.61	N	761.43	743.42	744.40	381.22	6
5	599.29	581.28	582.27	300.15	L	647.39	629.38	630.36	324.19	5
6	727.39	709.38	710.36	364.19	K	534.30	516.29	517.28	267.65	4
7	828.44	810.42	811.42	414.72	T	406.21	388.20	389.18	203.60	3
8	929.49	911.47	912.47	465.25	T	305.16	287.15	288.13	153.08	2
9					W(-.98)	204.11	186.10	187.09	102.56	1

-10lgP: 60.28; Mass: 1131.5825; Length: 9; ppm: 0.9; m/z: 566.7991; z: 2; RT: 51.97 ; Scan: 10321;

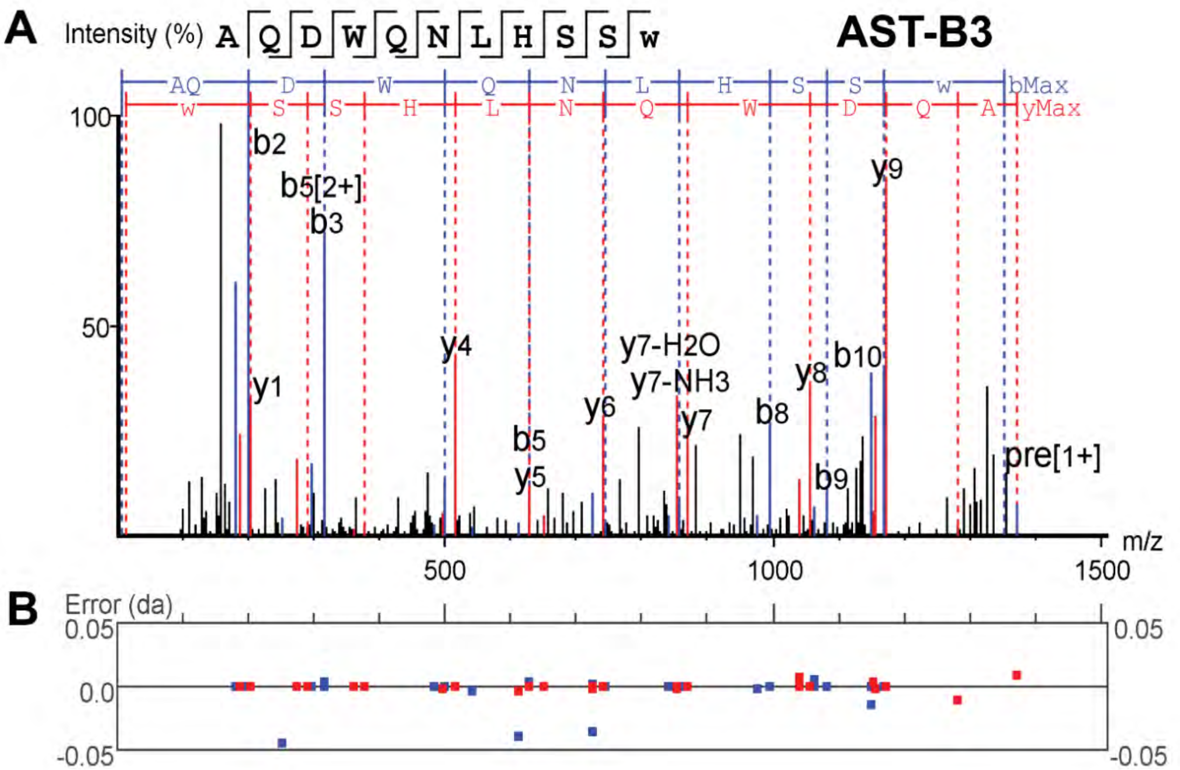

C

Ion Table

#	b	b-H2O	b-NH3	b (2+)	Seq	y	y-H2O	y-NH3	y (2+)	#
1	72.04	54.03	55.02	36.52	A					11
2	200.10	182.09	183.08	100.55	Q	1299.59	1281.58	1282.57	650.29	10
3	315.13	297.12	298.10	158.07	D	1171.53	1153.51	1154.50	586.26	9
4	501.21	483.20	484.18	251.15	W	1056.50	1038.48	1039.47	528.75	8
5	629.26	611.30	612.24	315.13	Q	870.42	852.41	853.40	435.71	7
6	743.31	725.34	726.28	372.16	N	742.36	724.35	725.34	371.68	6
7	856.39	838.38	839.37	428.70	L	628.32	610.31	611.30	314.66	5
8	993.46	975.45	976.43	497.23	H	515.24	497.23	498.21	258.12	4
9	1080.49	1062.47	1063.46	540.75	S	378.18	360.17	361.15	189.59	3
10	1167.52	1149.51	1150.51	584.26	S	291.15	273.13	274.12	146.07	2
11					W(-.98)	204.11	186.10	187.09	102.56	1

-10lgP:77.76; Mass: 1369.6163; Length: 11; ppm: -0.3 ; m/z: 685.8152; z: 2; RT: 48.79 ; Scan: 9553;

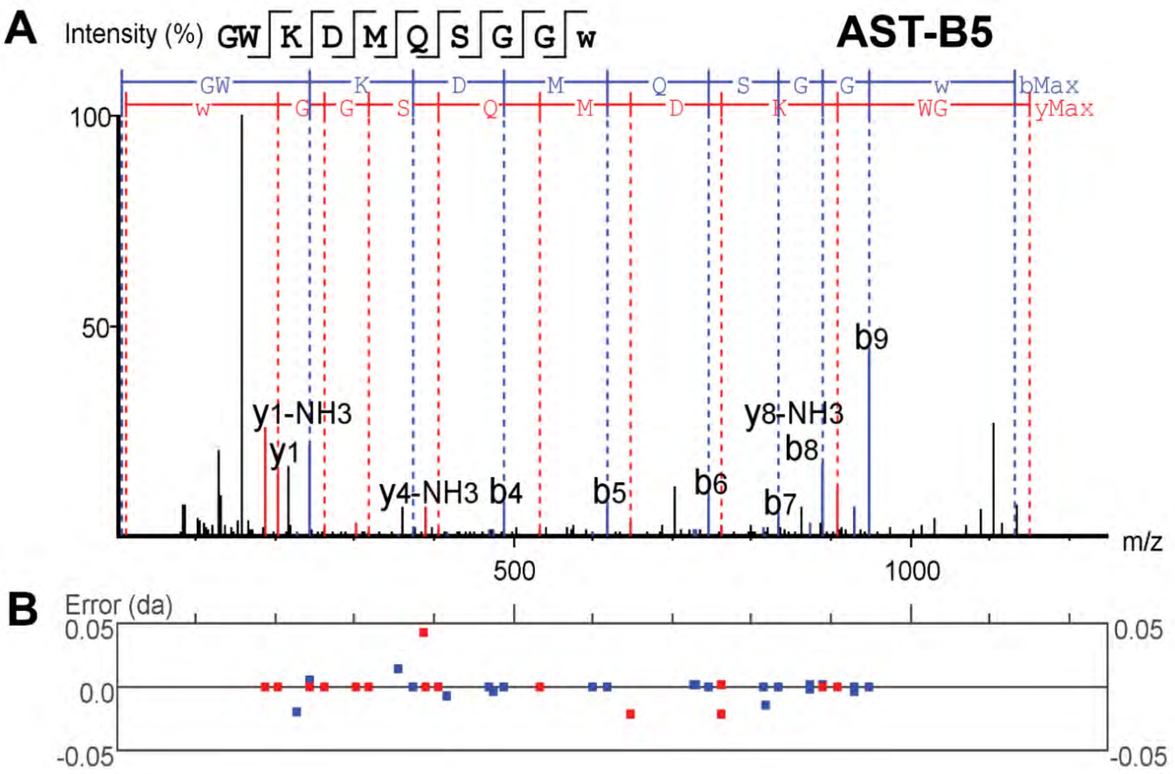

C

Ion Table

#	b	b-H2O	b-NH3	b (2+)	Seq	y	y-H2O	y-NH3	y (2+)	#
1	58.03	40.02	41.00	29.51	G					10
2	244.11	226.12	227.10	122.55	W	1093.49	1075.48	1076.46	547.24	9
3	372.20	354.19	355.16	186.60	K	907.41	889.40	890.38	454.20	8
4	487.23	469.22	470.20	244.11	D	779.31	761.33	762.28	390.16	7
5	618.27	600.26	601.24	309.64	M	664.29	646.28	647.28	332.64	6
6	746.33	728.32	729.30	373.66	Q	533.25	515.24	516.22	267.12	5
7	833.36	815.35	816.35	417.19	S	405.19	387.13	388.16	203.09	4
8	890.38	872.37	873.36	445.69	G	318.16	300.15	301.13	159.58	3
9	947.40	929.39	930.38	474.21	G	261.13	243.12	244.11	131.07	2
10					W(-.98)	204.11	186.10	187.09	102.56	1

-10lgP:57.03; Mass: 1149.5026; Length: 10; ppm: -2.4 ; m/z: 575.7572; z: 2; RT: 46.43 ; Scan: 9144;

4.3 AT

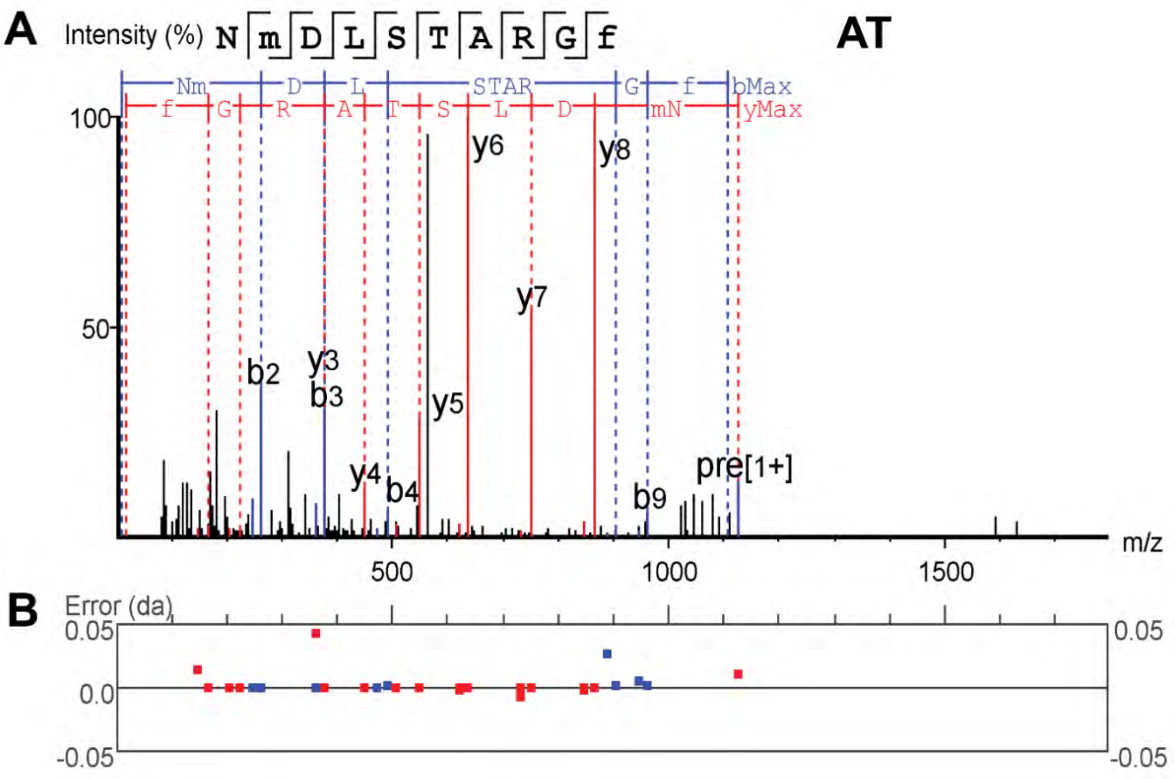

C

Ion Table

#	b	b-H2O	b-NH3	b (2+)	Seq	y	y-H2O	y-NH3	y (2+)	#
1	115.05	97.04	98.02	58.03	N					10
2	262.09	244.08	245.06	131.54	M(+15.99)	1012.49	994.48	995.46	506.75	9
3	377.11	359.10	360.09	189.06	D	865.45	847.44	848.43	433.23	8
4	490.20	472.19	473.17	245.60	L	750.43	732.42	733.41	375.71	7
5	577.23	559.22	560.20	289.11	S	637.34	619.33	620.32	319.17	6
6	678.28	660.27	661.25	339.64	T	550.31	532.30	533.28	275.65	5
7	749.31	731.30	732.29	375.16	A	449.26	431.25	432.23	225.13	4
8	905.41	887.38	888.39	453.21	R	378.22	360.21	361.15	189.61	3
9	962.43	944.43	945.40	481.72	G	222.12	204.11	205.10	111.56	2
10					F(-.98)	165.10	147.09	148.06	83.05	1

-10lgP:66.59; Mass: 1276.5282; Length: 10; ppm: -0.3 ; m/z: 639.2712 ; z: 2; RT: 38.90 ; Scan:7466;

4.4 CAPA

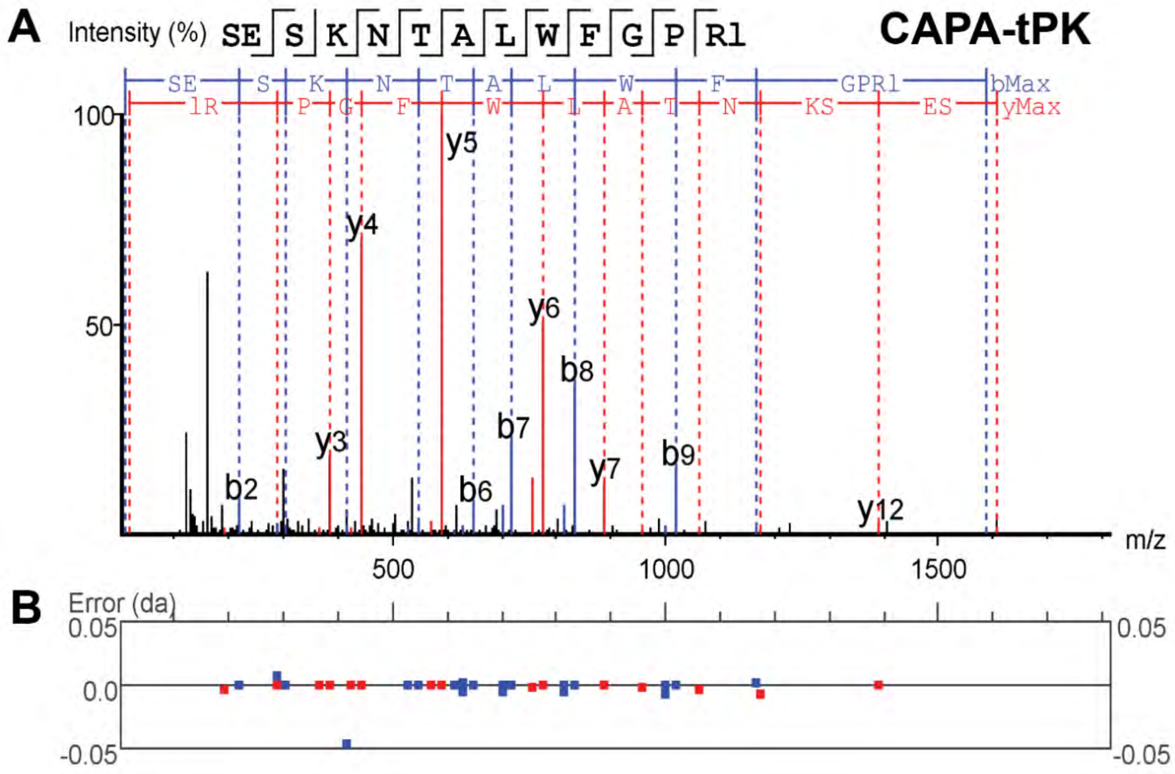

C

Ion Table

#	b	b-H2O	b-NH3	b (2+)	Seq	y	y-H2O	y-NH3	y (2+)	#
1	88.04	70.03	71.01	44.52	S					14
2	217.08	199.07	200.06	109.04	E	1517.82	1499.81	1500.80	759.41	13
3	304.11	286.10	287.08	152.56	S	1388.78	1370.77	1371.75	694.89	12
4	432.21	414.20	415.23	216.60	K	1301.75	1283.74	1284.72	651.37	11
5	546.25	528.24	529.23	273.63	N	1173.66	1155.64	1156.63	587.33	10
6	647.30	629.29	630.28	324.15	T	1059.61	1041.60	1042.58	530.30	9
7	718.34	700.33	701.32	359.67	A	958.56	940.55	941.54	479.78	8
8	831.42	813.41	814.40	416.21	L	887.53	869.51	870.50	444.26	7
9	1017.50	999.49	1000.48	509.25	W	774.44	756.43	757.42	387.72	6
10	1164.57	1146.56	1147.54	582.78	F	588.36	570.35	571.33	294.68	5
11	1221.59	1203.58	1204.56	611.30	G	441.29	423.28	424.27	221.15	4
12	1318.64	1300.63	1301.62	659.82	P	384.27	366.26	367.25	192.64	3
13	1474.74	1456.73	1457.72	737.87	R	287.22	269.21	270.19	144.11	2
14					L(-.98)	131.12	113.11	114.09	66.06	1

-10lgP: 62.93; Mass: 1603.8470; Length: 14; ppm: 1.1; m/z: 535.6235; z: 3; RT: 57.04; Scan:11386

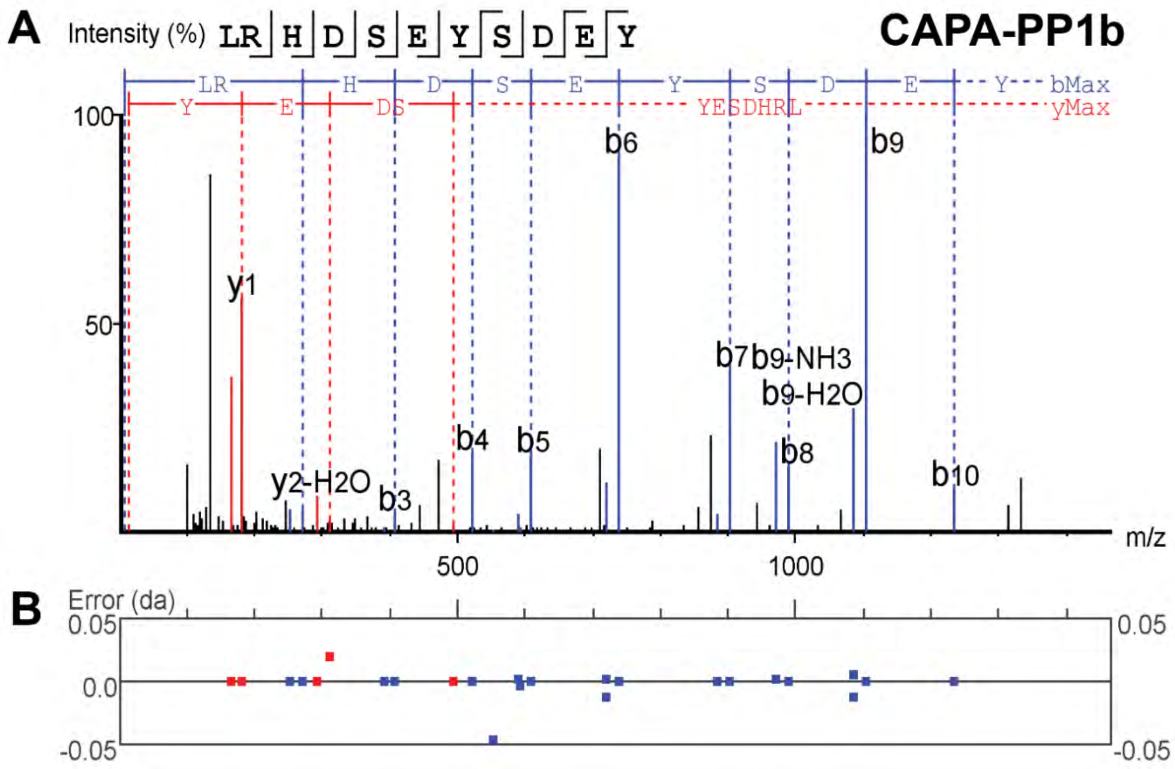

C

Ion Table

#	b	b-H2O	b-NH3	b (2+)	Seq	y	y-H2O	y-NH3	y (2+)	#
1	114.09	96.08	97.06	57.55	L					11
2	270.19	252.18	253.17	135.60	R	1300.51	1282.50	1283.48	650.75	10
3	407.25	389.24	390.22	204.13	H	1144.41	1126.40	1127.38	572.70	9
4	522.28	504.27	505.25	261.64	D	1007.35	989.34	990.32	504.17	8
5	609.31	591.30	592.29	305.16	S	892.32	874.31	875.29	446.66	7
6	738.35	720.34	721.34	369.68	E	805.29	787.28	788.26	403.14	6
7	901.42	883.41	884.39	451.21	Y	676.25	658.24	659.22	338.62	5
8	988.45	970.44	971.42	494.72	S	513.18	495.17	496.16	257.09	4
9	1103.48	1085.46	1086.46	552.29	D	426.15	408.14	409.12	213.58	3
10	1232.52	1214.51	1215.49	616.76	E	311.10	293.11	294.10	156.06	2
11					Y	182.08	164.07	165.05	91.54	1

-10lgP: 60.30; Mass: 1412.5844; Length: 11; ppm: -0.2; m/z: 707.2993; z: 2; RT: 30.45; Scan:5707

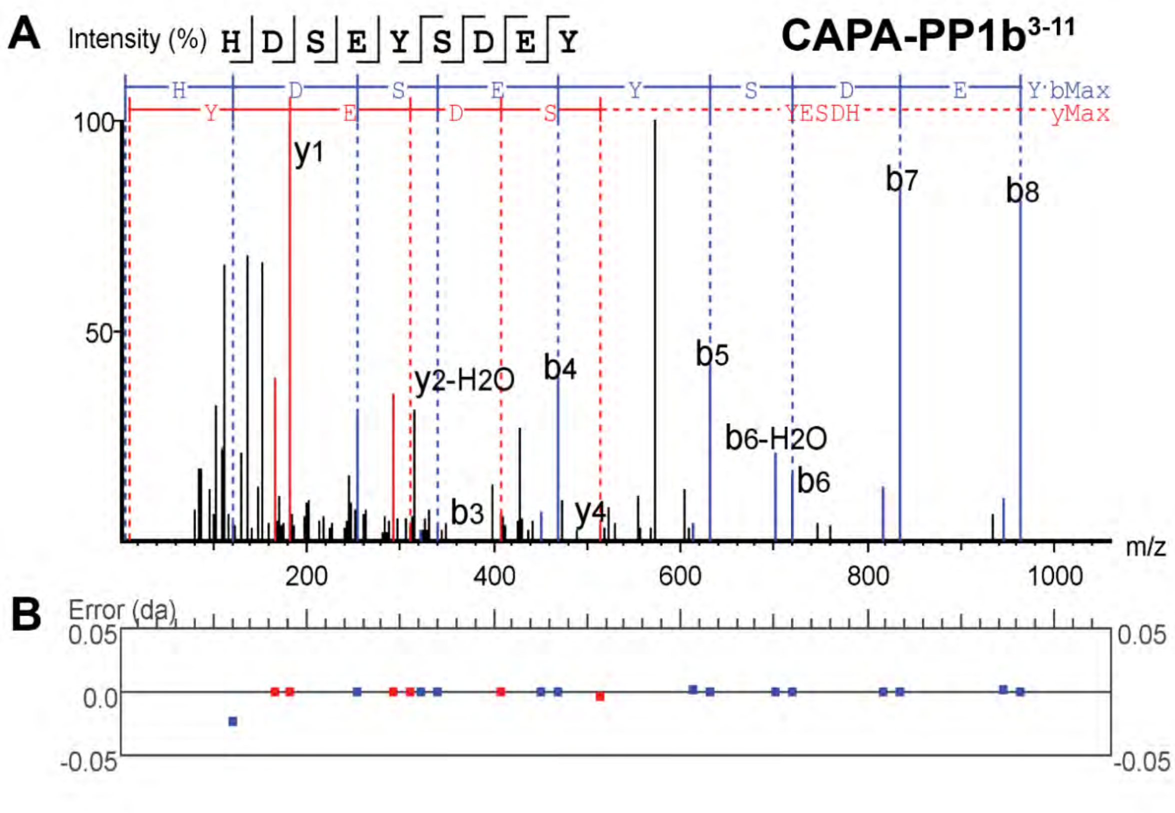

C

Ion Table

#	b	b-H2O	b-NH3	b (2+)	Seq	y	y-H2O	y-NH3	y (2+)	#
1	138.07	120.08	121.04	69.53	H					9
2	253.09	235.08	236.07	127.05	D	1007.35	989.34	990.32	504.17	8
3	340.13	322.11	323.10	170.56	S	892.32	874.31	875.29	446.66	7
4	469.17	451.16	452.14	235.08	E	805.29	787.28	788.26	403.14	6
5	632.23	614.22	615.20	316.62	Y	676.25	658.24	659.22	338.62	5
6	719.26	701.25	702.24	360.13	S	513.19	495.17	496.16	257.09	4
7	834.29	816.28	817.26	417.65	D	426.15	408.14	409.12	213.58	3
8	963.33	945.32	946.31	482.17	E	311.12	293.11	294.10	156.06	2
9					Y	182.08	164.07	165.05	91.54	1

-10lgP: 46.39; Mass: 1143.3992; Length: 9; ppm: 1; m/z: 572.7075; z: 2; RT: 30.15; Scan:5704

A

B

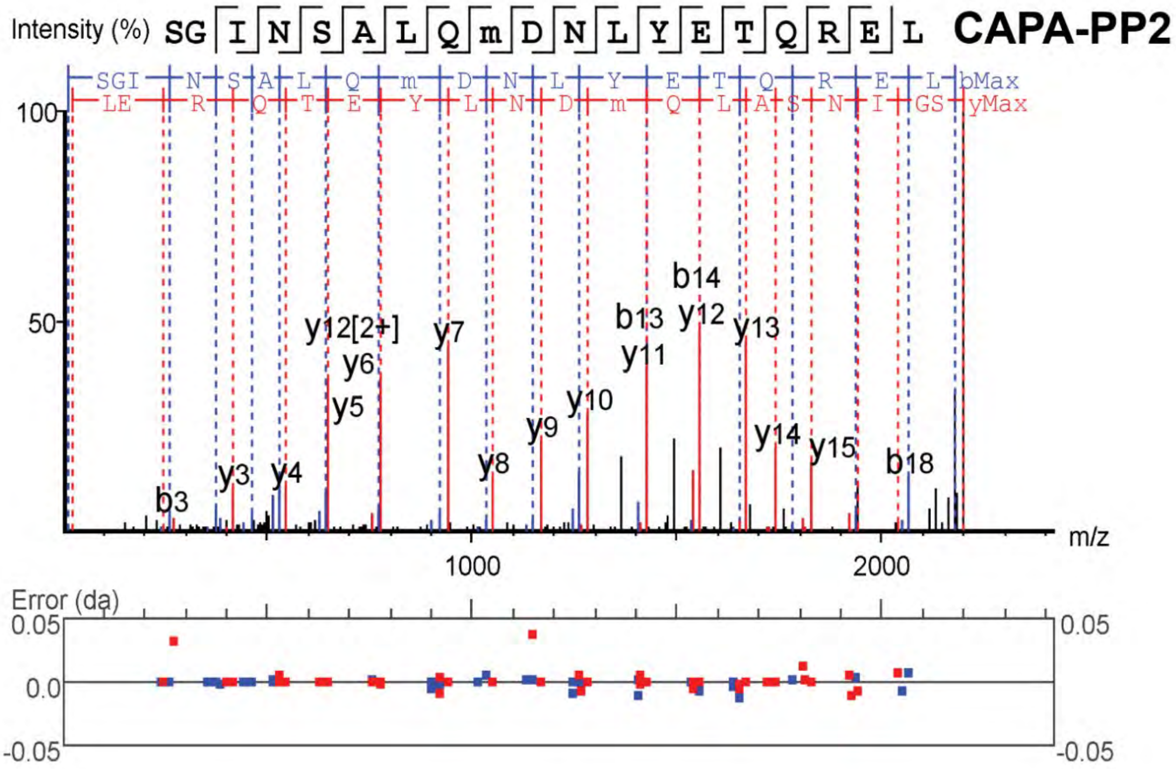

C

Ion Table

#	b	b-H2O	b-NH3	b (2+)	Seq	y	y-H2O	y-NH3	y (2+)	#
1	88.04	70.03	71.01	44.52	S					19
2	145.06	127.05	128.03	73.03	G	2111.01	2093.00	2093.98	1056.00	18
3	258.14	240.13	241.12	129.57	I	2053.99	2035.98	2036.95	1027.49	17
4	372.19	354.18	355.16	186.59	N	1940.91	1922.89	1923.89	970.95	16
5	459.22	441.21	442.19	230.11	S	1826.86	1808.83	1809.83	913.93	15
6	530.26	512.25	513.23	265.63	A	1739.83	1721.82	1722.80	870.41	14
7	643.34	625.33	626.31	322.17	L	1668.79	1650.78	1651.77	834.89	13
8	771.40	753.39	754.37	386.20	Q	1555.71	1537.70	1538.69	778.35	12
9	918.44	900.43	901.42	459.72	M(+15.99)	1427.65	1409.64	1410.61	714.32	11
10	1033.46	1015.45	1016.44	517.23	D	1280.61	1262.59	1263.59	640.81	10
11	1147.50	1129.49	1130.48	574.25	N	1165.59	1147.57	1148.52	583.29	9
12	1260.59	1242.58	1243.57	630.79	L	1051.54	1033.53	1034.51	526.27	8
13	1423.65	1405.64	1406.64	712.33	Y	938.46	920.44	921.44	469.73	7
14	1552.70	1534.68	1535.67	776.85	E	775.40	757.38	758.37	388.20	6
15	1653.76	1635.73	1636.72	827.37	T	646.35	628.34	629.32	323.68	5
16	1781.80	1763.79	1764.77	891.40	Q	545.30	527.29	528.28	273.12	4
17	1937.90	1919.89	1920.87	969.45	R	417.25	399.24	400.22	209.12	3
18	2066.94	2048.93	2049.93	1033.97	E	261.14	243.13	244.12	131.07	2
19					L	132.10	114.09	115.07	66.55	1

-10lgP: 85.34; Mass: 2197.0320; Length: 19; ppm: 2.8; m/z: 1099.5264; z: 2; RT: 55.29; Scan: 10853

4.5 CCAP

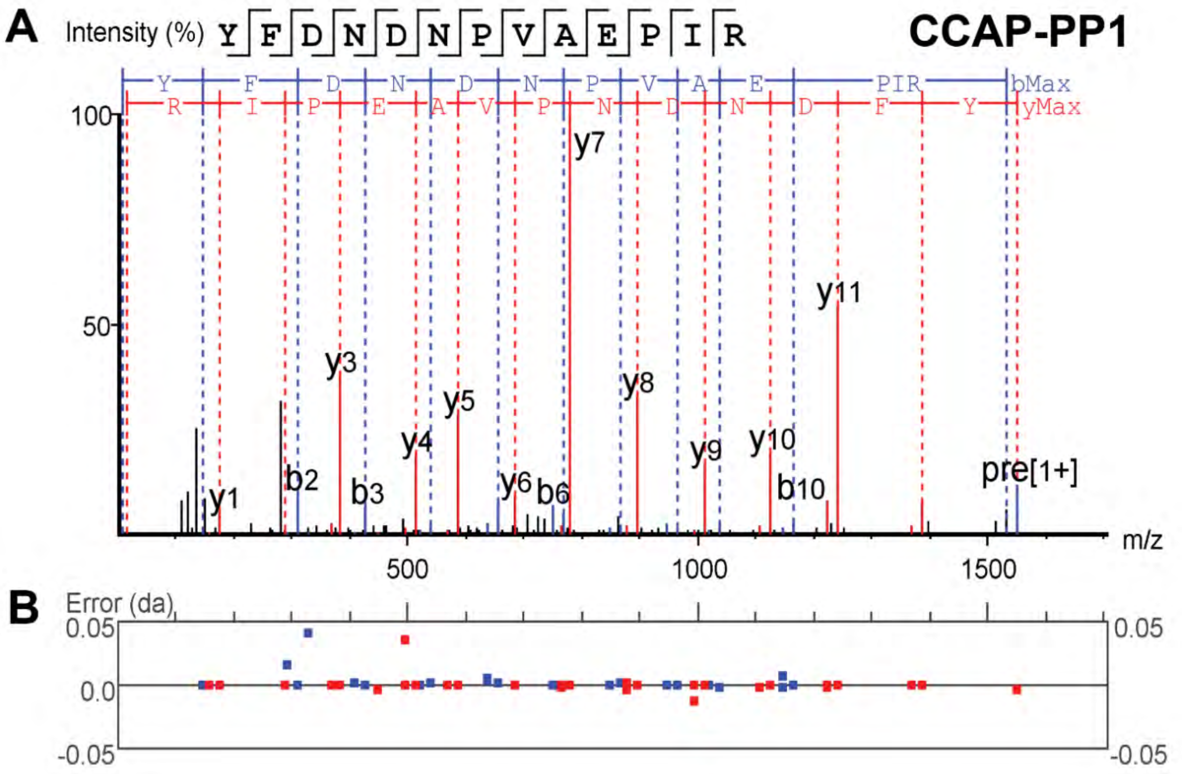

C

Ion Table

#	b	b-H2O	b-NH3	b (2+)	Seq	y	y-H2O	y-NH3	y (2+)	#
1	164.07	146.06	147.04	82.54	Y					13
2	311.14	293.11	294.11	156.07	F	1386.66	1368.65	1369.64	693.83	12
3	426.17	408.15	409.14	213.58	D	1239.60	1221.59	1222.57	620.30	11
4	540.21	522.20	523.18	270.60	N	1124.57	1106.56	1107.55	562.78	10
5	655.23	637.22	638.20	328.08	D	1010.53	992.52	993.51	505.76	9
6	769.28	751.27	752.25	385.14	N	895.50	877.49	878.48	448.25	8
7	866.33	848.32	849.31	433.67	P	781.46	763.45	764.43	391.23	7
8	965.40	947.39	948.37	483.20	V	684.40	666.39	667.38	342.70	6
9	1036.44	1018.43	1019.41	518.72	A	585.34	567.33	568.31	293.17	5
10	1165.48	1147.46	1148.46	583.24	E	514.30	496.29	497.24	257.65	4
11	1262.53	1244.52	1245.51	631.77	P	385.26	367.24	368.23	193.13	3
12	1375.62	1357.61	1358.59	688.31	I	288.20	270.19	271.18	144.60	2
13					R	175.12	157.11	158.09	88.06	1

-10lgP: 81.57; Mass: 1548.7208; Length: 13; ppm: -0.9; m/z: 775.3670; z: 2; RT: 48.80; Scan: 9562

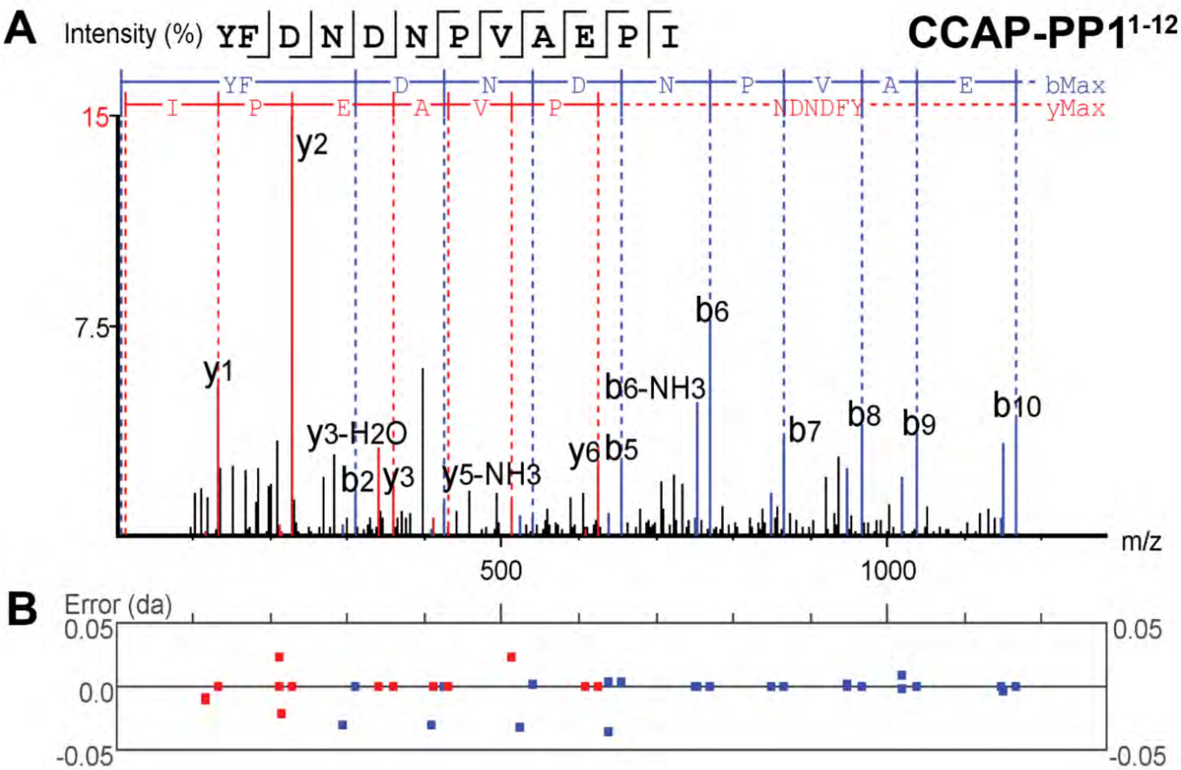

C

Ion Table

#	b	b-H2O	b-NH3	b (2+)	Seq	y	y-H2O	y-NH3	y (2+)	#
1	164.07	146.06	147.04	82.54	Y					12
2	311.14	293.16	294.14	156.07	F	1230.56	1212.55	1213.54	615.78	11
3	426.17	408.16	409.17	213.58	D	1083.50	1065.48	1066.47	542.25	10
4	540.21	522.20	523.22	270.60	N	968.47	950.46	951.44	484.73	9
5	655.23	637.22	638.25	328.12	D	854.43	836.41	837.40	427.71	8
6	769.28	751.27	752.25	385.14	N	739.40	721.39	722.37	370.20	7
7	866.33	848.32	849.31	433.67	P	625.36	607.35	608.33	313.18	6
8	965.40	947.39	948.37	483.20	V	528.30	510.29	511.25	264.65	5
9	1036.44	1018.42	1019.41	518.72	A	429.24	411.22	412.21	215.14	4
10	1165.48	1147.47	1148.46	583.24	E	358.20	340.19	341.17	179.60	3
11	1262.53	1244.52	1245.51	631.77	P	229.15	211.14	212.10	115.09	2
12					I	132.10	114.09	115.09	66.55	1

-10lgP: 63.74; Mass: 1392.6198; Length: 12; ppm: 1.1; m/z: 697.3179; z: 2; RT: 56.88; Scan: 11347

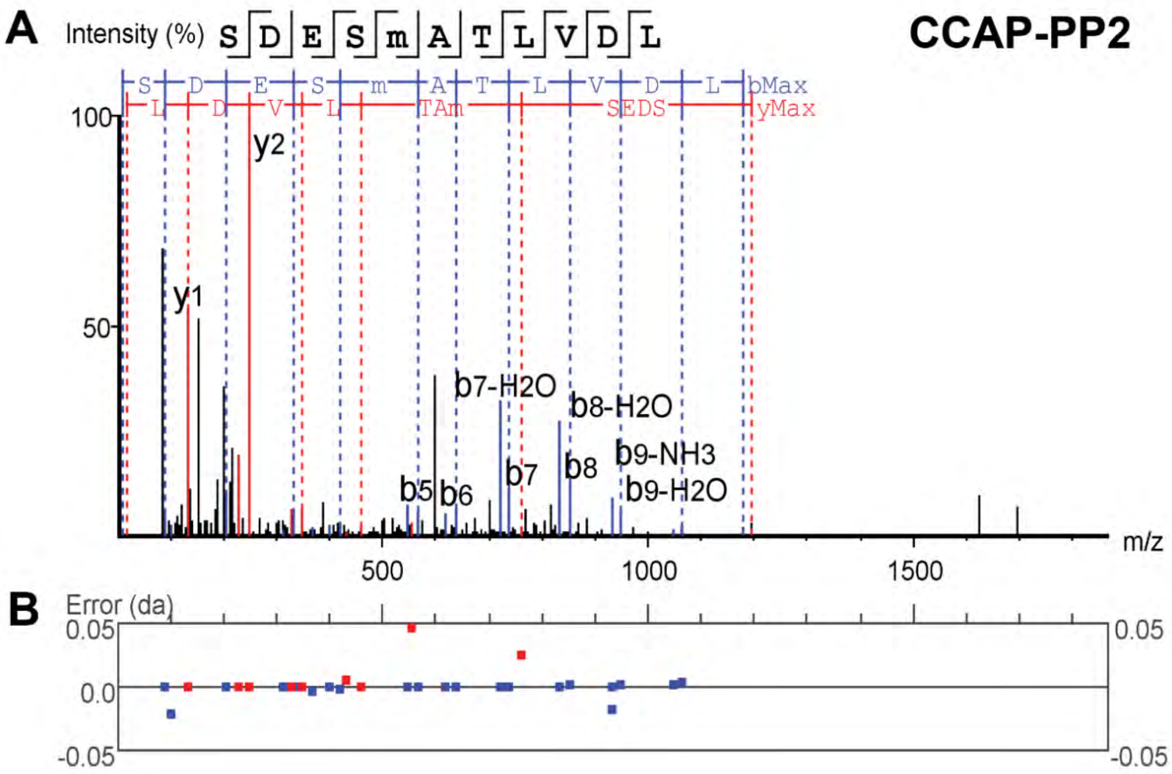

C

Ion Table

#	b	b-H2O	b-NH3	b (2+)	Seq	y	y-H2O	y-NH3	y (2+)	#
1	88.04	70.03	71.01	44.52	S					11
2	203.07	185.06	186.04	102.06	D	1109.50	1091.49	1092.48	555.20	10
3	332.11	314.10	315.08	166.55	E	994.48	976.47	977.45	497.74	9
4	419.14	401.13	402.11	210.07	S	865.43	847.42	848.41	433.21	8
5	566.18	548.17	549.15	283.59	M(+15.99)	778.40	760.36	761.37	389.70	7
6	637.21	619.20	620.19	319.11	A	631.37	613.36	614.34	316.18	6
7	738.26	720.25	721.23	369.63	T	560.33	542.32	543.30	280.66	5
8	851.34	833.33	834.32	426.17	L	459.28	441.27	442.25	230.14	4
9	950.41	932.41	933.41	475.71	V	346.20	328.19	329.17	173.60	3
10	1065.44	1047.43	1048.41	533.22	D	247.13	229.12	230.10	124.06	2
11					L	132.10	114.09	115.07	66.55	1

-10lgP: 55.39; Mass: 1195.5278; Length: 11; ppm: -0.1; m/z: 598.7711; z: 2; RT: 57.79; Scan: 11471

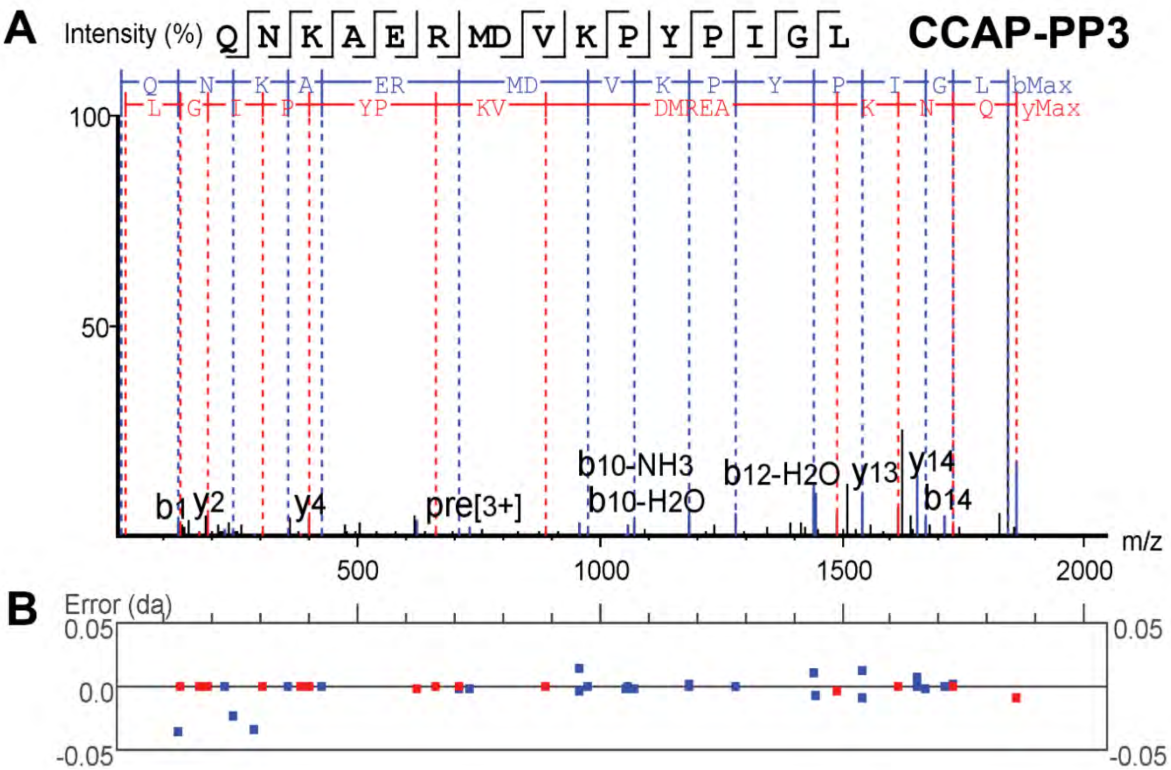

C

Ion Table

#	b	b-H2O	b-NH3	b (2+)	Seq	y	y-H2O	y-NH3	y (2+)	#
1	129.10	111.06	112.04	65.03	Q					16
2	243.13	225.10	226.08	122.05	N	1730.92	1712.92	1713.90	865.96	15
3	371.20	353.19	354.18	186.10	K	1616.88	1598.87	1599.86	808.94	14
4	442.24	424.23	425.21	221.62	A	1488.79	1470.78	1471.76	744.89	13
5	571.28	553.27	554.26	286.18	E	1417.75	1399.74	1400.72	709.38	12
6	727.39	709.38	710.36	364.19	R	1288.71	1270.70	1271.68	644.85	11
7	858.43	840.42	841.40	429.71	M	1132.61	1114.60	1115.58	566.80	10
8	973.45	955.43	956.43	487.23	D	1001.57	983.56	984.54	501.28	9
9	1072.52	1054.51	1055.49	536.76	V	886.54	868.53	869.51	443.77	8
10	1200.62	1182.60	1183.59	600.81	K	787.47	769.46	770.44	394.24	7
11	1297.67	1279.66	1280.64	649.33	P	659.37	641.37	642.35	330.19	6
12	1460.73	1442.71	1443.71	730.87	Y	562.32	544.31	545.30	281.66	5
13	1557.78	1539.76	1540.77	779.39	P	399.26	381.25	382.23	200.13	4
14	1670.87	1652.85	1653.84	835.93	I	302.21	284.20	285.18	151.60	3
15	1727.89	1709.88	1710.86	864.45	G	189.12	171.11	172.10	95.06	2
16					L	132.10	114.09	115.07	66.55	1

-10lgP: 41.57; Mass: 1857.9771; Length: 16; ppm: 1.1; m/z: 620.3336; z: 3; RT: 49.59; Scan: 9817

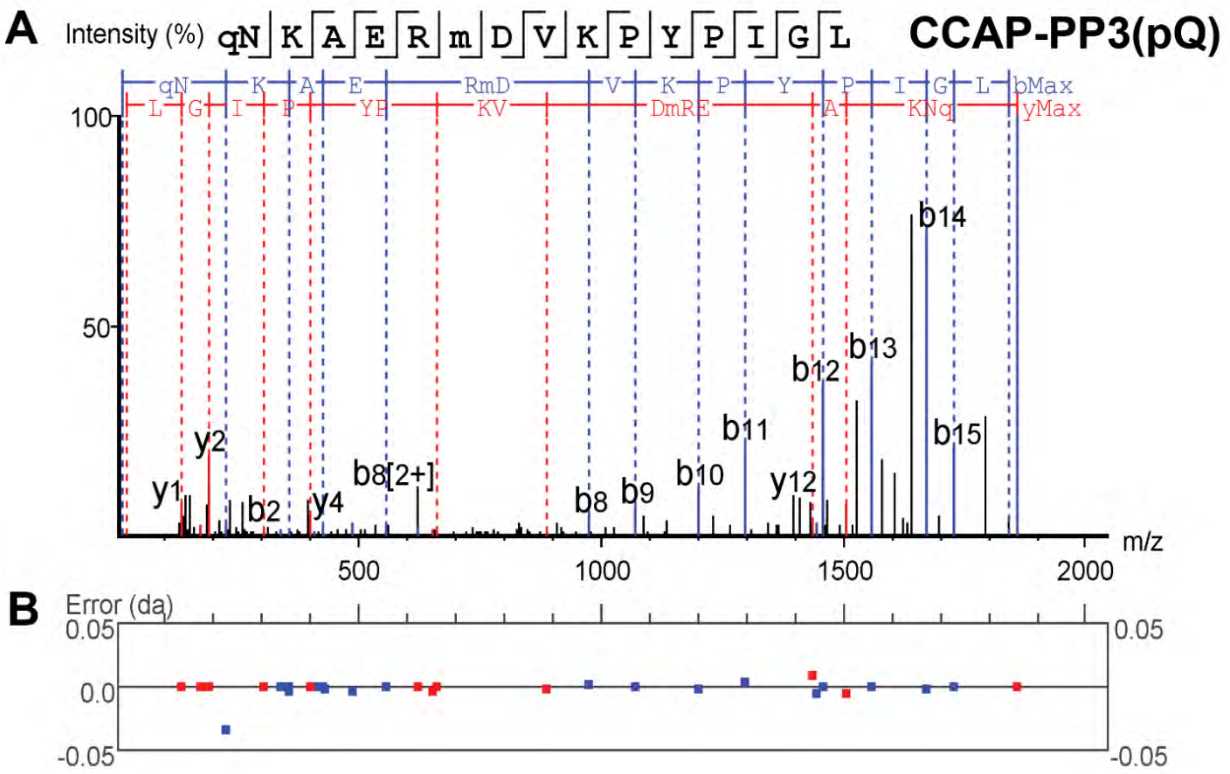

C

Ion Table

#	b	b-H2O	b-NH3	b (2+)	Seq	y	y-H2O	y-NH3	y (2+)	#
1	112.04	94.03	95.01	56.52	Q(-17.03)					16
2	226.12	208.07	209.06	113.54	N	1746.92	1728.91	1729.89	873.96	15
3	354.18	336.17	337.15	177.59	K	1632.88	1614.87	1615.85	816.94	14
4	425.21	407.20	408.19	213.11	A	1504.79	1486.77	1487.76	752.89	13
5	554.26	536.25	537.23	277.63	E	1433.74	1415.74	1416.72	717.37	12
6	710.36	692.35	693.33	355.68	R	1304.70	1286.69	1287.68	652.86	11
7	857.39	839.38	840.37	429.20	M(+15.99)	1148.60	1130.59	1131.57	574.80	10
8	972.42	954.41	955.39	486.71	D	1001.57	983.56	984.54	501.28	9
9	1071.49	1053.48	1054.46	536.24	V	886.54	868.53	869.51	443.77	8
10	1199.59	1181.57	1182.56	600.29	K	787.47	769.46	770.44	394.24	7
11	1296.63	1278.63	1279.61	648.82	P	659.37	641.37	642.35	330.19	6
12	1459.70	1441.69	1442.68	730.35	Y	562.32	544.31	545.30	281.66	5
13	1556.75	1538.74	1539.73	778.88	P	399.26	381.25	382.23	200.13	4
14	1669.84	1651.83	1652.81	835.42	I	302.21	284.20	285.18	151.60	3
15	1726.86	1708.85	1709.83	863.93	G	189.12	171.11	172.10	95.06	2
16					L	132.10	114.09	115.07	66.55	1

-10lgP: 52.04; Mass: 1856.9454; Length: 16; ppm: -0.7; m/z: 619.9886; z: 3; RT: 50.21; Scan: 9950

4.6 CNP

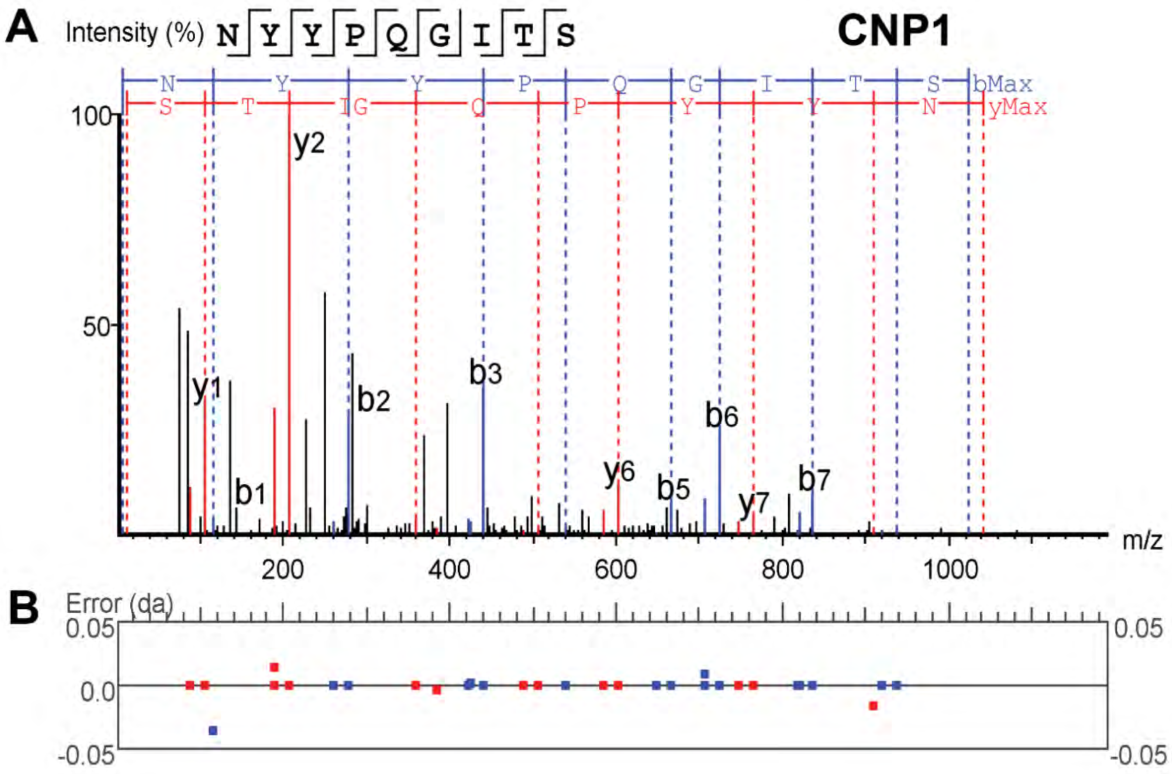

C

Ion Table

#	b	b-H2O	b-NH3	b (2+)	Seq	y	y-H2O	y-NH3	y (2+)	#
1	115.09	97.04	98.02	58.03	N					9
2	278.11	260.10	261.09	139.56	Y	928.44	910.45	911.41	464.72	8
3	441.18	423.17	424.15	221.09	Y	765.38	747.37	748.35	383.19	7
4	538.23	520.22	521.20	269.62	P	602.31	584.30	585.29	301.66	6
5	666.29	648.28	649.26	333.64	Q	505.26	487.25	488.23	253.13	5
6	723.31	705.29	706.28	362.16	G	377.20	359.19	360.18	189.09	4
7	836.39	818.38	819.37	418.70	I	320.18	302.17	303.15	160.59	3
8	937.44	919.43	920.41	469.22	T	207.10	189.09	190.07	104.05	2
9					S	106.05	88.04	89.02	53.52	1

-10lgP: 39.15; Mass: 1124.5397; Length: 9; ppm: 0.5; m/z: 375.8540; z: 2; RT: 25.99; Scan: 4775;

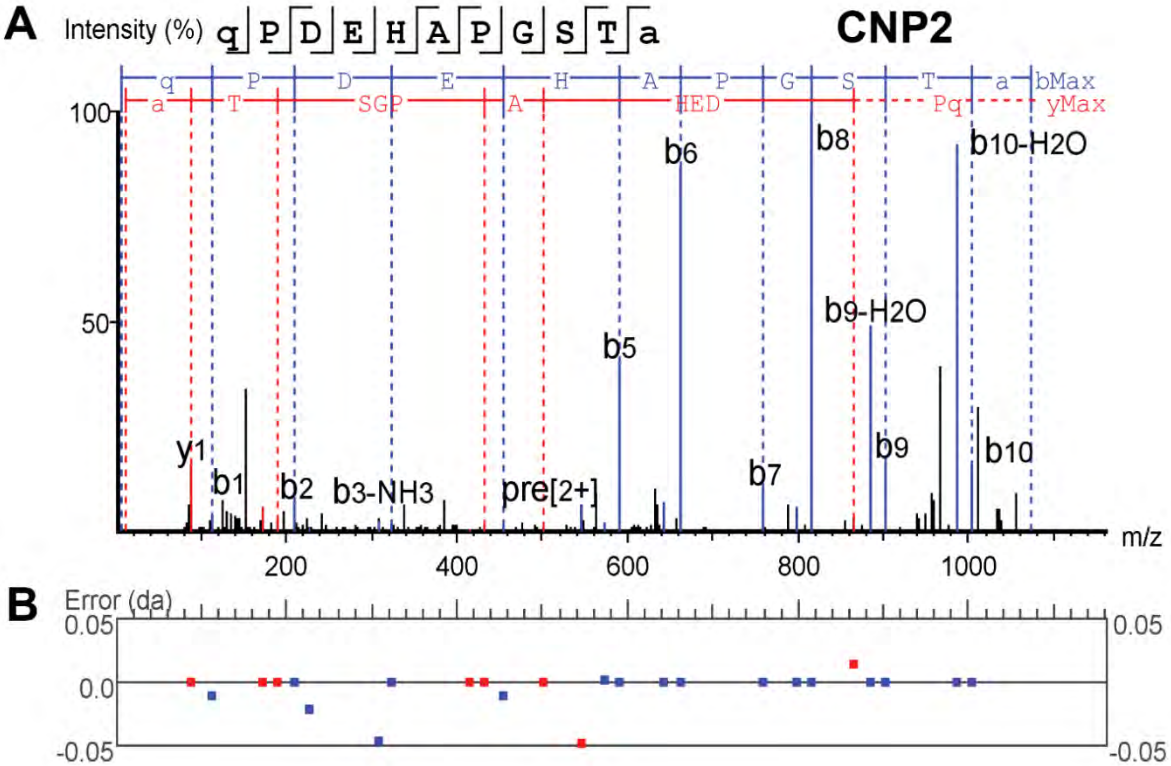

C

Ion Table

#	b	b-H2O	b-NH3	b (2+)	Seq	y	y-H2O	y-NH3	y (2+)	#
1	112.05	94.03	95.01	56.52	Q(-17.03)					11
2	209.09	191.08	192.07	105.05	P	980.44	962.43	963.42	490.72	10
3	324.12	306.11	307.14	162.56	D	883.39	865.38	866.35	442.20	9
4	453.17	435.15	436.14	227.10	E	768.36	750.35	751.34	384.68	8
5	590.22	572.21	573.19	295.61	H	639.32	621.31	622.29	320.16	7
6	661.26	643.25	644.23	331.13	A	502.26	484.25	485.23	251.63	6
7	758.31	740.30	741.28	379.66	P	431.23	413.21	414.20	216.11	5
8	815.33	797.32	798.31	408.17	G	334.17	316.16	317.15	167.59	4
9	902.36	884.35	885.34	451.68	S	277.15	259.14	260.12	139.08	3
10	1003.41	985.40	986.39	502.21	T	190.12	172.11	173.09	95.56	2
11					A(-.98)	89.07	71.06	72.04	45.04	1

-10lgP: 63.96; Mass: 1090.4679; Length: 11; ppm: 0.2; m/z: 546.2413; z: 2; RT: 29.43; Scan:5488;

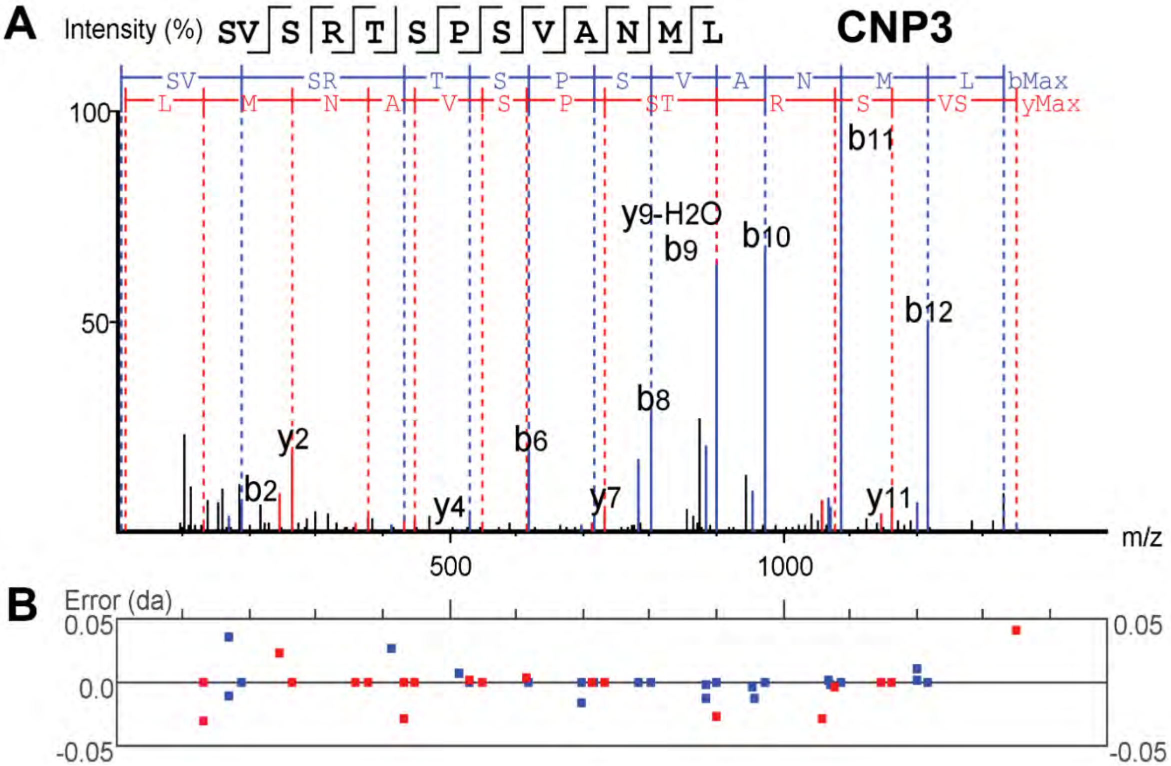

C

Ion Table

#	b	b-H2O	b-NH3	b (2+)	Seq	y	y-H2O	y-NH3	y (2+)	#
1	88.04	70.03	71.01	44.52	S					13
2	187.11	169.06	170.09	94.05	V	1261.66	1243.65	1244.63	631.33	12
3	274.14	256.13	257.11	137.57	S	1162.59	1144.58	1145.56	581.79	11
4	430.24	412.23	413.19	215.62	R	1075.56	1057.55	1058.56	538.28	10
5	531.29	513.27	514.26	266.14	T	919.46	901.47	902.43	460.23	9
6	618.32	600.31	601.29	309.66	S	818.41	800.40	801.38	409.70	8
7	715.37	697.36	698.36	358.19	P	731.38	713.37	714.35	366.19	7
8	802.41	784.39	785.38	401.70	S	634.32	616.31	617.30	317.66	6
9	901.47	883.47	884.46	451.24	V	547.29	529.28	530.26	274.15	5
10	972.51	954.51	955.50	486.76	A	448.22	430.24	431.20	224.61	4
11	1086.55	1068.54	1069.53	543.78	N	377.19	359.18	360.16	189.09	3
12	1217.59	1199.57	1200.56	609.30	M	263.14	245.13	246.09	132.10	2
13					L	132.10	114.09	115.07	66.55	1

-10lgP: 65.58; Mass: 1347.6816; Length: 13; ppm: 1.5; m/z: 674.8491; z: 2; RT: 51.16; Scan:10140;

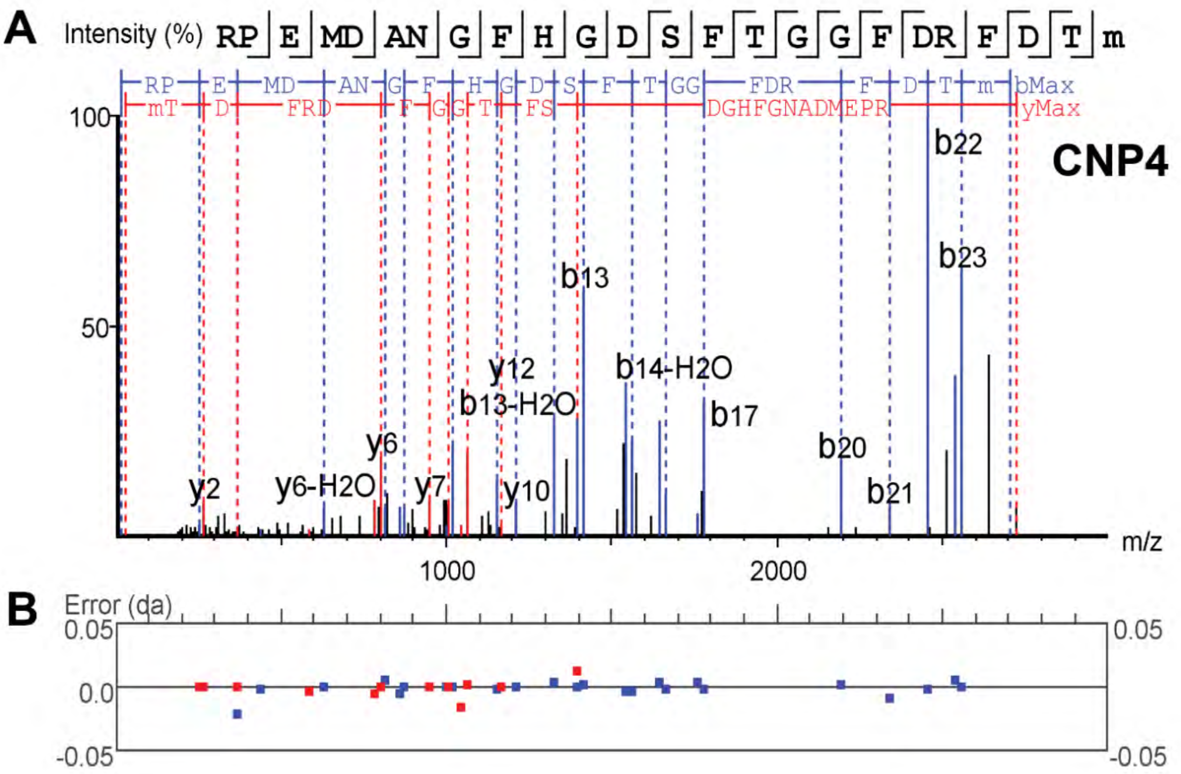

C

Ion Table

#	b	b-H2O	b-NH3	b (2+)	Seq	y	y-H2O	y-NH3	y (2+)	#
1	157.11	139.10	140.08	79.05	R					24
2	254.16	236.15	237.13	127.58	P	2567.02	2549.01	2550.00	1284.01	23
3	383.20	365.19	366.20	192.10	E	2469.97	2451.96	2452.94	1235.49	22
4	514.24	496.23	497.22	257.62	M	2340.93	2322.92	2323.90	1170.96	21
5	629.27	611.26	612.24	315.14	D	2209.89	2191.88	2192.86	1105.44	20
6	700.31	682.30	683.28	350.65	A	2094.86	2076.85	2077.83	1047.93	19
7	814.35	796.34	797.32	407.68	N	2023.82	2005.81	2006.80	1012.41	18
8	871.37	853.36	854.35	436.19	G	1909.78	1891.77	1892.75	955.39	17
9	1018.44	1000.43	1001.42	509.72	F	1852.76	1834.75	1835.73	926.88	16
10	1155.50	1137.49	1138.47	578.25	H	1705.69	1687.68	1688.66	853.35	15
11	1212.52	1194.51	1195.49	606.76	G	1568.63	1550.62	1551.61	784.82	14
12	1327.54	1309.54	1310.52	664.27	D	1511.61	1493.60	1494.58	756.31	13
13	1414.58	1396.57	1397.55	707.79	S	1396.57	1378.57	1379.56	698.79	12
14	1561.65	1543.64	1544.62	781.32	F	1309.55	1291.54	1292.52	655.28	11
15	1662.70	1644.68	1645.67	831.85	T	1162.48	1144.47	1145.46	581.75	10
16	1719.72	1701.71	1702.69	860.36	G	1061.43	1043.43	1044.42	531.22	9
17	1776.74	1758.73	1759.71	888.87	G	1004.41	986.40	987.39	502.71	8
18	1923.81	1905.80	1906.78	962.40	F	947.39	929.38	930.37	474.20	7
19	2038.84	2020.82	2021.81	1019.92	D	800.33	782.32	783.30	400.66	6
20	2194.93	2176.93	2177.91	1097.97	R	685.30	667.29	668.27	343.15	5
21	2342.01	2323.99	2324.98	1171.50	F	529.20	511.19	512.17	265.10	4
22	2457.03	2439.02	2440.00	1229.02	D	382.13	364.12	365.10	191.56	3
23	2558.08	2540.06	2541.05	1279.54	T	267.10	249.09	250.07	134.05	2
24					M(+15.99)	166.05	148.04	149.03	83.53	1

-10lgP: 87.29; Mass: 2722.1177; Length: 24; ppm: 3.8; m/z: 681.5393; z: 4; RT: 53.60; Scan:10507;

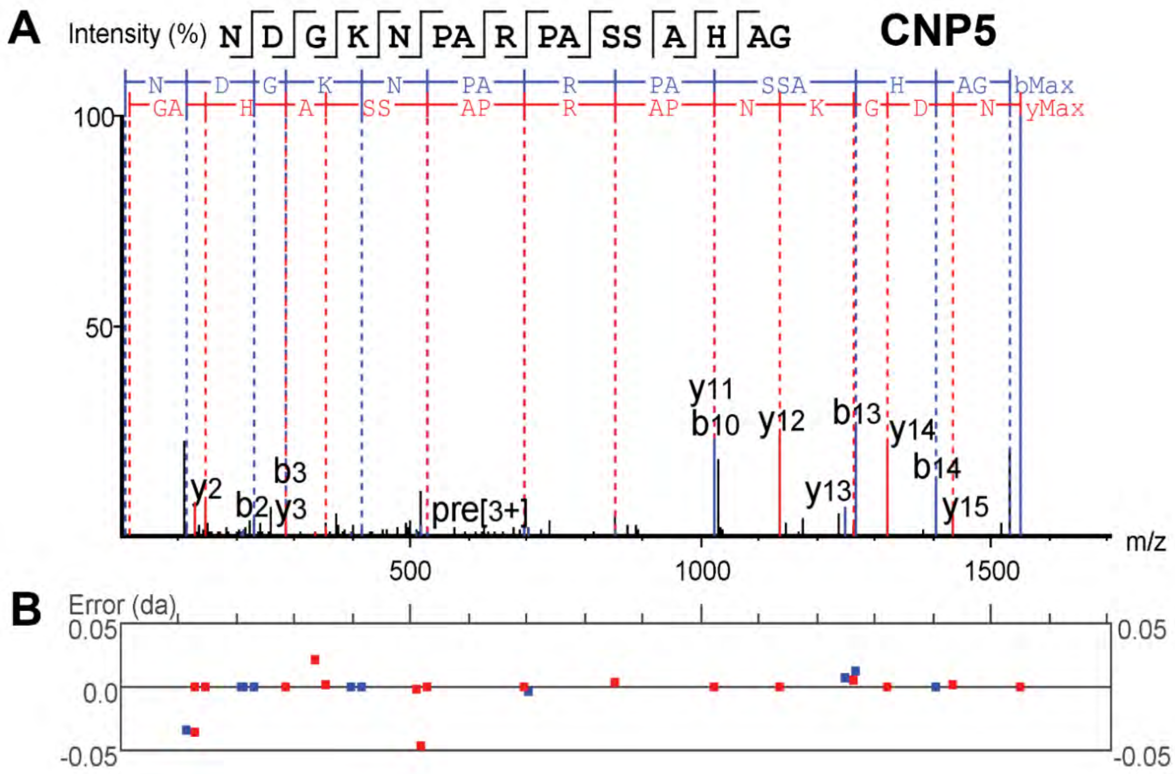

C

Ion Table

#	b	b-H2O	b-NH3	b (2+)	Seq	y	y-H2O	y-NH3	y (2+)	#
1	115.09	97.04	98.02	58.03	N					16
2	230.08	212.07	213.05	115.54	D	1435.70	1417.69	1418.68	718.35	15
3	287.10	269.09	270.07	144.05	G	1320.68	1302.67	1303.65	660.84	14
4	415.19	397.18	398.17	208.10	K	1263.65	1245.64	1246.63	632.33	13
5	529.24	511.23	512.21	265.12	N	1135.56	1117.55	1118.53	568.28	12
6	626.29	608.28	609.26	313.64	P	1021.52	1003.51	1004.49	511.26	11
7	697.33	679.32	680.30	349.16	A	924.46	906.45	907.44	462.73	10
8	853.42	835.42	836.40	427.21	R	853.42	835.42	836.40	427.21	9
9	950.48	932.47	933.45	475.74	P	697.33	679.32	680.30	349.16	8
10	1021.52	1003.51	1004.49	511.26	A	600.27	582.26	583.25	300.64	7
11	1108.55	1090.54	1091.52	554.77	S	529.24	511.23	512.21	265.12	6
12	1195.58	1177.57	1178.55	598.29	S	442.20	424.19	425.18	221.60	5
13	1266.61	1248.60	1249.59	633.81	A	355.17	337.14	338.15	178.09	4
14	1403.68	1385.67	1386.65	702.34	H	284.13	266.12	267.11	142.57	3
15	1474.72	1456.70	1457.69	737.86	A	147.08	129.10	130.05	74.04	2
16					G	76.04	58.03	59.01	38.52	1

-10lgP: 19.60; Mass: 1548.7393; Length: 16; ppm: 0.9; m/z: 517.2542; z: 3; RT: 19.60; Scan:3532;

DH-31

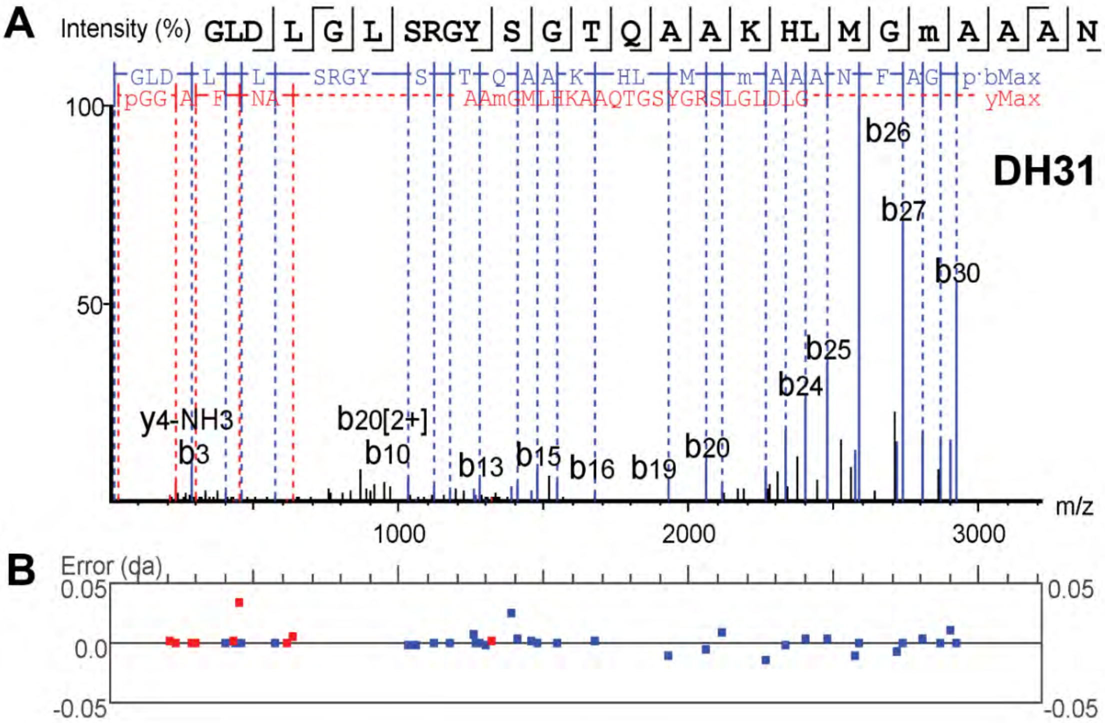

C

Ion Table

2	171.11	153.10	154.09	86.06	L	2977.48	2959.47	2960.45	1489.24	30
3	286.14	268.13	269.11	143.57	D	2864.39	2846.38	2847.37	1432.70	29
4	399.22	381.21	382.20	200.11	L	2749.37	2731.36	2732.34	1375.18	28
5	456.25	438.23	439.22	228.62	G	2636.28	2618.27	2619.25	1318.64	27
6	569.33	551.32	552.30	285.16	L	2579.26	2561.25	2562.23	1290.13	26
7	656.36	638.35	639.33	328.68	S	2466.18	2448.17	2449.15	1233.59	25
8	812.46	794.45	795.44	406.73	R	2379.14	2361.13	2362.12	1190.07	24
9	869.48	851.47	852.46	435.24	G	2223.04	2205.03	2206.02	1112.02	23
10	1032.55	1014.54	1015.52	516.77	Y	2166.02	2148.01	2148.99	1083.51	22
11	1119.58	1101.57	1102.55	560.29	S	2002.96	1984.95	1985.93	1001.98	21
12	1176.60	1158.59	1159.57	588.80	G	1915.93	1897.92	1898.90	958.46	20
13	1277.65	1259.63	1260.62	639.32	T	1858.91	1840.89	1841.88	929.95	19
14	1405.70	1387.67	1388.68	703.35	Q	1757.86	1739.85	1740.83	879.43	18
15	1476.74	1458.73	1459.72	738.87	A	1629.80	1611.79	1612.77	815.40	17
16	1547.78	1529.77	1530.75	774.39	A	1558.76	1540.75	1541.73	779.88	16
17	1675.87	1657.87	1658.85	838.44	K	1487.72	1469.71	1470.70	744.36	15
18	1812.94	1794.93	1795.91	906.97	H	1359.63	1341.62	1342.60	680.31	14
19	1926.03	1908.01	1908.99	963.51	L	1222.57	1204.56	1205.54	611.79	13
20	2057.07	2039.05	2040.03	1029.03	M	1109.49	1091.48	1092.46	555.24	12
21	2114.07	2096.07	2097.05	1057.54	G	978.45	960.44	961.42	489.72	11
22	2261.13	2243.11	2244.09	1131.06	M(+15.99)	921.42	903.41	904.40	461.21	10
23	2332.16	2314.14	2315.13	1166.58	A	774.39	756.38	757.36	387.69	9
24	2403.19	2385.18	2386.16	1202.10	A	703.35	685.34	686.33	352.18	8
25	2474.22	2456.22	2457.20	1237.61	A	632.31	614.30	615.29	316.66	7
26	2588.27	2570.26	2571.26	1294.64	N	561.28	543.27	544.25	281.14	6
27	2735.34	2717.33	2718.32	1368.17	F	447.20	429.22	430.21	224.12	5
28	2806.37	2788.37	2789.35	1403.69	A	300.17	282.16	283.14	150.58	4
29	2863.40	2845.39	2846.37	1432.20	G	229.13	211.12	212.10	115.06	3
30	2920.42	2902.40	2903.39	1460.71	G	172.11	154.10	155.08	86.55	2
31					P(-.98)	115.09	97.08	98.06	58.04	1

-10lgP:75.91; Mass: 3033.4912; Length: 31; ppm: 2.3 ; m/z: 759.3818 ; z: 2; RT: 59.42 ; Scan:11724;

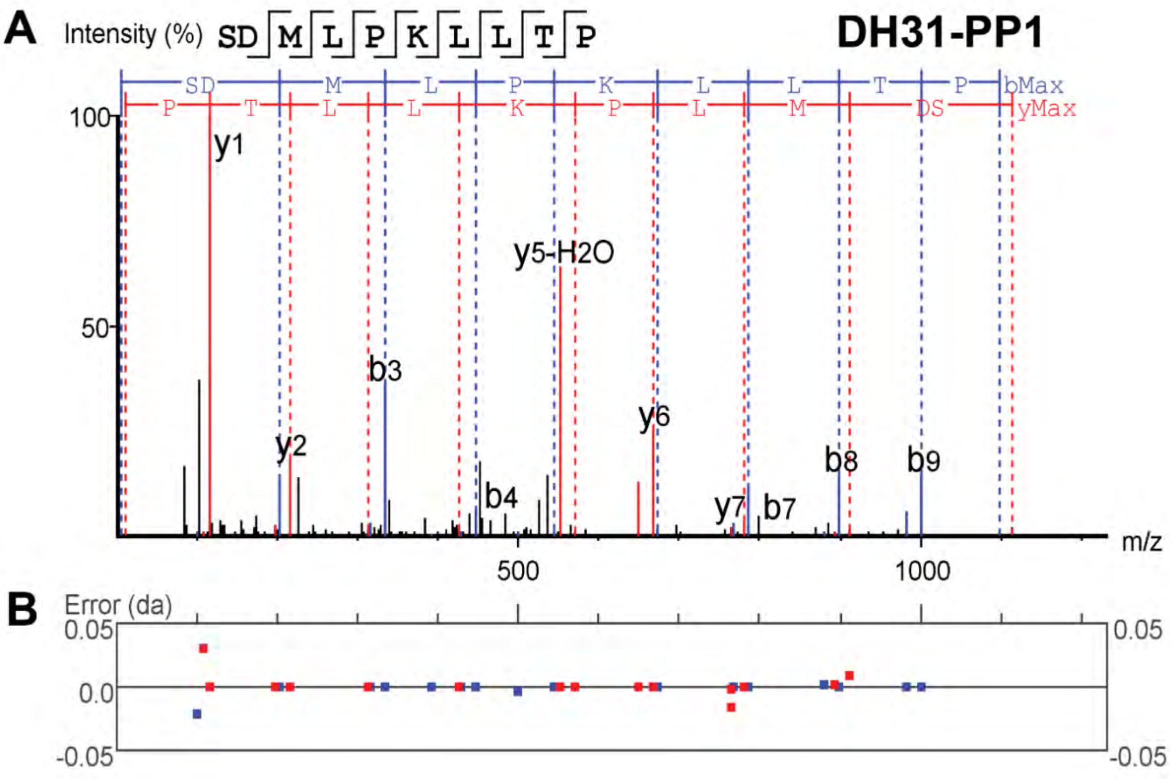

C

Ion Table

#	b	b-H2O	b-NH3	b (2+)	Seq	y	y-H2O	y-NH3	y (2+)	#
1	88.04	70.03	71.01	44.52	S					10
2	203.07	185.06	186.04	102.06	D	1027.59	1009.58	1010.56	514.29	9
3	334.11	316.10	317.08	167.55	M	912.55	894.55	895.53	456.78	8
4	447.19	429.18	430.16	224.10	L	781.52	763.51	764.51	391.26	7
5	544.24	526.23	527.22	272.62	P	668.43	650.42	651.41	334.72	6
6	672.34	654.33	655.31	336.67	K	571.38	553.37	554.35	286.19	5
7	785.42	767.41	768.40	393.21	L	443.29	425.28	426.26	222.14	4
8	898.51	880.49	881.48	449.75	L	330.20	312.19	313.18	165.60	3
9	999.55	981.54	982.53	500.28	T	217.12	199.11	200.09	109.03	2
10					P	116.07	98.06	99.04	58.54	1

-10lgP: 66.70; Mass: 1113.6104; Length: 10; ppm: 5.2; m/z: 557.8154 ; z: 2; RT: 63.20 ; Scan: 12643;

FMRF

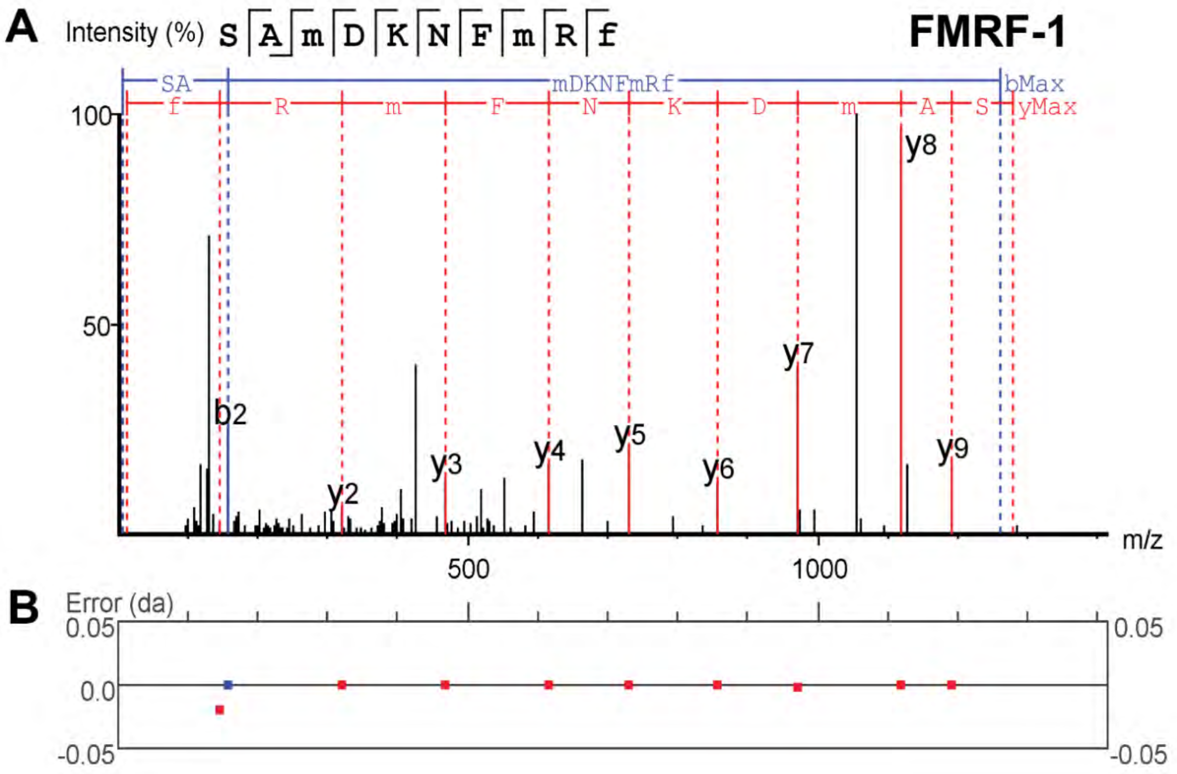

C

Ion Table

#	b	b-H2O	b-NH3	b (2+)	Seq	y	y-H2O	y-NH3	y (2+)	#
1	88.04	70.03	71.01	44.52	S					10
2	159.08	141.07	142.05	80.04	A	1190.54	1172.53	1173.52	595.77	9
3	306.11	288.10	289.09	153.56	M(+15.99)	1119.51	1101.50	1102.48	560.25	8
4	421.14	403.13	404.11	211.07	D	972.48	954.46	955.45	486.74	7
5	549.23	531.22	532.21	275.12	K	857.44	839.43	840.42	429.22	6
6	663.28	645.27	646.25	332.14	N	729.35	711.34	712.32	365.18	5
7	810.35	792.34	793.32	405.67	F	615.31	597.30	598.28	308.15	4
8	957.38	939.37	940.35	479.19	M(+15.99)	468.24	450.23	451.21	234.62	3
9	1113.48	1095.47	1096.46	557.24	R	321.20	303.19	304.18	161.10	2
10					F(-.98)	165.10	147.11	148.08	83.05	1

-10lgP: 34.34; Mass: 1276.5692; Length: 10; ppm: -0.1; m/z: 426.5303; z: 3; RT: 34.57; Scan: 6525

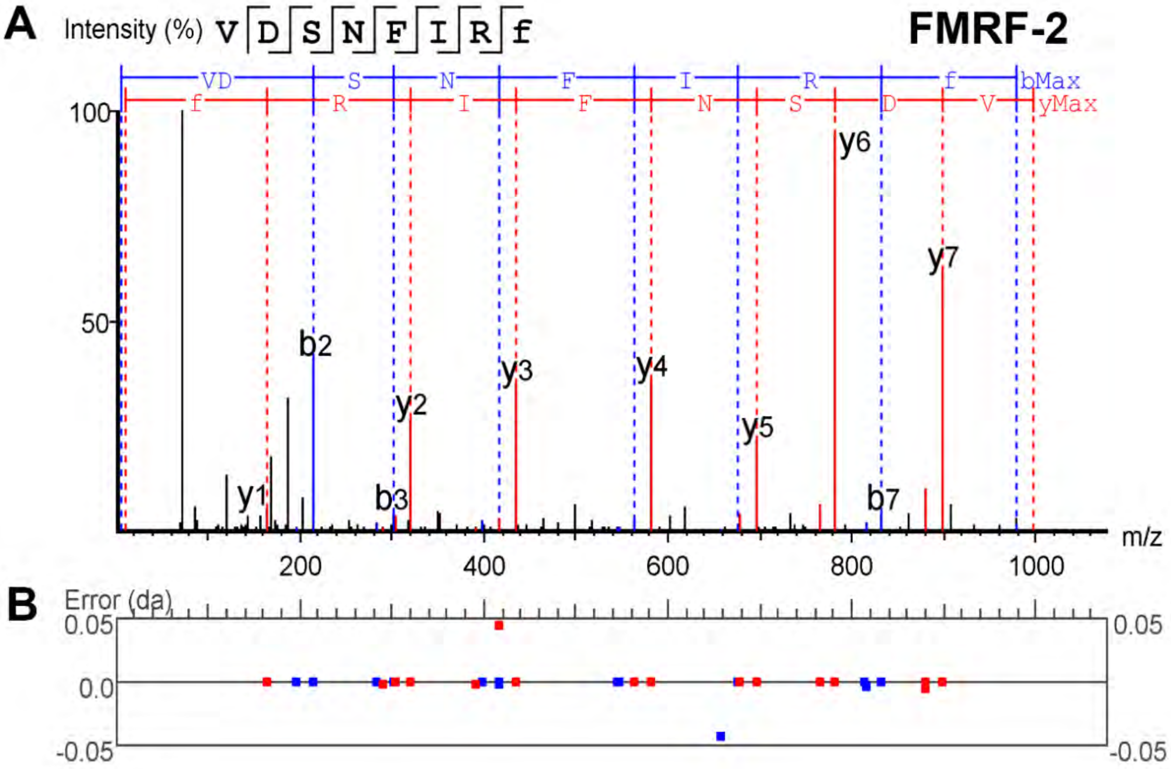

C

Ion Table

#	b	b-H2O	b-NH3	b (2+)	Seq	y	y-H2O	y-NH3	y (2+)	#
1	100.08	82.07	83.05	50.54	V					8
2	215.10	197.09	198.08	108.05	D	897.46	879.45	880.44	449.23	7
3	302.13	284.12	285.11	151.57	S	782.43	764.42	765.40	391.72	6
4	416.18	398.17	399.15	208.59	N	695.40	677.39	678.37	348.20	5
5	563.25	545.24	546.22	282.12	F	581.36	563.35	564.33	291.18	4
6	676.33	658.36	659.30	338.67	I	434.29	416.28	417.21	217.64	3
7	832.43	814.42	815.41	416.72	R	321.20	303.19	304.18	161.10	2
8					F(-.98)	165.10	147.09	148.08	83.05	1

-10lgP: 64.01; Mass: 995.5189; Length: 8; ppm: 0.4; m/z: 498.7669; z: 2; RT: 53.99; Scan: 10669

4.7 IDL

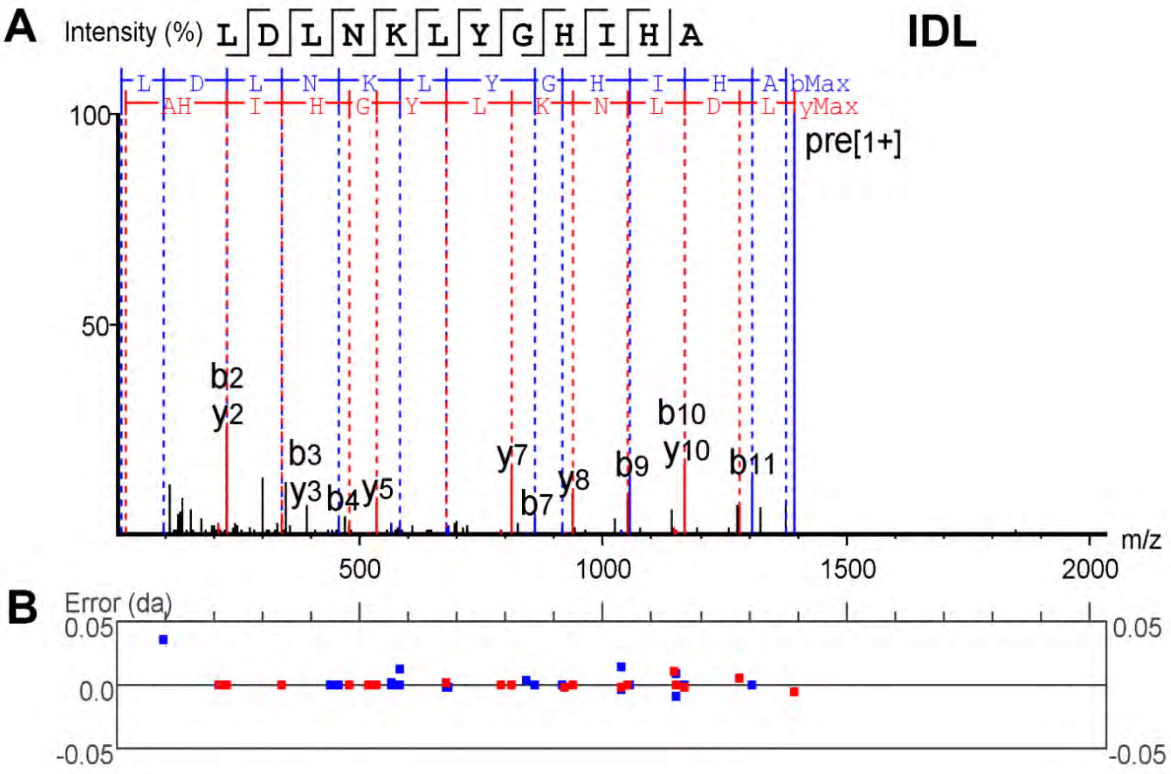

C

Ion Table

#	b	b-H2O	b-NH3	b (2+)	Seq	y	y-H2O	y-NH3	y (2+)	#
1	114.09	96.08	97.03	57.55	L					12
2	229.12	211.11	212.09	115.06	D	1280.67	1262.66	1263.65	640.84	11
3	342.20	324.19	325.18	171.60	L	1165.65	1147.63	1148.62	583.32	10
4	456.25	438.24	439.22	228.62	N	1052.56	1034.55	1035.54	526.78	9
5	584.33	566.33	567.31	292.67	K	938.52	920.51	921.49	469.76	8
6	697.42	679.42	680.40	349.21	L	810.43	792.41	793.40	405.71	7
7	860.49	842.48	843.46	430.74	Y	697.34	679.33	680.31	349.17	6
8	917.51	899.50	900.48	459.25	G	534.28	516.27	517.25	267.64	5
9	1054.57	1036.54	1037.55	527.78	H	477.26	459.25	460.23	239.13	4
10	1167.65	1149.63	1150.63	584.33	I	340.20	322.19	323.17	170.60	3
11	1304.71	1286.70	1287.68	652.86	H	227.11	209.10	210.09	114.06	2
12					A	90.05	72.04	73.03	45.53	1

-10lgP: 74.42; Mass: 1392.7513; Length: 12; ppm: -1.9; m/z: 697.3816; z: 2; RT: 51.57; Scan: 10153

4.8 MS

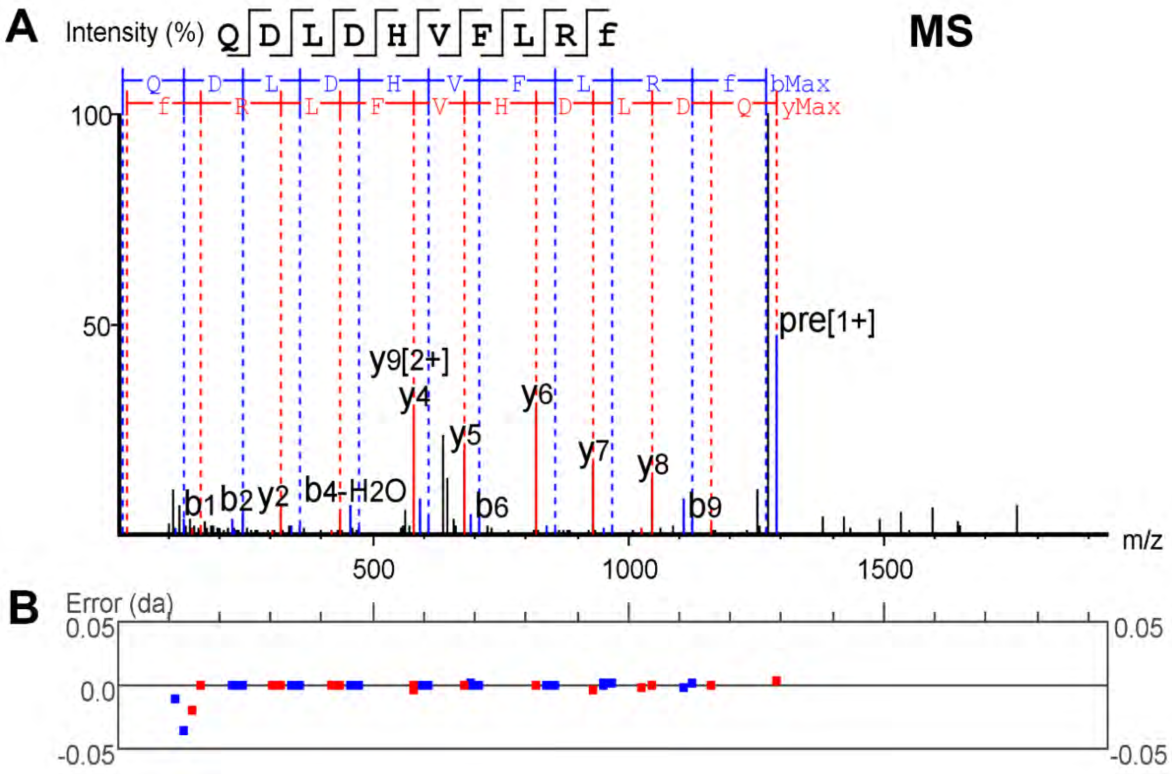

C

Ion Table

#	b	b-H2O	b-NH3	b (2+)	Seq	y	y-H2O	y-NH3	y (2+)	#
1	129.10	111.06	112.05	65.03	Q					10
2	244.09	226.08	227.07	122.55	D	1160.62	1142.61	1143.59	580.81	9
3	357.18	339.17	340.15	179.09	L	1045.59	1027.59	1028.57	523.30	8
4	472.20	454.19	455.18	236.60	D	932.51	914.50	915.48	466.76	7
5	609.26	591.25	592.24	305.13	H	817.48	799.47	800.46	409.24	6
6	708.33	690.32	691.30	354.67	V	680.42	662.41	663.40	340.71	5
7	855.40	837.39	838.37	428.20	F	581.36	563.35	564.33	291.18	4
8	968.48	950.47	951.46	484.74	L	434.29	416.28	417.26	217.64	3
9	1124.58	1106.57	1107.56	562.79	R	321.20	303.19	304.18	161.10	2
10					F(-.98)	165.10	147.11	148.08	83.05	1

-10lgP: 66.29; Mass: 1287.6724; Length: 10; ppm: 1.6; m/z: 644.8445; z: 2; RT: 60.53; Scan: 12057

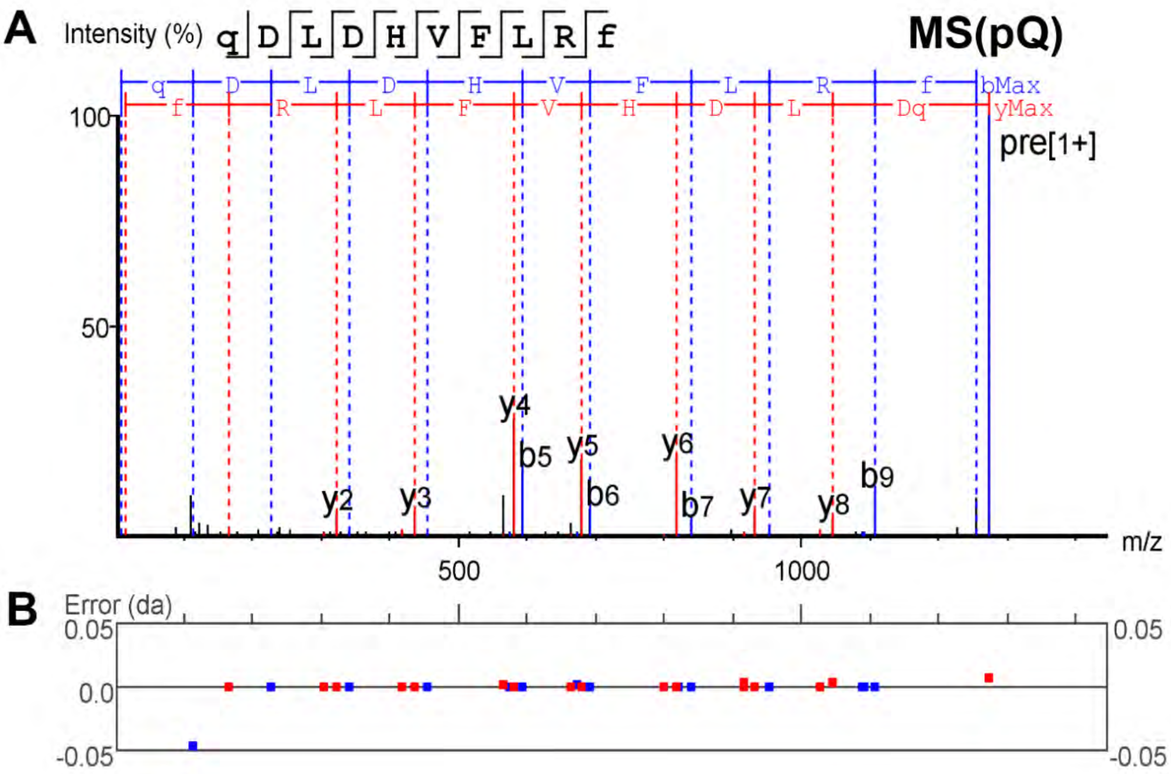

C

Ion Table

#	b	b-H2O	b-NH3	b (2+)	Seq	y	y-H2O	y-NH3	y (2+)	#
1	112.09	94.03	95.01	56.52	Q(-17.03)					10
2	227.07	209.06	210.04	114.03	D	1160.62	1142.61	1143.59	580.81	9
3	340.15	322.14	323.12	170.58	L	1045.59	1027.58	1028.57	523.30	8
4	455.18	437.17	438.15	228.09	D	932.51	914.50	915.48	466.76	7
5	592.24	574.23	575.21	296.62	H	817.48	799.47	800.46	409.24	6
6	691.30	673.29	674.28	346.15	V	680.42	662.41	663.40	340.71	5
7	838.37	820.36	821.35	419.69	F	581.35	563.35	564.33	291.18	4
8	951.46	933.45	934.43	476.23	L	434.29	416.28	417.26	217.64	3
9	1107.56	1089.55	1090.53	554.28	R	321.20	303.19	304.18	161.10	2
10					F(-.98)	165.10	147.09	148.08	83.05	1

-10lgP: 72.51; Mass: 1270.6459; Length: 10; ppm: 2.6; m/z: 636.3318; z: 2; RT: 68.94; Scan: 13845

4.9 Natalisin

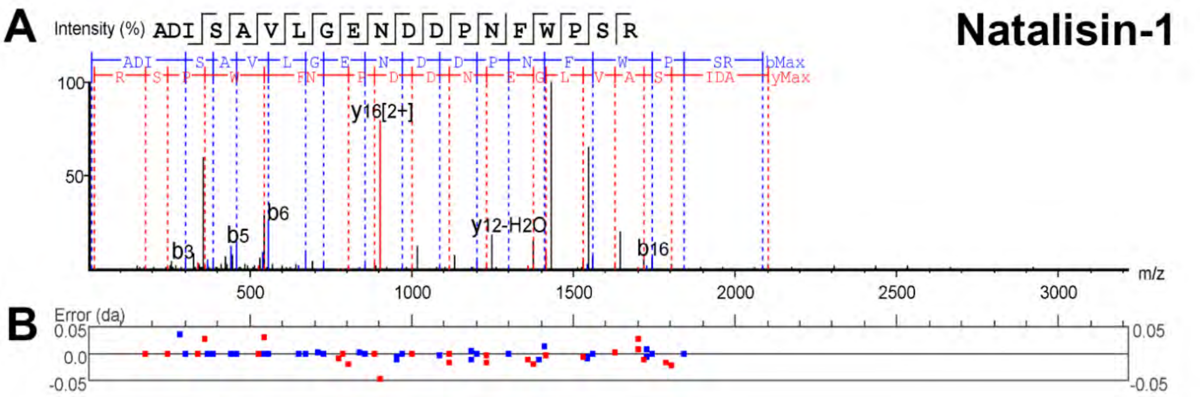

C

Ion Table

#	b	b-H2O	b-NH3	b (2+)	Seq	y	y-H2O	y-NH3	y (2+)	#
1	72.04	54.03	55.02	36.52	A					19
2	187.07	169.06	170.04	94.04	D	2031.94	2013.93	2014.91	1016.47	18
3	300.16	282.11	283.13	150.58	I	1916.91	1898.90	1899.89	958.96	17
4	387.19	369.18	370.16	194.09	S	1803.85	1785.84	1786.80	902.46	16
5	458.22	440.21	441.20	229.61	A	1716.81	1698.76	1699.76	858.90	15
6	557.29	539.28	540.27	279.15	V	1645.76	1627.75	1628.73	823.38	14
7	670.38	652.37	653.35	335.69	L	1546.69	1528.69	1529.67	773.86	13
8	727.40	709.39	710.37	364.20	G	1433.61	1415.60	1416.58	717.30	12
9	856.44	838.43	839.41	428.72	E	1376.61	1358.59	1359.56	688.79	11
10	970.48	952.48	953.47	485.74	N	1247.54	1229.54	1230.53	624.27	10
11	1085.52	1067.50	1068.48	543.26	D	1133.50	1115.49	1116.49	567.25	9
12	1200.54	1182.52	1183.52	600.77	D	1018.47	1000.46	1001.45	509.74	8
13	1297.59	1279.58	1280.56	649.30	P	903.45	885.44	886.42	452.22	7
14	1411.62	1393.62	1394.62	706.32	N	806.42	788.39	789.37	403.70	6
15	1558.70	1540.70	1541.68	779.85	F	692.35	674.34	675.32	346.68	5
16	1744.78	1726.76	1727.76	872.89	W	545.25	527.27	528.26	273.14	4
17	1841.84	1823.82	1824.81	921.42	P	359.17	341.19	342.18	180.10	3
18	1928.87	1910.86	1911.84	964.93	S	262.15	244.14	245.12	131.58	2
19					R	175.12	157.11	158.09	88.06	1

-10lgP: 63.12 Mass: 2101.9705; Length: 19; ppm: 10.4 m/z: 1052.0034; z: 2; RT: 62.81; Scan: 12535

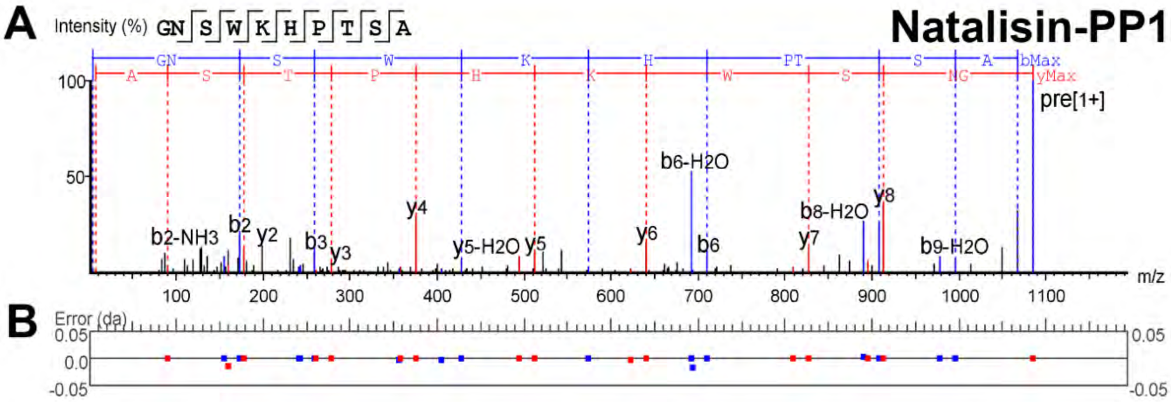

C

Ion Table

#	b	b-H2O	b-NH3	b (2+)	Seq	y	y-H2O	y-NH3	y (2+)	#
1	58.03	40.02	41.00	29.51	G					10
2	172.07	154.06	155.05	86.54	N	1027.50	1009.48	1010.47	514.25	9
3	259.10	241.09	242.08	130.05	S	913.45	895.44	896.43	457.23	8
4	445.18	427.17	428.16	223.09	W	826.42	808.41	809.39	413.71	7
5	573.28	555.27	556.25	287.14	K	640.34	622.33	623.31	320.67	6
6	710.34	692.33	693.33	355.67	H	512.25	494.24	495.22	256.62	5
7	807.39	789.38	790.36	404.20	P	375.19	357.18	358.16	188.09	4
8	908.44	890.42	891.41	454.72	T	278.13	260.12	261.11	139.57	3
9	995.47	977.46	978.44	498.23	S	177.09	159.09	160.06	89.04	2
10					A	90.06	72.04	73.03	45.53	1

-10lgP: 67.48 Mass: 1083.5098; Length: 10; ppm: 1.1 m/z: 542.7628; z: 2; RT: 26.68; Scan: 4975

4.10 NPLP-1

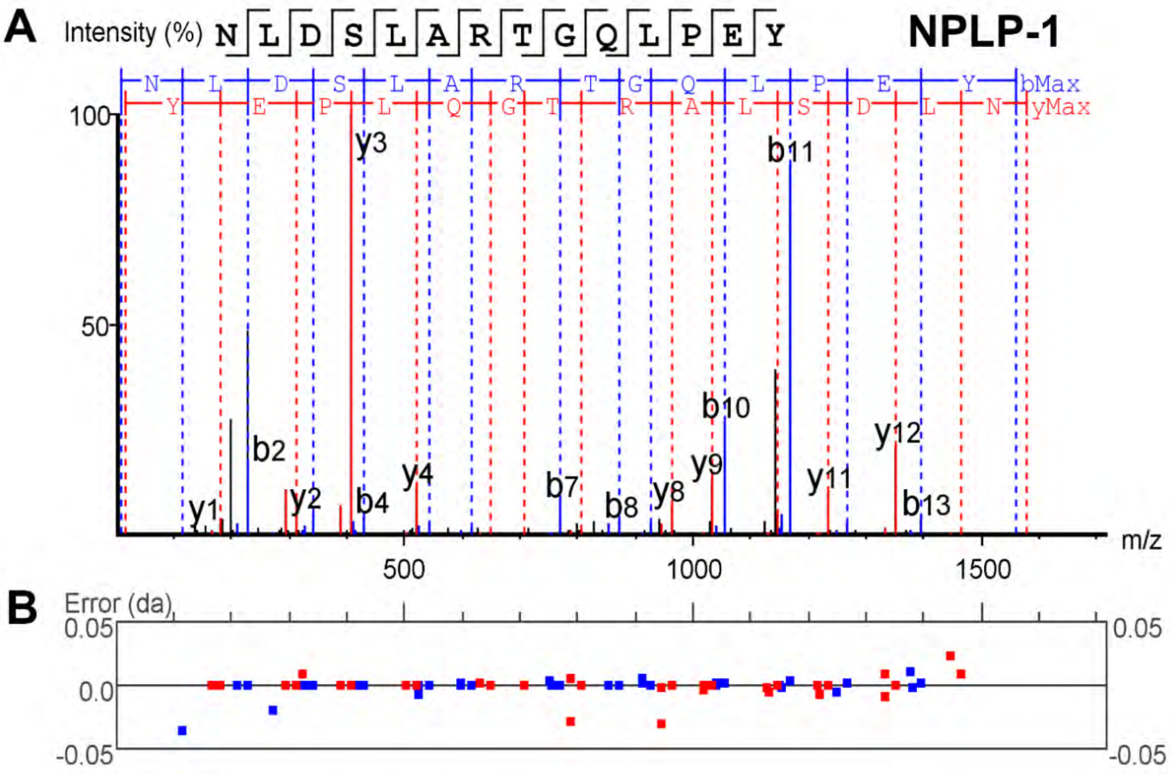

C

Ion Table

#	b	b-H2O	b-NH3	b (2+)	Seq	y	y-H2O	y-NH3	y (2+)	#
1	115.09	97.04	98.02	58.03	N					14
2	228.13	210.12	211.11	114.57	L	1462.74	1444.74	1445.70	731.88	13
3	343.16	325.15	326.13	172.08	D	1349.67	1331.65	1332.65	675.33	12
4	430.19	412.18	413.17	215.60	S	1234.64	1216.63	1217.62	617.82	11
5	543.28	525.27	526.26	272.16	L	1147.61	1129.60	1130.59	574.31	10
6	614.31	596.30	597.29	307.66	A	1034.53	1016.52	1017.50	517.76	9
7	770.41	752.40	753.39	385.71	R	963.49	945.51	946.46	482.24	8
8	871.46	853.45	854.44	436.23	T	807.39	789.41	790.36	404.19	7
9	928.48	910.47	911.45	464.74	G	706.34	688.33	689.31	353.67	6
10	1056.54	1038.53	1039.52	528.77	Q	649.32	631.31	632.29	325.15	5
11	1169.62	1151.62	1152.60	585.31	L	521.26	503.25	504.23	261.13	4
12	1266.68	1248.67	1249.66	633.84	P	408.18	390.17	391.15	204.59	3
13	1395.72	1377.70	1378.70	698.36	E	311.12	293.11	294.10	156.06	2
14					Y	182.08	164.07	165.05	91.54	1

-10lgP: 86.06; Mass: 1575.7893; Length: 14; ppm: 0.1; m/z: 788.9020; z: 2; RT: 53.94; Scan: 10656

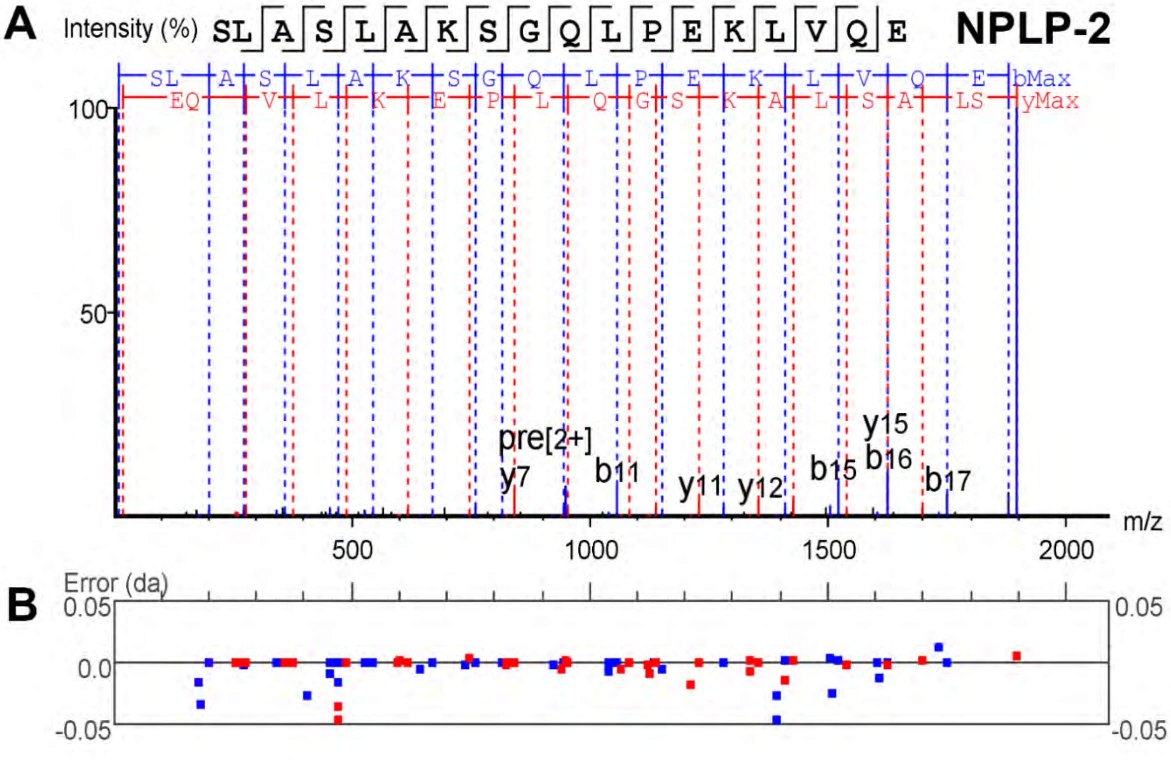

C Ion Table

#	b	b-H2O	b-NH3	b (2+)	Seq	y	y-H2O	y-NH3	y (2+)	#
1	88.04	70.03	71.01	44.52	S					18
2	201.12	183.15	184.10	101.06	L	1811.03	1793.02	1794.00	906.01	17
3	272.16	254.15	255.13	136.58	A	1697.94	1679.93	1680.92	849.47	16
4	359.19	341.18	342.17	180.11	S	1626.91	1608.90	1609.88	813.95	15
5	472.28	454.27	455.26	236.64	L	1539.88	1521.86	1522.85	770.44	14
6	543.31	525.30	526.29	272.16	A	1426.79	1408.78	1409.78	713.89	13
7	671.41	653.40	654.38	336.20	K	1355.75	1337.74	1338.73	678.38	12
8	758.44	740.43	741.41	379.72	S	1227.66	1209.65	1210.65	614.33	11
9	815.46	797.45	798.44	408.26	G	1140.63	1122.62	1123.61	570.81	10
10	943.52	925.51	926.49	472.28	Q	1083.60	1065.59	1066.58	542.30	9
11	1056.61	1038.59	1039.59	528.80	L	955.55	937.54	938.52	478.27	8
12	1153.66	1135.65	1136.63	577.33	P	842.46	824.45	825.44	421.73	7
13	1282.70	1264.69	1265.67	641.86	E	745.40	727.40	728.38	373.20	6
14	1410.79	1392.81	1393.82	705.90	K	616.37	598.35	599.34	308.68	5
15	1523.88	1505.87	1506.88	762.44	L	488.27	470.30	471.29	244.64	4
16	1622.95	1604.94	1605.93	811.97	V	375.19	357.18	358.16	188.09	3
17	1751.01	1732.98	1733.98	876.00	Q	276.12	258.11	259.09	138.56	2
18					E	148.06	130.05	131.03	74.53	1

-10lgP: 94.21; Mass: 1897.0520; Length: 18; ppm: 0.9; m/z: 949.5341; z: 2; RT: 54.32; Scan: 10729

A

B

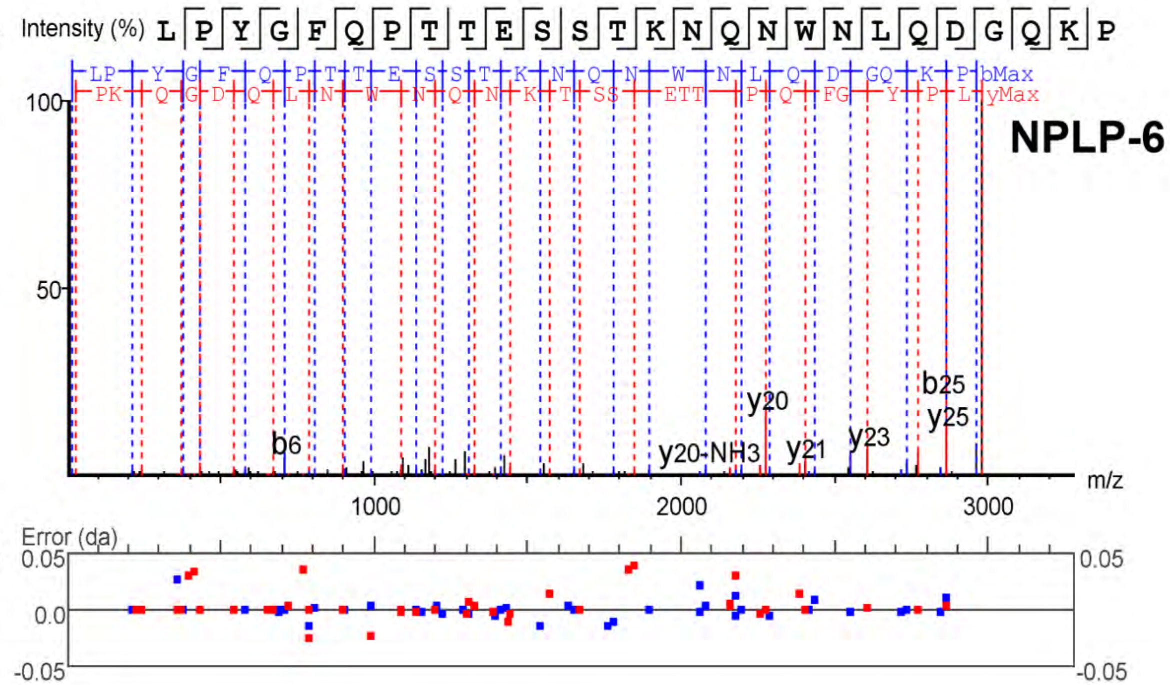

C

Ion Table

#	b	b-H2O	b-NH3	b (2+)	Seq	y	y-H2O	y-NH3	y (2+)	#
1	114.09	96.08	97.06	57.55	L					26
2	211.14	193.13	194.12	106.07	P	2865.34	2847.33	2848.32	1433.18	25
3	374.21	356.20	357.15	187.60	Y	2768.29	2750.28	2751.26	1384.65	24
4	431.23	413.22	414.20	216.11	G	2605.23	2587.22	2588.20	1303.12	23
5	578.30	560.29	561.27	289.65	F	2548.21	2530.20	2531.18	1274.60	22
6	706.36	688.34	689.33	353.68	Q	2401.14	2383.11	2384.11	1201.07	21
7	803.41	785.41	786.38	402.20	P	2273.08	2255.07	2256.06	1137.04	20
8	904.46	886.45	887.43	452.73	T	2176.00	2158.01	2158.99	1088.52	19
9	1005.50	987.49	988.48	503.25	T	2074.98	2056.97	2057.95	1037.99	18
10	1134.55	1116.54	1117.52	567.77	E	1973.93	1955.92	1956.90	987.49	17
11	1221.58	1203.56	1204.55	611.29	S	1844.85	1826.88	1827.83	922.94	16
12	1308.61	1290.60	1291.58	654.81	S	1757.86	1739.85	1740.83	879.43	15
13	1409.66	1391.65	1392.63	705.33	T	1670.82	1652.81	1653.80	835.91	14
14	1537.77	1519.74	1520.73	769.38	K	1569.76	1551.77	1552.75	785.41	13
15	1651.80	1633.78	1634.77	826.40	N	1441.69	1423.67	1424.65	721.34	12
16	1779.87	1761.84	1762.84	890.43	Q	1327.63	1309.62	1310.61	664.32	11
17	1893.90	1875.89	1876.87	947.45	N	1199.58	1181.57	1182.55	600.29	10
18	2079.97	2061.94	2062.95	1040.49	W	1085.54	1067.53	1068.51	543.27	9
19	2194.02	2176.00	2177.00	1097.51	N	899.46	881.45	882.43	450.23	8
20	2307.10	2289.09	2290.08	1154.05	L	785.41	767.37	768.39	393.18	7
21	2435.15	2417.15	2418.13	1218.08	Q	672.33	654.32	655.30	336.67	6
22	2550.19	2532.18	2533.16	1275.60	D	544.27	526.26	527.25	272.64	5
23	2607.21	2589.20	2590.18	1304.11	G	429.25	411.24	412.18	215.12	4
24	2735.27	2717.26	2718.25	1368.14	Q	372.23	354.21	355.20	186.61	3
25	2863.35	2845.35	2846.34	1432.18	K	244.17	226.15	227.14	122.58	2
26					P	116.07	98.06	99.04	58.54	1

-10lgP: 91.32; Mass: 2977.4207; Length: 26; ppm: -1.7; m/z: 993.4791; z: 3; RT: 51.83; Scan: 10206

4.11 NVP-like

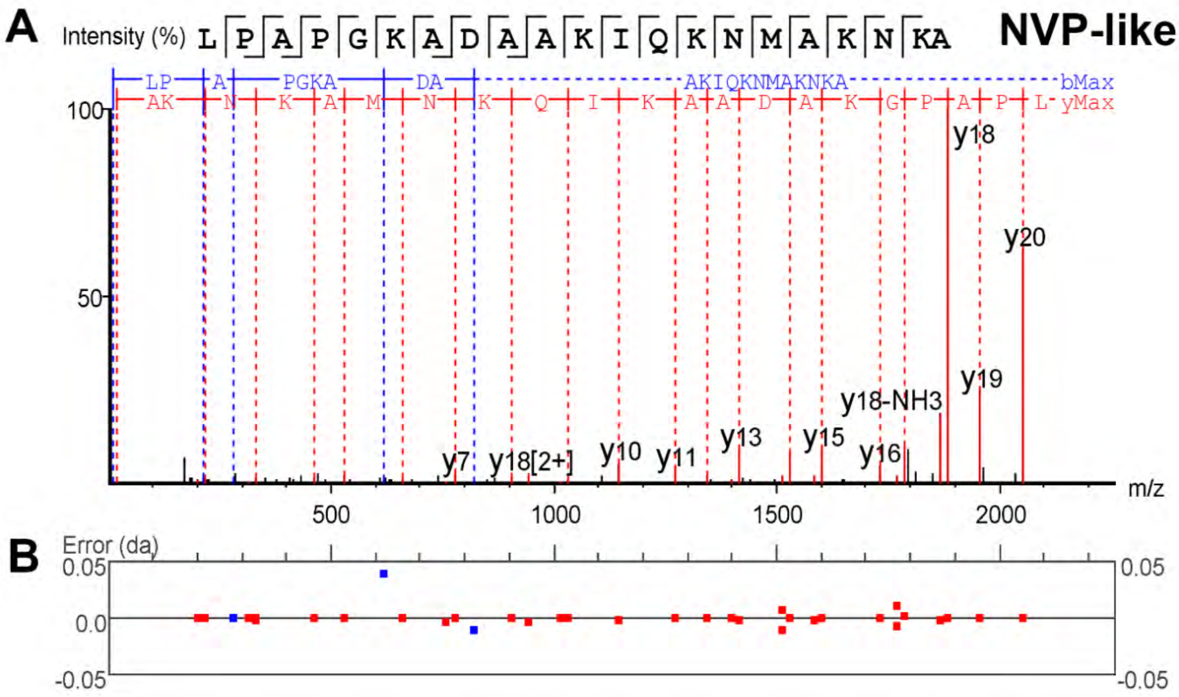

C

Ion Table

#	b	b-H2O	b-NH3	b (2+)	Seq	y	y-H2O	y-NH3	y (2+)	#
1	114.09	96.08	97.06	57.55	L					21
2	211.14	193.13	194.12	106.07	P	2052.14	2034.13	2035.11	1026.57	20
3	282.18	264.17	265.15	141.59	A	1955.08	1937.07	1938.06	978.04	19
4	379.23	361.22	362.21	190.12	P	1884.05	1866.04	1867.02	942.53	18
5	436.26	418.25	419.23	218.63	G	1786.99	1768.97	1769.98	894.00	17
6	564.35	546.34	547.32	282.68	K	1729.98	1711.96	1712.95	865.49	16
7	635.39	617.38	618.32	318.19	A	1601.88	1583.87	1584.85	801.44	15
8	750.41	732.40	733.39	375.71	D	1530.84	1512.82	1513.83	765.92	14
9	821.46	803.44	804.43	411.23	A	1415.82	1397.80	1398.79	708.41	13
10	892.49	874.48	875.46	446.74	A	1344.78	1326.77	1327.75	672.89	12
11	1020.58	1002.57	1003.56	510.79	K	1273.74	1255.73	1256.71	637.37	11
12	1133.67	1115.66	1116.64	567.33	I	1145.65	1127.64	1128.62	573.32	10
13	1261.73	1243.72	1244.70	631.36	Q	1032.56	1014.55	1015.54	516.78	9
14	1389.82	1371.81	1372.79	695.41	K	904.50	886.49	887.48	452.75	8
15	1503.86	1485.85	1486.84	752.43	N	776.41	758.40	759.39	388.70	7
16	1634.91	1616.89	1617.88	817.95	M	662.36	644.35	645.34	331.69	6
17	1705.94	1687.93	1688.92	853.47	A	531.32	513.31	514.30	266.16	5
18	1834.04	1816.03	1817.01	917.52	K	460.29	442.28	443.26	230.64	4
19	1948.08	1930.07	1931.05	974.54	N	332.19	314.18	315.17	166.60	3
20	2076.18	2058.16	2059.15	1038.59	K	218.15	200.14	201.12	109.57	2
21					A	90.05	72.04	73.03	45.53	1

-10lgP: 79.63; Mass: 2164.2148; Length: 21; ppm: 3.1; m/z: 433.8516; z: 5; RT: 33.79; Scan: 6471

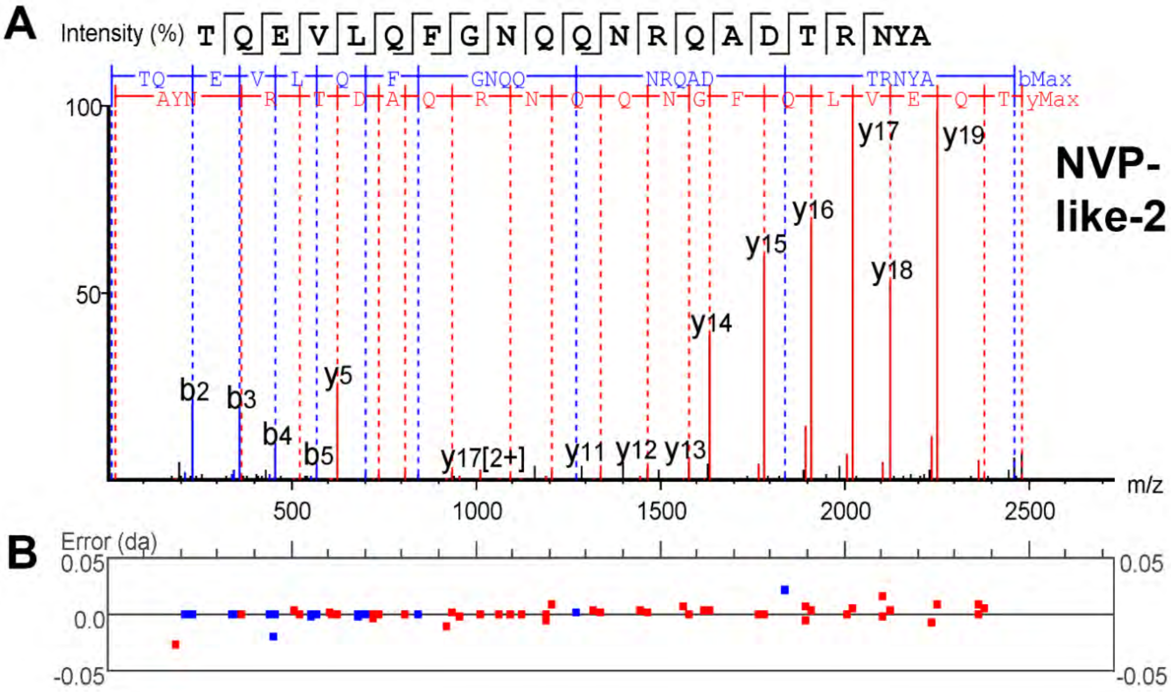

C

Ion Table

#	b	b-H2O	b-NH3	b (2+)	Seq	y	y-H2O	y-NH3	y (2+)	#
1	102.06	84.04	85.03	51.53	T					21
2	230.11	212.10	213.09	115.56	Q	2380.13	2362.13	2363.10	1190.57	20
3	359.16	341.14	342.13	180.08	E	2252.07	2234.07	2235.06	1126.54	19
4	458.22	440.21	441.20	229.61	V	2123.03	2105.01	2106.01	1062.02	18
5	571.31	553.30	554.28	286.15	L	2023.96	2005.96	2006.94	1012.49	17
6	699.37	681.36	682.34	350.18	Q	1910.88	1892.87	1893.86	955.94	16
7	846.44	828.43	829.41	423.72	F	1782.82	1764.82	1765.80	891.91	15
8	903.46	885.45	886.43	452.25	G	1635.75	1617.75	1618.73	818.38	14
9	1017.50	999.49	1000.47	509.25	N	1578.74	1560.73	1561.70	789.87	13
10	1145.56	1127.55	1128.53	573.28	Q	1464.69	1446.68	1447.66	732.85	12
11	1273.61	1255.61	1256.59	637.31	Q	1336.63	1318.62	1319.60	668.82	11
12	1387.66	1369.65	1370.63	694.33	N	1208.57	1190.57	1191.56	604.79	10
13	1543.76	1525.75	1526.73	772.38	R	1094.53	1076.52	1077.51	547.77	9
14	1671.82	1653.81	1654.79	836.41	Q	938.43	920.42	921.42	469.72	8
15	1742.86	1724.85	1725.83	871.93	A	810.38	792.36	793.35	405.69	7
16	1857.88	1839.85	1840.86	929.44	D	739.34	721.33	722.31	370.17	6
17	1958.93	1940.92	1941.91	979.97	T	624.31	606.30	607.28	312.65	5
18	2115.03	2097.02	2098.01	1058.02	R	523.26	505.25	506.23	262.13	4
19	2229.08	2211.07	2212.05	1115.04	N	367.16	349.15	350.13	184.11	3
20	2392.14	2374.13	2375.11	1196.57	Y	253.12	235.11	236.09	127.06	2
21					A	90.05	72.04	73.03	45.53	1

-10lgP: 62.34; Mass: 2480.1792; Length: 21; ppm: -1.8; m/z: 827.7322; z: 3; RT: 44.13; Scan: 8578

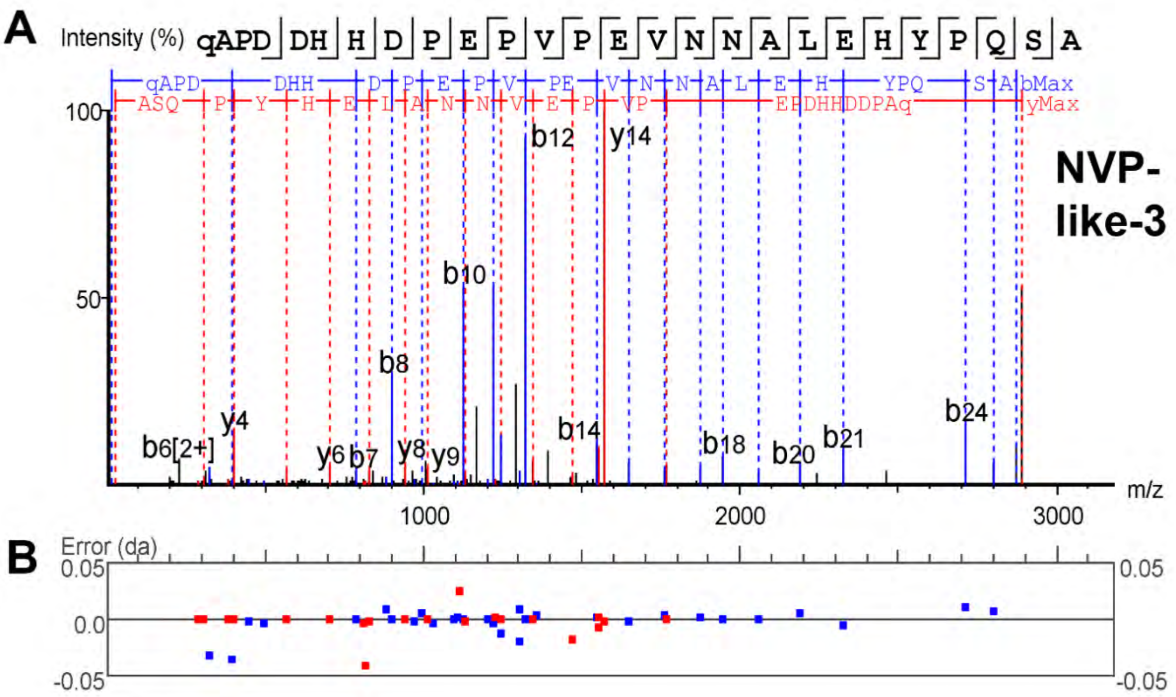

C

Ion Table

#	b	b-H2O	b-NH3	b (2+)	Seq	y	y-H2O	y-NH3	y (2+)	#
1	112.04	94.03	95.01	56.52	Q(-17.03)					26
2	183.08	165.07	166.05	92.04	A	2778.24	2760.23	2761.21	1389.62	25
3	280.13	262.12	263.10	140.56	P	2707.20	2689.19	2690.17	1354.10	24
4	395.19	377.15	378.13	198.08	D	2610.15	2592.14	2593.12	1305.57	23
5	510.18	492.17	493.16	255.59	D	2495.12	2477.11	2478.09	1248.06	22
6	647.24	629.23	630.22	324.16	H	2380.10	2362.08	2363.07	1190.55	21
7	784.30	766.29	767.27	392.65	H	2243.04	2225.03	2226.01	1122.02	20
8	899.33	881.31	882.30	450.17	D	2105.98	2087.97	2088.95	1053.49	19
9	996.38	978.37	979.35	498.69	P	1990.95	1972.94	1973.92	995.98	18
10	1125.42	1107.41	1108.40	563.21	E	1893.90	1875.89	1876.87	947.45	17
11	1222.48	1204.47	1205.45	611.74	P	1764.86	1746.84	1747.83	882.93	16
12	1321.54	1303.53	1304.54	661.27	V	1667.80	1649.79	1650.78	834.40	15
13	1418.60	1400.59	1401.57	709.80	P	1568.74	1550.73	1551.70	784.87	14
14	1547.64	1529.63	1530.61	774.32	E	1471.70	1453.67	1454.65	736.34	13
15	1646.71	1628.70	1629.68	823.85	V	1342.64	1324.63	1325.61	671.82	12
16	1760.75	1742.74	1743.72	880.88	N	1243.57	1225.56	1226.54	622.29	11
17	1874.79	1856.78	1857.77	937.90	N	1129.53	1111.49	1112.50	565.26	10
18	1945.83	1927.82	1928.80	973.42	A	1015.48	997.47	998.46	508.24	9
19	2058.92	2040.91	2041.89	1029.96	L	944.45	926.44	927.42	472.72	8
20	2187.95	2169.95	2170.93	1094.48	E	831.37	813.36	814.38	416.18	7
21	2325.02	2307.01	2307.99	1163.01	H	702.32	684.31	685.29	351.66	6
22	2488.08	2470.07	2471.05	1244.55	Y	565.26	547.25	548.23	283.13	5
23	2585.13	2567.12	2568.11	1293.07	P	402.20	384.19	385.17	201.60	4
24	2713.18	2695.18	2696.16	1357.09	Q	305.15	287.13	288.12	153.07	3
25	2800.22	2782.21	2783.20	1400.61	S	177.09	159.08	160.06	89.04	2
26					A	90.05	72.04	73.03	45.53	1

-10lgP: 88.22; Mass:2888.2637; Length: 26; ppm: -1.8; m/z: 963.7601; z: 3; RT: 51.75; Scan: 10196

4.12 OKa

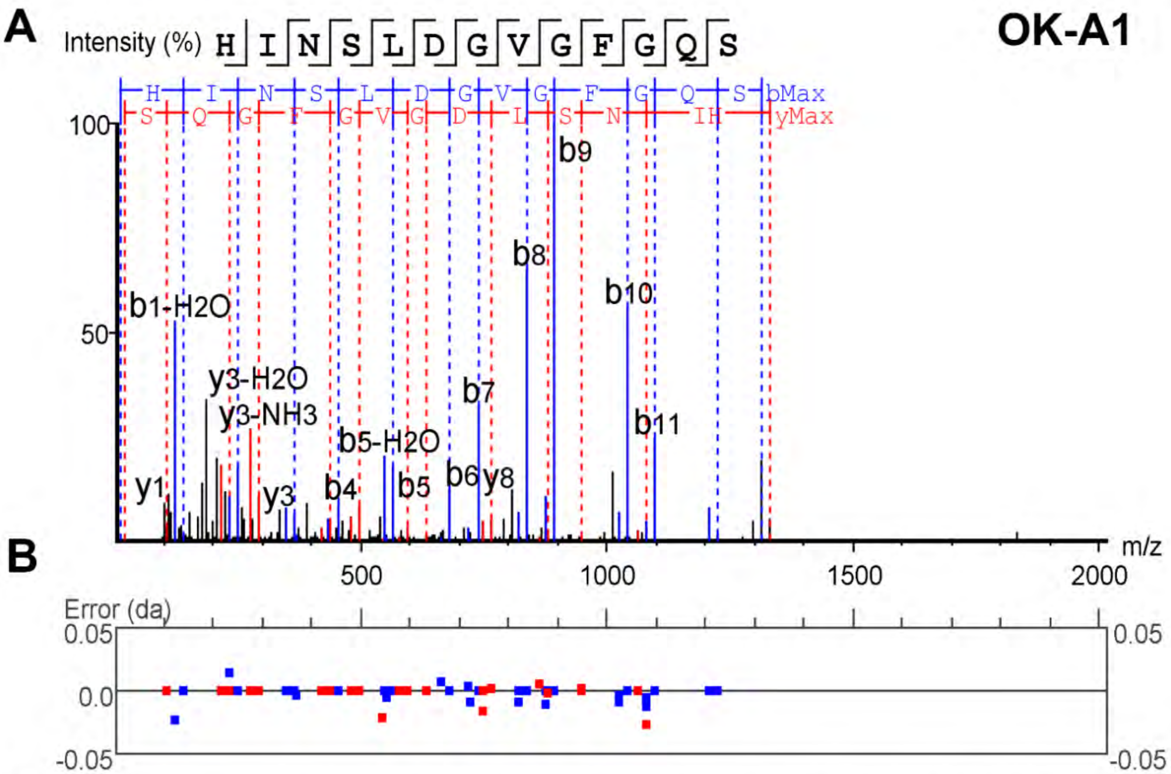

C

Ion Table

#	b	b-H2O	b-NH3	b (2+)	Seq	y	y-H2O	y-NH3	y (2+)	#
1	138.07	120.08	121.04	69.53	H					13
2	251.15	233.14	234.11	126.08	I	1193.58	1175.57	1176.55	597.29	12
3	365.19	347.18	348.17	183.10	N	1080.52	1062.48	1063.47	540.77	11
4	452.23	434.21	435.20	226.61	S	966.45	948.44	949.42	483.73	10
5	565.31	547.30	548.28	283.15	L	879.42	861.40	862.39	440.21	9
6	680.34	662.32	663.31	340.67	D	766.33	748.33	749.33	383.67	8
7	737.36	719.34	720.34	369.18	G	651.31	633.30	634.28	326.15	7
8	836.43	818.42	819.41	418.71	V	594.29	576.28	577.26	297.64	6
9	893.45	875.44	876.43	447.22	G	495.22	477.21	478.19	248.11	5
10	1040.52	1022.51	1023.50	520.76	F	438.20	420.19	421.17	219.60	4
11	1097.54	1079.53	1080.52	549.28	G	291.13	273.12	274.10	146.06	3
12	1225.59	1207.59	1208.57	613.30	Q	234.11	216.10	217.08	117.55	2
13					S	106.05	88.04	89.02	53.52	1

-10lgP: 83.93; Mass: 1329.6313; Length: 13; ppm: -1.0; m/z: 665.8223; z: 2; RT: 47.62; Scan: 9306

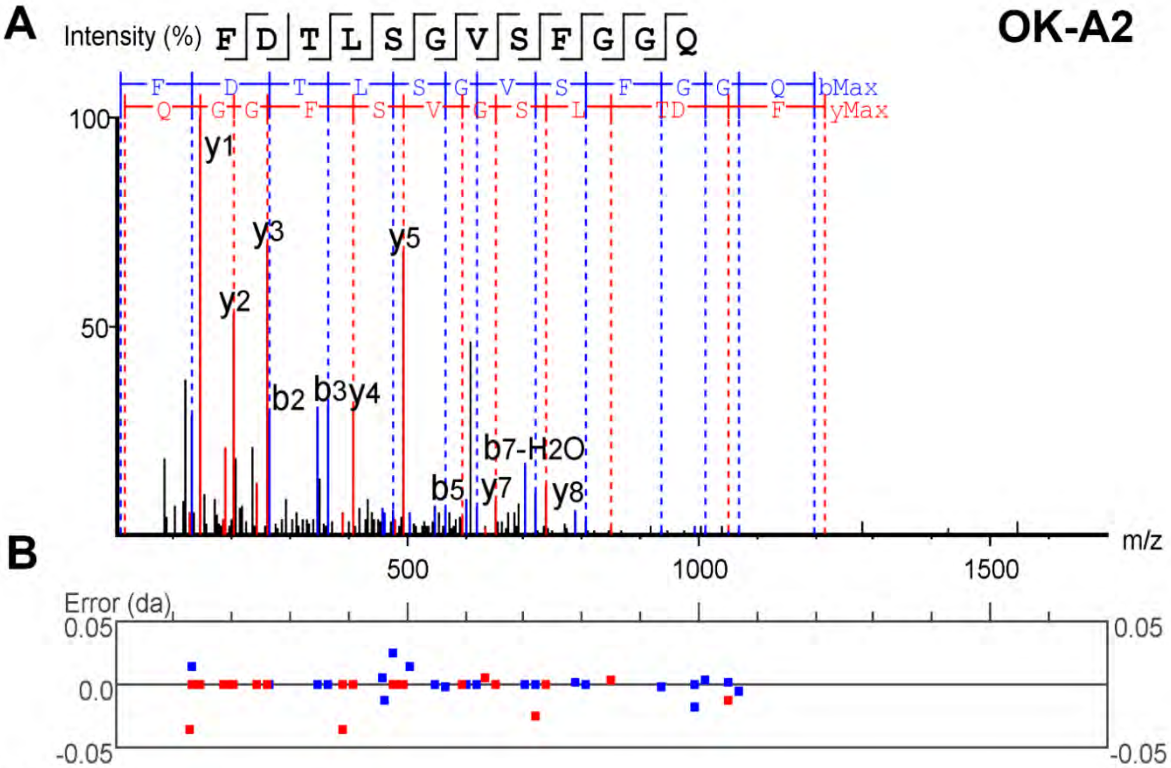

C

Ion Table

1	148.08	130.05	131.05	74.54	F					12
2	263.10	245.09	246.08	132.05	D	1067.50	1049.49	1050.49	534.25	11
3	364.15	346.14	347.12	182.58	T	952.47	934.46	935.45	476.74	10
4	477.21	459.22	460.22	239.12	L	851.42	833.42	834.40	426.21	9
5	564.27	546.26	547.24	282.63	S	738.34	720.36	721.31	369.67	8
6	621.29	603.28	604.26	311.14	G	651.31	633.29	634.28	326.15	7
7	720.36	702.35	703.33	360.68	V	594.29	576.28	577.26	297.64	6
8	807.39	789.38	790.36	404.19	S	495.22	477.21	478.19	248.11	5
9	954.46	936.45	937.43	477.73	F	408.19	390.18	391.20	204.59	4
10	1011.47	993.47	994.47	506.22	G	261.12	243.11	244.09	131.06	3
11	1068.51	1050.49	1051.47	534.75	G	204.10	186.09	187.07	102.55	2
12					Q	147.08	129.10	130.05	74.04	1

-10lgP: 74.94; Mass: 1213.5615; Length: 12; ppm: 0.7; m/z: 607.7885; z: 2; RT: 58.55; Scan: 11630

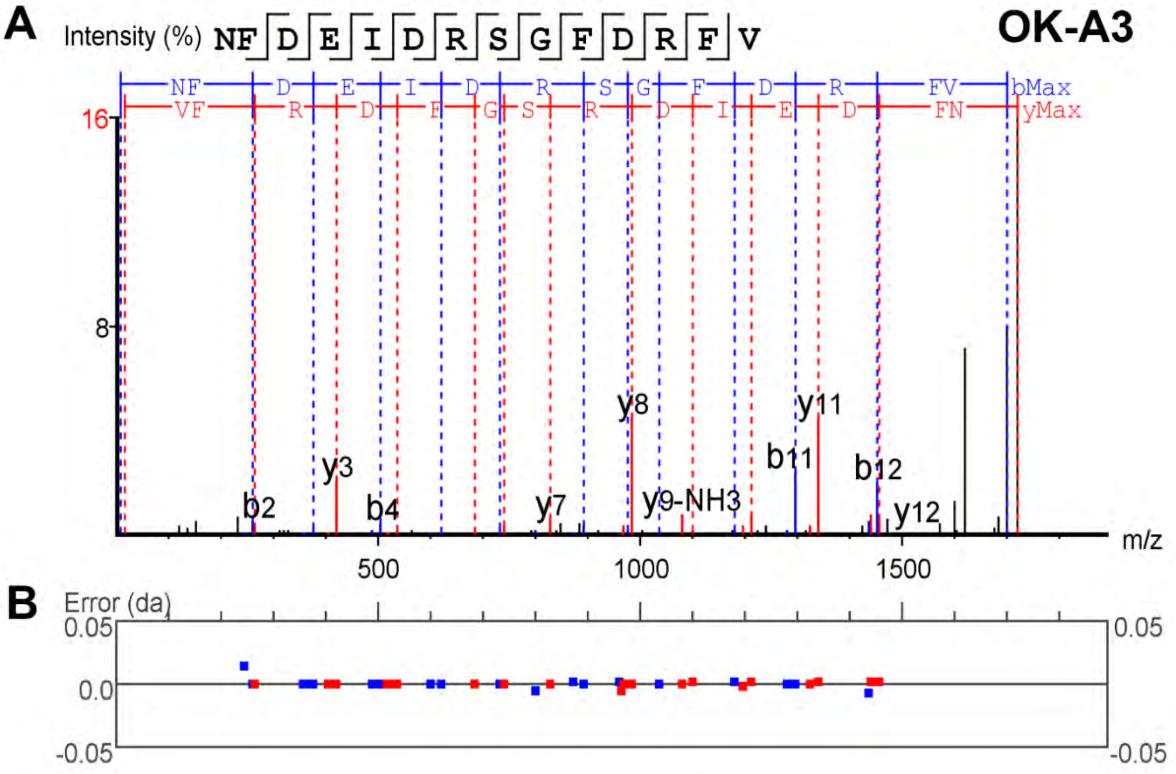

C

Ion Table

#	b	b-H2O	b-NH3	b (2+)	Seq	y	y-H2O	y-NH3	y (2+)	#
1	115.05	97.04	98.02	58.03	N					14
2	262.12	244.11	245.08	131.56	F	1602.75	1584.74	1585.73	801.88	13
3	377.15	359.13	360.12	189.07	D	1455.68	1437.68	1438.66	728.34	12
4	506.19	488.18	489.16	253.59	E	1340.66	1322.65	1323.63	670.83	11
5	619.27	601.26	602.25	310.14	I	1211.61	1193.61	1194.59	606.31	10
6	734.30	716.29	717.27	367.65	D	1098.53	1080.52	1081.51	549.77	9
7	890.40	872.39	873.37	445.70	R	983.50	965.50	966.48	492.25	8
8	977.43	959.42	960.40	489.22	S	827.40	809.39	810.38	414.20	7
9	1034.45	1016.44	1017.43	517.73	G	740.37	722.36	723.35	370.69	6
10	1181.52	1163.51	1164.50	591.26	F	683.35	665.34	666.32	342.18	5
11	1296.55	1278.54	1279.52	648.77	D	536.28	518.27	519.26	268.64	4
12	1452.65	1434.64	1435.63	726.83	R	421.26	403.25	404.23	211.13	3
13	1599.72	1581.71	1582.69	800.37	F	265.15	247.14	248.13	133.08	2
14					V	118.09	100.08	101.06	59.54	1

-10lgP: 71.52; Mass: 1715.7903; Length: 14; ppm: 0.8; m/z: 858.9031; z: 3; RT: 58.65; Scan: 11731

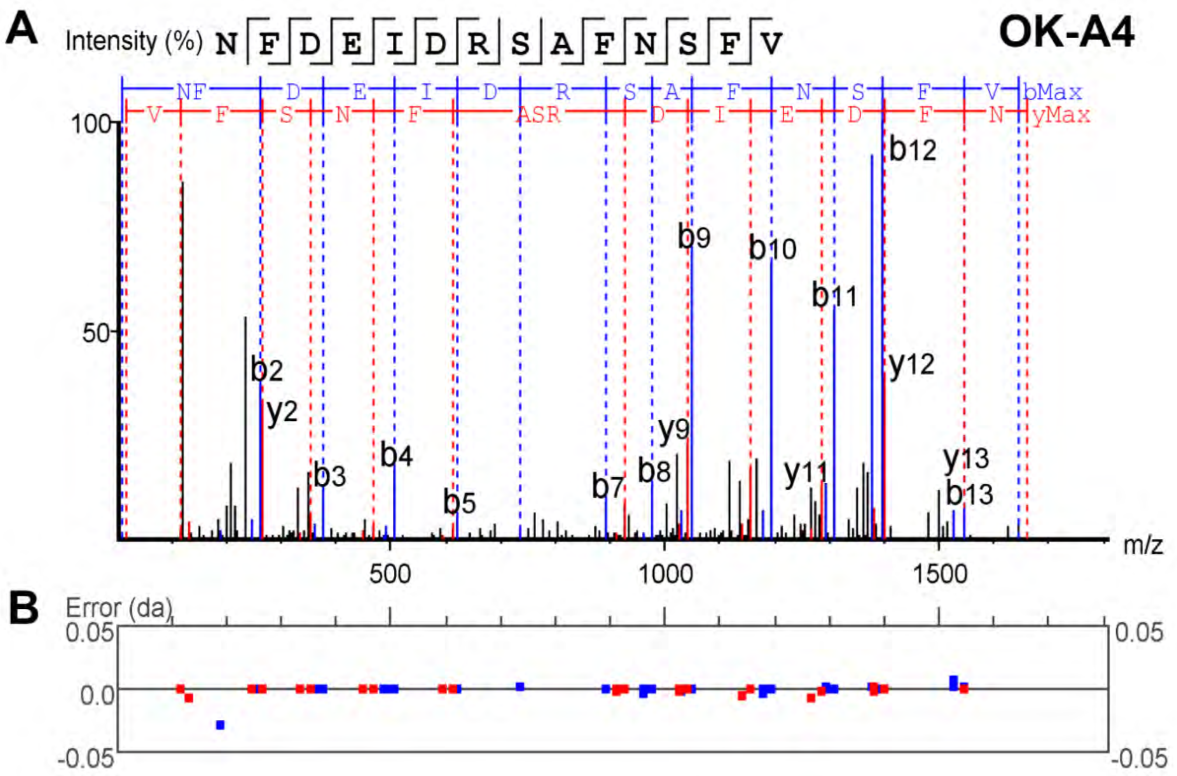

C

Ion Table

#	b	b-H2O	b-NH3	b (2+)	Seq	y	y-H2O	y-NH3	y (2+)	#
1	115.05	97.04	98.02	58.03	N					14
2	262.12	244.11	245.09	131.56	F	1546.72	1528.71	1529.69	773.86	13
3	377.15	359.14	360.12	189.10	D	1399.65	1381.64	1382.63	700.32	12
4	506.19	488.18	489.16	253.59	E	1284.62	1266.62	1267.60	642.81	11
5	619.27	601.26	602.25	310.14	I	1155.58	1137.57	1138.56	578.29	10
6	734.30	716.29	717.27	367.65	D	1042.50	1024.48	1025.47	521.75	9
7	890.40	872.39	873.37	445.70	R	927.47	909.46	910.44	464.23	8
8	977.43	959.42	960.41	489.22	S	771.37	753.36	754.34	386.18	7
9	1048.47	1030.46	1031.45	524.73	A	684.34	666.32	667.31	342.67	6
10	1195.54	1177.53	1178.52	598.27	F	613.30	595.29	596.27	307.15	5
11	1309.58	1291.57	1292.56	655.29	N	466.23	448.22	449.20	233.61	4
12	1396.61	1378.60	1379.59	698.81	S	352.19	334.18	335.16	176.59	3
13	1543.68	1525.66	1526.65	772.34	F	265.15	247.14	248.13	133.09	2
14					V	118.09	100.08	101.06	59.54	1

-10lgP: 80.00; Mass:1659.7528; Length: 14; ppm: 3.6; m/z: 830.8867; z: 2; RT: 63.94; Scan: 12682

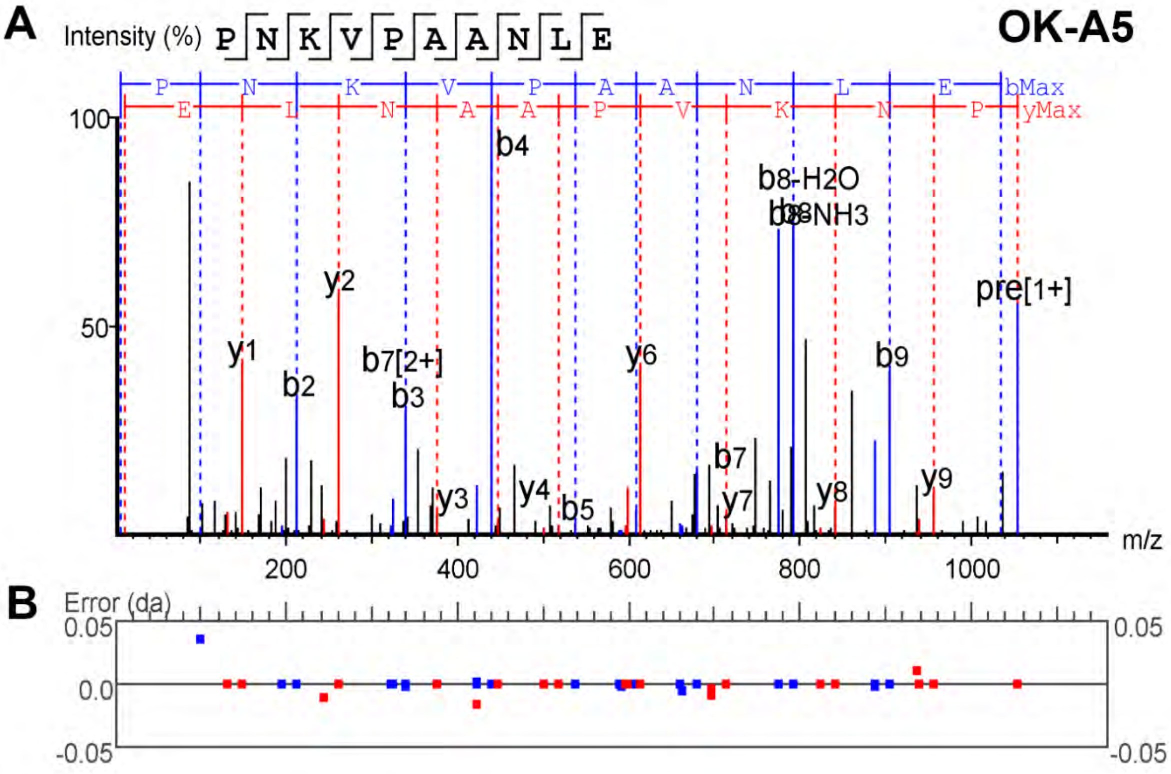

C Ion Table

#	b	b-H2O	b-NH3	b (2+)	Seq	y	y-H2O	y-NH3	y (2+)	#
1	98.02	80.05	81.03	49.53	P					10
2	212.10	194.09	195.08	106.55	N	955.52	937.50	938.49	478.26	9
3	340.20	322.19	323.17	170.60	K	841.48	823.47	824.45	421.26	8
4	439.27	421.26	422.24	220.13	V	713.38	695.38	696.37	357.19	7
5	536.32	518.31	519.29	268.66	P	614.31	596.30	597.29	307.66	6
6	607.36	589.35	590.33	304.18	A	517.26	499.25	500.24	259.13	5
7	678.40	660.38	661.37	339.70	A	446.22	428.21	429.20	223.61	4
8	792.44	774.42	775.41	396.72	N	375.19	357.18	358.16	188.09	3
9	905.52	887.51	888.50	453.26	L	261.14	243.15	244.12	131.07	2
10					E	148.06	130.05	131.03	74.53	1

-10lgP: 69.48; Mass:1051.5662; Length: 10; ppm: -2.8; m/z: 526.7889; z: 2; RT: 36.77; Scan: 7024

4.13 PDF

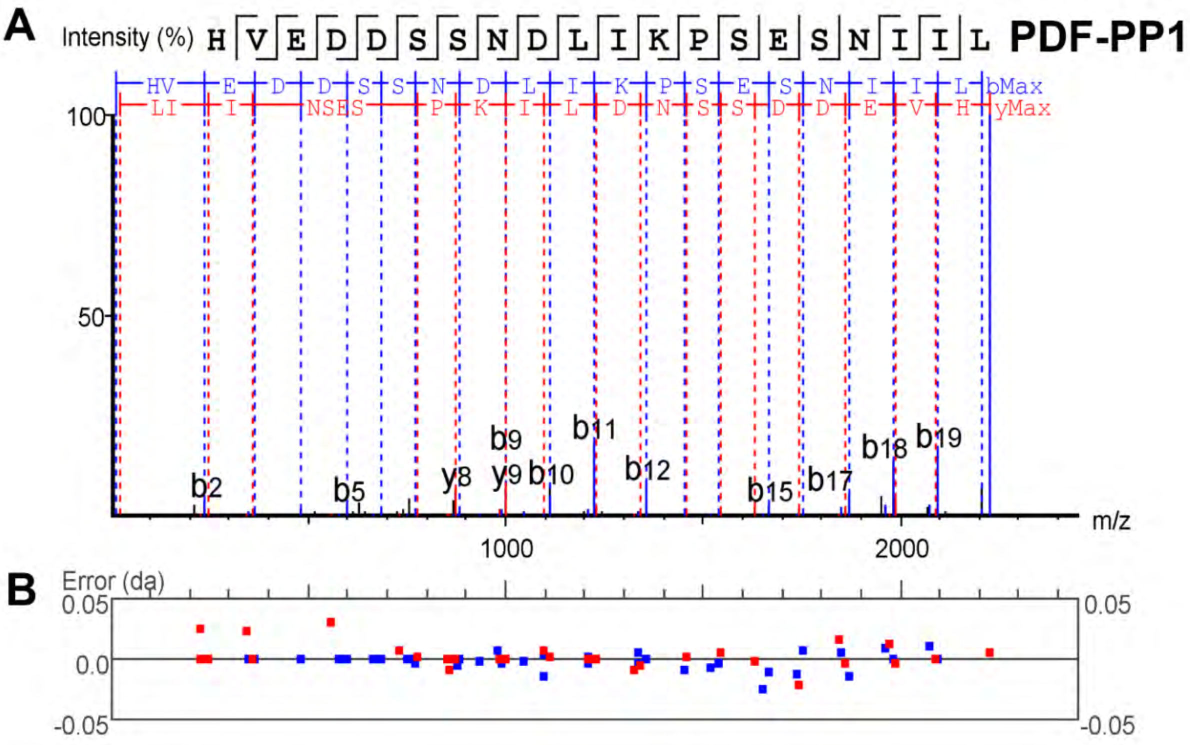

C

Ion Table

#	b	b-H2O	b-NH3	b (2+)	Seq	y	y-H2O	y-NH3	y (2+)	#
1	138.07	120.06	121.04	69.53	H					20
2	237.13	219.12	220.11	119.07	V	2088.04	2070.02	2071.01	1044.52	19
3	366.18	348.17	349.15	183.59	E	1988.97	1970.94	1971.94	994.98	18
4	481.20	463.19	464.18	241.10	D	1859.93	1841.91	1842.88	930.46	17
5	596.23	578.22	579.20	298.62	D	1744.92	1726.89	1727.87	872.95	16
6	683.26	665.25	666.24	342.13	S	1629.87	1611.86	1612.84	815.43	15
7	770.30	752.28	753.27	385.65	S	1542.83	1524.83	1525.81	771.92	14
8	884.34	866.33	867.31	442.67	N	1455.80	1437.79	1438.78	728.39	13
9	999.36	981.35	982.34	500.18	D	1341.77	1323.76	1324.74	671.38	12
10	1112.45	1094.44	1095.44	556.72	L	1226.73	1208.72	1209.71	613.87	11
11	1225.53	1207.52	1208.51	613.27	I	1113.65	1095.63	1096.62	557.29	10
12	1353.63	1335.61	1336.61	677.31	K	1000.57	982.56	983.54	500.78	9
13	1450.69	1432.67	1433.65	725.84	P	872.47	854.46	855.46	436.74	8
14	1537.72	1519.71	1520.69	769.36	S	775.42	757.41	758.39	388.21	7
15	1666.77	1648.75	1649.76	833.88	E	688.39	670.38	671.36	344.69	6
16	1753.78	1735.79	1736.76	877.40	S	559.34	541.33	542.32	280.17	5
17	1867.85	1849.82	1850.80	934.42	N	472.31	454.30	455.29	236.66	4
18	1980.91	1962.89	1963.89	990.96	I	358.27	340.26	341.22	179.64	3
19	2094.00	2075.98	2076.97	1047.50	I	245.19	227.17	228.13	123.09	2
20					L	132.10	114.09	115.07	66.55	1

-10lgP: 93.14; Mass: 2224.0859; Length: 20; ppm:1.4 m/z: 1113.0515; z: 2; RT: 57.38; Scan: 11489

4.14 PK

A

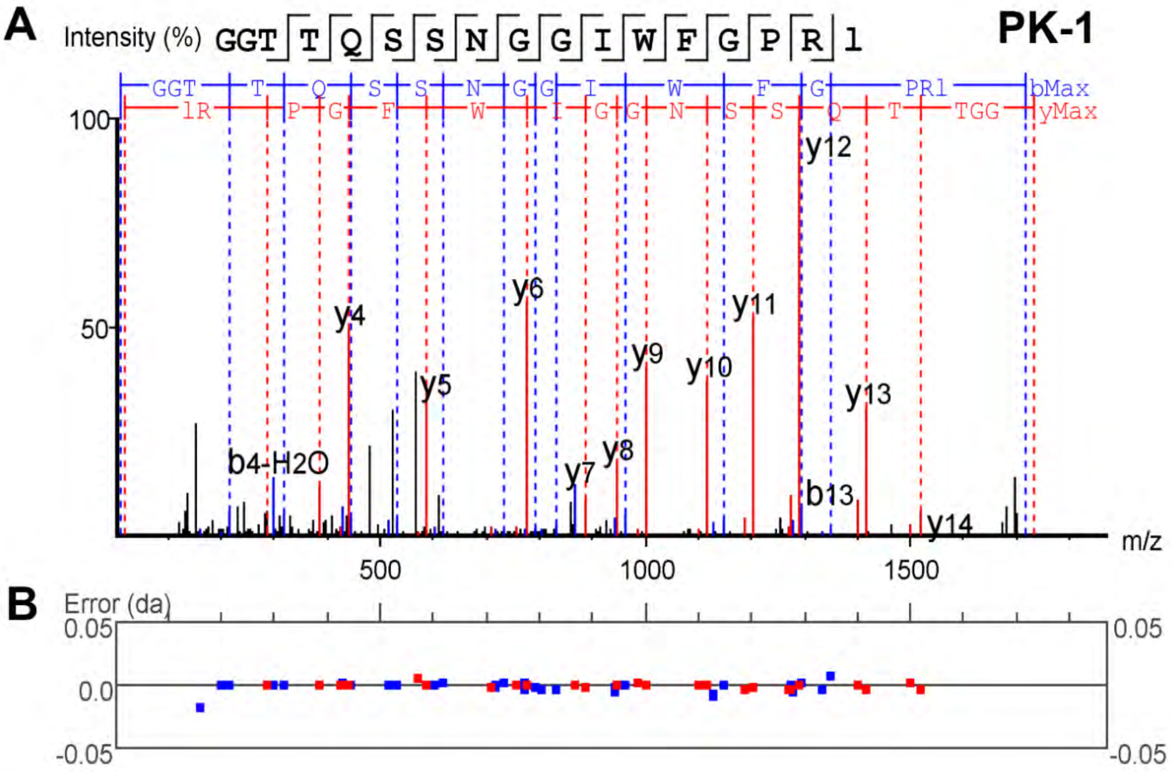

C

Ion Table

#	b	b-H2O	b-NH3	b (2+)	Seq	y	y-H2O	y-NH3	y (2+)	#
1	58.03	40.02	41.00	29.51	G					17
2	115.05	97.04	98.02	58.03	G	1676.85	1658.84	1659.82	838.93	16
3	216.10	198.09	199.07	108.55	T	1619.83	1601.82	1602.80	810.41	15
4	317.15	299.14	300.12	159.09	T	1518.79	1500.77	1501.75	759.89	14
5	445.20	427.19	428.18	223.10	Q	1417.74	1399.72	1400.71	709.37	13
6	532.24	514.22	515.21	266.62	S	1289.68	1271.67	1272.65	645.34	12
7	619.27	601.26	602.24	310.13	S	1202.65	1184.63	1185.62	601.82	11
8	733.31	715.30	716.29	367.16	N	1115.61	1097.60	1098.58	558.31	10
9	790.33	772.33	773.30	395.67	G	1001.57	983.56	984.54	501.28	9
10	847.35	829.34	830.33	424.18	G	944.55	926.54	927.52	472.77	8
11	960.44	942.43	943.42	480.72	I	887.53	869.51	870.50	444.26	7
12	1146.52	1128.52	1129.50	573.76	W	774.44	756.43	757.41	387.72	6
13	1293.58	1275.58	1276.57	647.29	F	588.36	570.35	571.33	294.68	5
14	1350.60	1332.60	1333.58	675.80	G	441.29	423.28	424.27	221.15	4
15	1447.66	1429.65	1430.63	724.33	P	384.27	366.26	367.24	192.64	3
16	1603.76	1585.75	1586.73	802.38	R	287.22	269.21	270.19	144.11	2
17					L(-.98)	131.12	113.11	114.09	66.06	1

-10lgP: 85.29; Mass:1732.8645; Length: 17; ppm: 2.7; m/z: 867.4419; z: 3; RT: 60.35; Scan: 12016

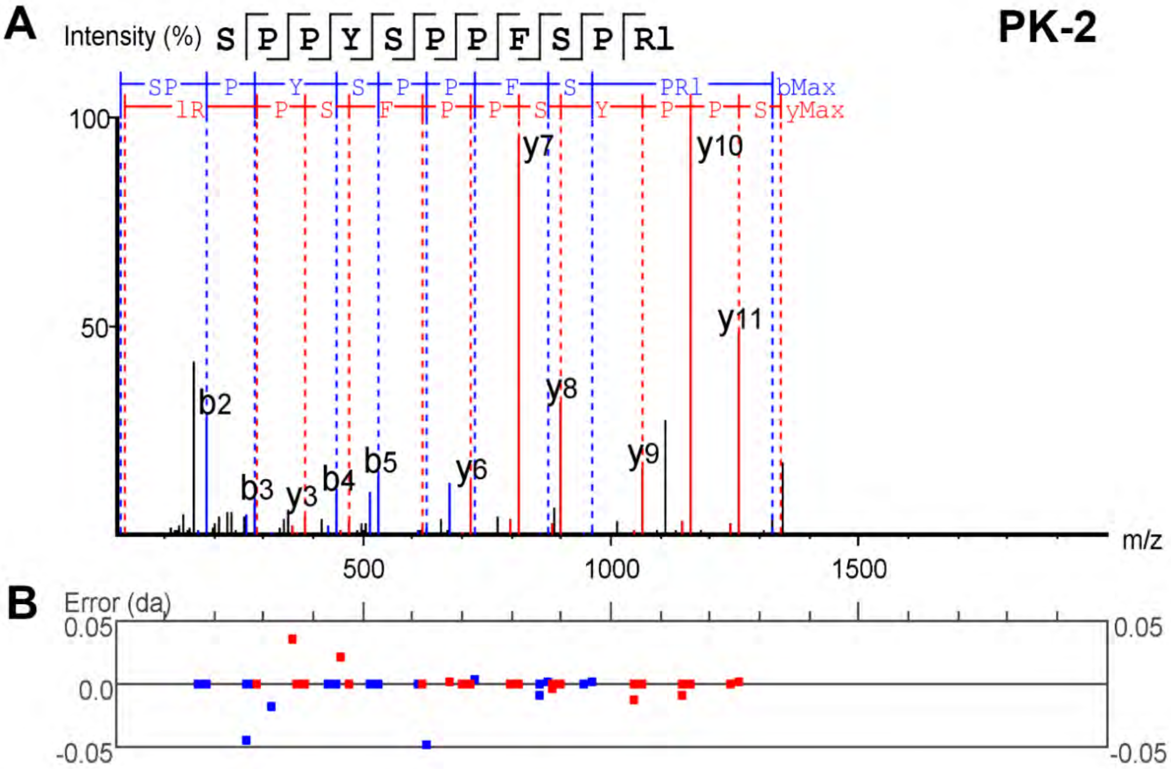

C

Ion Table

#	b	b-H2O	b-NH3	b (2+)	Seq	y	y-H2O	y-NH3	y (2+)	#
1	88.04	70.03	71.01	44.52	S					12
2	185.09	167.08	168.07	93.05	P	1256.68	1238.67	1239.65	628.84	11
3	282.14	264.13	265.16	141.57	P	1159.62	1141.62	1142.61	580.31	10
4	445.21	427.20	428.18	223.10	Y	1062.57	1044.56	1045.56	531.79	9
5	532.24	514.23	515.21	266.62	S	899.51	881.50	882.49	450.25	8
6	629.34	611.28	612.27	315.17	P	812.48	794.47	795.45	406.74	7
7	726.34	708.34	709.32	363.67	P	715.42	697.41	698.40	358.18	6
8	873.41	855.40	856.40	437.21	F	618.37	600.36	601.35	309.69	5
9	960.44	942.44	943.42	480.72	S	471.30	453.27	454.28	236.15	4
10	1057.50	1039.49	1040.47	529.25	P	384.27	366.26	367.25	192.64	3
11	1213.60	1195.59	1196.57	607.30	R	287.22	269.21	270.19	144.11	2
12					L(-.98)	131.12	113.11	114.09	66.06	1

-10lgP: 62.20; Mass:1342.7034; Length: 12; ppm: 2.2; m/z: 672.3604; z: 2; RT: 54.88; Scan: 10922

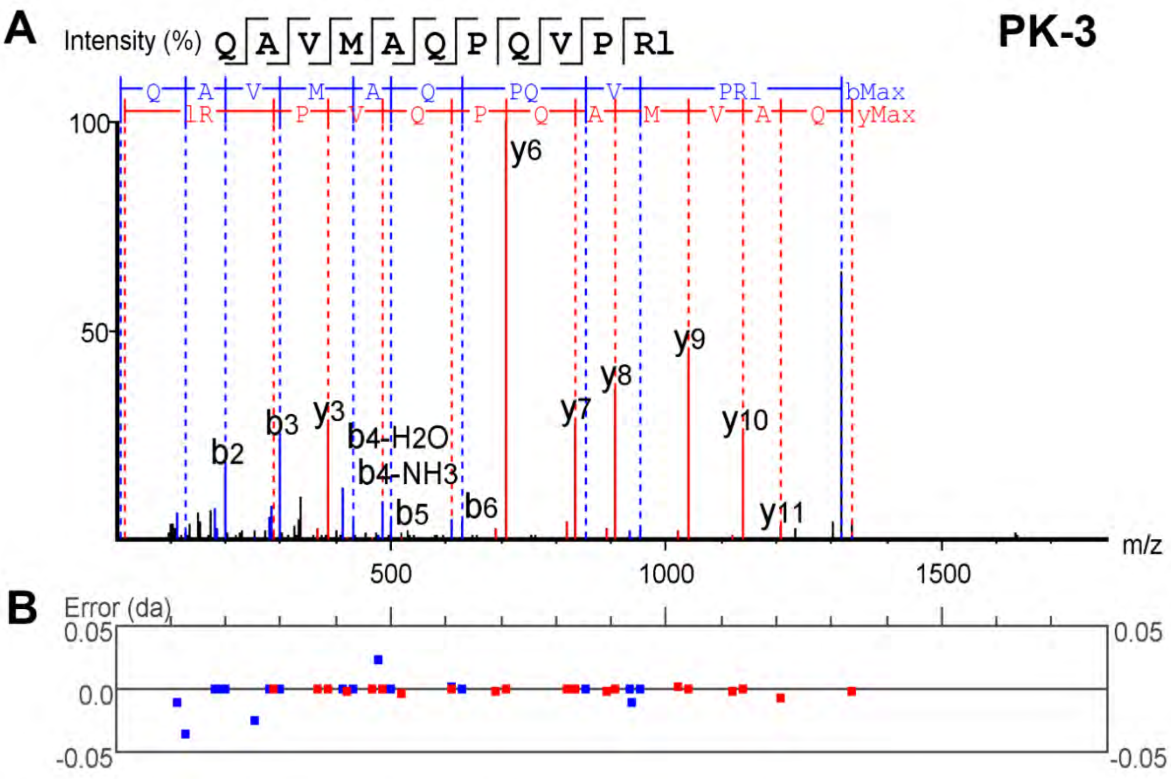

C

Ion Table

#	b	b-H2O	b-NH3	b (2+)	Seq	y	y-H2O	y-NH3	y (2+)	#
1	129.10	111.06	112.05	65.03	Q					12
2	200.10	182.09	183.08	100.55	A	1208.70	1190.68	1191.67	604.85	11
3	299.17	281.16	282.14	150.09	V	1137.66	1119.65	1120.63	569.33	10
4	430.21	412.20	413.18	215.61	M	1038.59	1020.58	1021.56	519.80	9
5	501.25	483.24	484.22	251.15	A	907.55	889.54	890.52	454.27	8
6	629.31	611.30	612.28	315.15	Q	836.51	818.50	819.48	418.76	7
7	726.36	708.35	709.33	363.68	P	708.45	690.44	691.43	354.73	6
8	854.42	836.41	837.39	427.71	Q	611.40	593.39	594.37	306.20	5
9	953.49	935.48	936.47	477.22	V	483.34	465.33	466.31	242.17	4
10	1050.54	1032.53	1033.51	525.77	P	384.27	366.26	367.25	192.64	3
11	1206.64	1188.63	1189.61	603.82	R	287.22	269.21	270.19	144.11	2
12					L(-.98)	131.12	113.11	114.09	66.06	1

-10lgP: 70.20; Mass:1335.7445; Length: 12; ppm: 1.6; m/z: 668.8806; z: 2; RT: 50.11; Scan: 9920

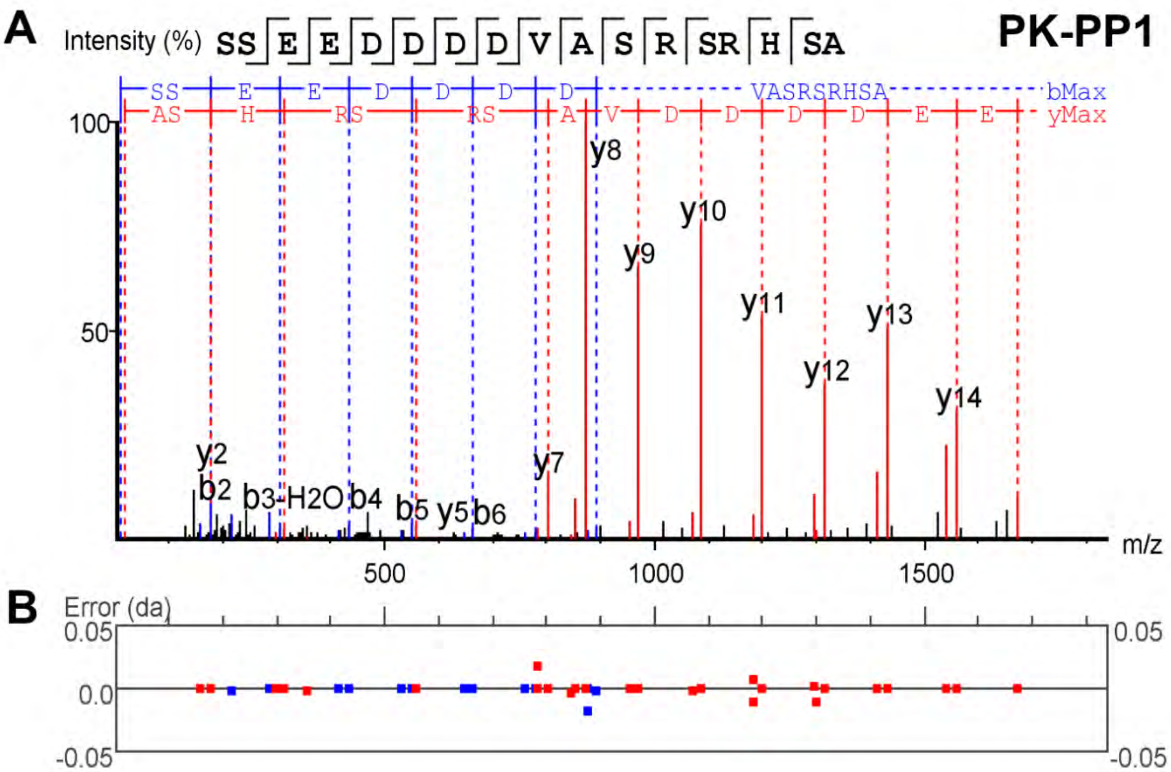

C

Ion Table

#	b	b-H2O	b-NH3	b (2+)	Seq	y	y-H2O	y-NH3	y (2+)	#
1	88.04	70.03	71.01	44.52	S					17
2	175.07	157.06	158.04	88.04	S	1775.74	1757.73	1758.72	888.37	16
3	304.11	286.10	287.09	152.56	E	1688.71	1670.70	1671.68	844.86	15
4	433.16	415.15	416.13	217.08	E	1559.67	1541.66	1542.64	780.33	14
5	548.18	530.17	531.16	274.59	D	1430.62	1412.61	1413.60	715.81	13
6	663.21	645.20	646.18	332.11	D	1315.60	1297.59	1298.58	658.30	12
7	778.24	760.23	761.21	389.62	D	1200.57	1182.55	1183.56	600.79	11
8	893.27	875.25	876.26	447.13	D	1085.54	1067.53	1068.52	543.27	10
9	992.33	974.32	975.31	496.67	V	970.52	952.51	953.49	485.76	9
10	1063.37	1045.36	1046.34	532.19	A	871.45	853.44	854.42	436.22	8
11	1150.40	1132.39	1133.38	575.70	S	800.41	782.38	783.39	400.71	7
12	1306.50	1288.49	1289.48	653.75	R	713.38	695.37	696.35	357.19	6
13	1393.54	1375.53	1376.51	697.27	S	557.28	539.27	540.25	279.14	5
14	1549.64	1531.63	1532.61	775.32	R	470.25	452.24	453.22	235.62	4
15	1686.70	1668.69	1669.67	843.85	H	314.15	296.14	297.12	157.57	3
16	1773.73	1755.72	1756.70	887.36	S	177.09	159.08	160.06	89.04	2
17					A	90.05	72.04	73.03	45.53	1

-10lgP: 72.74; Mass:1861.7673; Length: 17; ppm:-0.3 m/z: 466.4490; z: 4; RT: 23.63; Scan: 4287

4.15 RYa

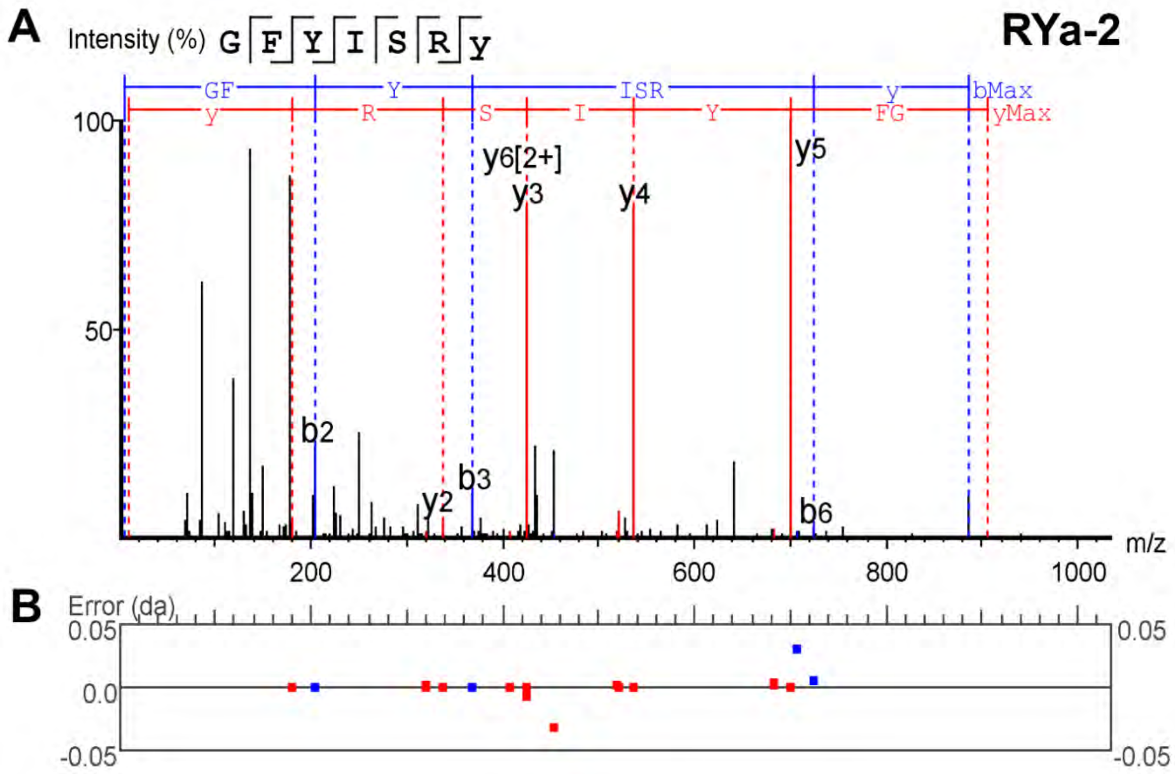

C

Ion Table

#	b	b-H2O	b-NH3	b (2+)	Seq	y	y-H2O	y-NH3	y (2+)	#
1	58.03	40.02	41.00	29.51	G					7
2	205.10	187.09	188.07	103.05	F	847.45	829.44	830.42	424.23	6
3	368.16	350.15	351.13	184.58	Y	700.38	682.36	683.35	350.69	5
4	481.25	463.23	464.22	241.12	I	537.31	519.30	520.29	269.16	4
5	568.28	550.27	551.25	284.64	S	424.23	406.22	407.20	212.62	3
6	724.37	706.37	707.32	362.69	R	337.20	319.19	320.17	169.10	2
7					Y(-.98)	181.10	163.09	164.07	91.05	1

-10lgP: 47.84; Mass:903.4603; Length: 7; ppm: 5.3 m/z: 452.7398; z: 2; RT: 46.14; Scan: 9086

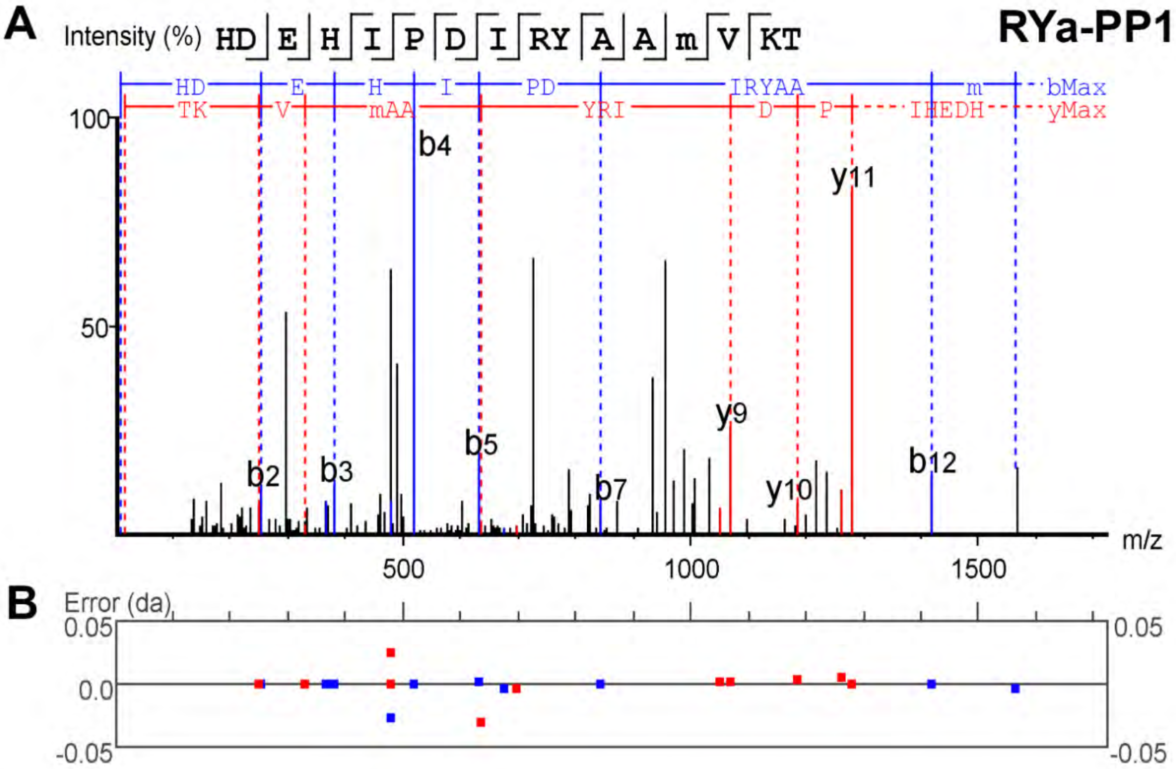

C

Ion Table

#	b	b-H2O	b-NH3	b (2+)	Seq	y	y-H2O	y-NH3	y (2+)	#
1	138.07	120.06	121.04	69.53	H					16
2	253.09	235.08	236.07	127.05	D	1774.88	1756.87	1757.85	887.94	15
3	382.14	364.13	365.11	191.57	E	1659.85	1641.84	1642.83	830.43	14
4	519.19	501.18	502.17	260.10	H	1530.81	1512.80	1513.78	765.90	13
5	632.28	614.27	615.25	316.64	I	1393.75	1375.74	1376.72	697.38	12
6	729.33	711.32	712.30	365.17	P	1280.67	1262.65	1263.64	640.83	11
7	844.36	826.35	827.33	422.68	D	1183.61	1165.60	1166.59	592.31	10
8	957.44	939.43	940.42	479.25	I	1068.58	1050.57	1051.56	534.79	9
9	1113.54	1095.53	1096.52	557.27	R	955.50	937.49	938.48	478.22	8
10	1276.61	1258.60	1259.58	638.80	Y	799.40	781.39	782.37	400.20	7
11	1347.64	1329.63	1330.62	674.33	A	636.37	618.33	619.31	318.67	6
12	1418.68	1400.67	1401.65	709.84	A	565.30	547.29	548.27	283.15	5
13	1565.72	1547.71	1548.69	783.36	M(+15.99)	494.26	476.25	477.24	247.63	4
14	1664.79	1646.77	1647.76	832.89	V	347.23	329.22	330.20	174.11	3
15	1792.88	1774.87	1775.85	896.94	K	248.16	230.15	231.13	124.58	2
16					T	120.07	102.05	103.04	60.53	1

-10lgP: 49.90; Mass:1910.9308; Length:16; ppm: 1.8 m/z: 478.7408; z: 4; RT: 43.85; Scan: 8604

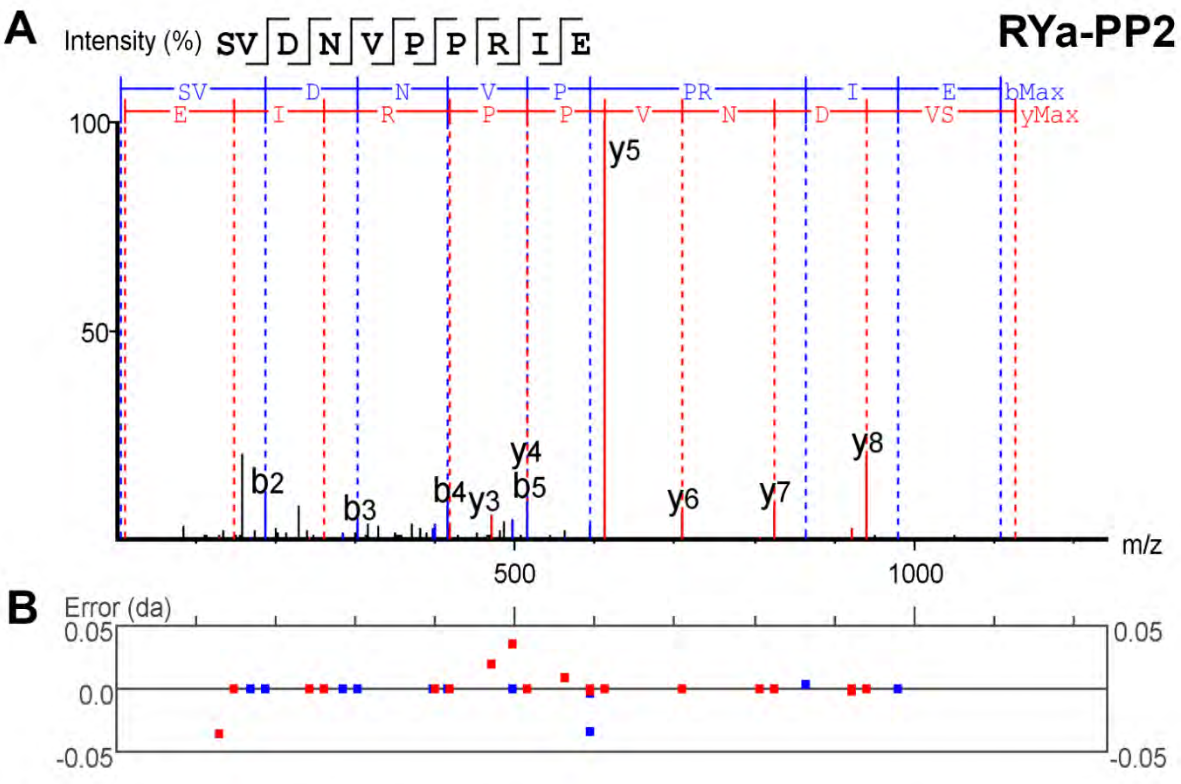

C Ion Table

#	b	b-H2O	b-NH3	b (2+)	Seq	y	y-H2O	y-NH3	y (2+)	#
1	88.04	70.03	71.01	44.52	S					10
2	187.11	169.10	170.08	94.05	V	1038.56	1020.55	1021.53	519.78	9
3	302.13	284.12	285.11	151.57	D	939.49	921.48	922.46	470.22	8
4	416.18	398.17	399.15	208.59	N	824.46	806.45	807.43	412.73	7
5	515.25	497.24	498.22	258.12	V	710.42	692.41	693.39	355.71	6
6	612.30	594.32	595.28	306.65	P	611.35	593.34	594.32	306.18	5
7	709.35	691.34	692.33	355.18	P	514.30	496.29	497.24	257.65	4
8	865.45	847.44	848.43	433.23	R	417.25	399.24	400.22	209.12	3
9	978.54	960.53	961.51	489.77	I	261.15	243.13	244.12	131.07	2
10					E	148.06	130.09	131.03	74.53	1

-10lgP: 64.77; Mass:1124.5825; Length:10; ppm: -0.3 m/z: 563.2983; z: 2; RT: 41.13; Scan: 8057

4.16 TKRP

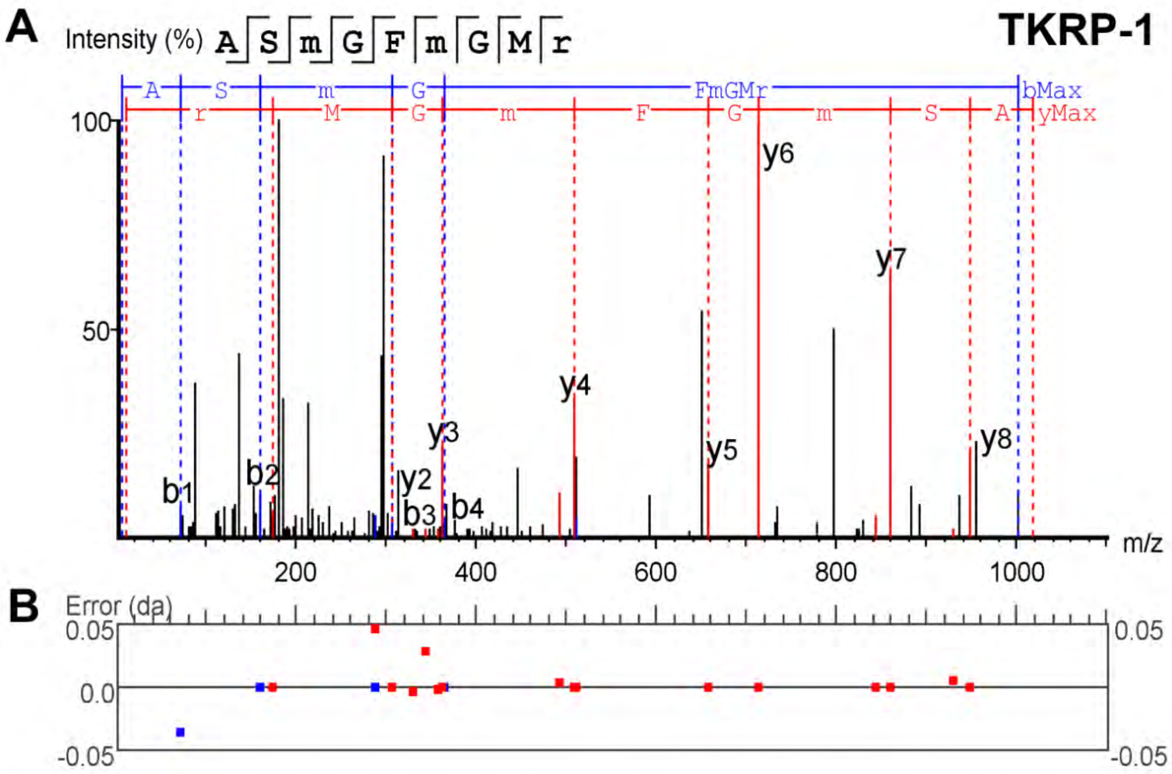

C

Ion Table

#	b	b-H2O	b-NH3	b (2+)	Seq	y	y-H2O	y-NH3	y (2+)	#
1	72.08	54.03	55.02	36.52	A					9
2	159.08	141.07	142.05	80.04	S	947.39	929.37	930.36	474.19	8
3	306.11	288.10	289.09	153.56	M(+15.99)	860.36	842.35	843.33	430.68	7
4	363.13	345.12	346.11	182.07	G	713.32	695.31	696.30	357.16	6
5	510.20	492.19	493.18	255.60	F	656.30	638.29	639.27	328.65	5
6	657.24	639.23	640.21	329.12	M(+15.99)	509.23	491.22	492.21	255.12	4
7	714.26	696.25	697.23	357.63	G	362.20	344.16	345.17	181.60	3
8	845.30	827.29	828.27	423.15	M	305.18	287.16	288.10	153.09	2
9					R(-.98)	174.13	156.12	157.11	87.57	1

-10lgP: 44.90; Mass:1017.4194; Length: 9; ppm:1.4 m/z: 509.7177; z: 2; RT: 33.21; Scan: 6285

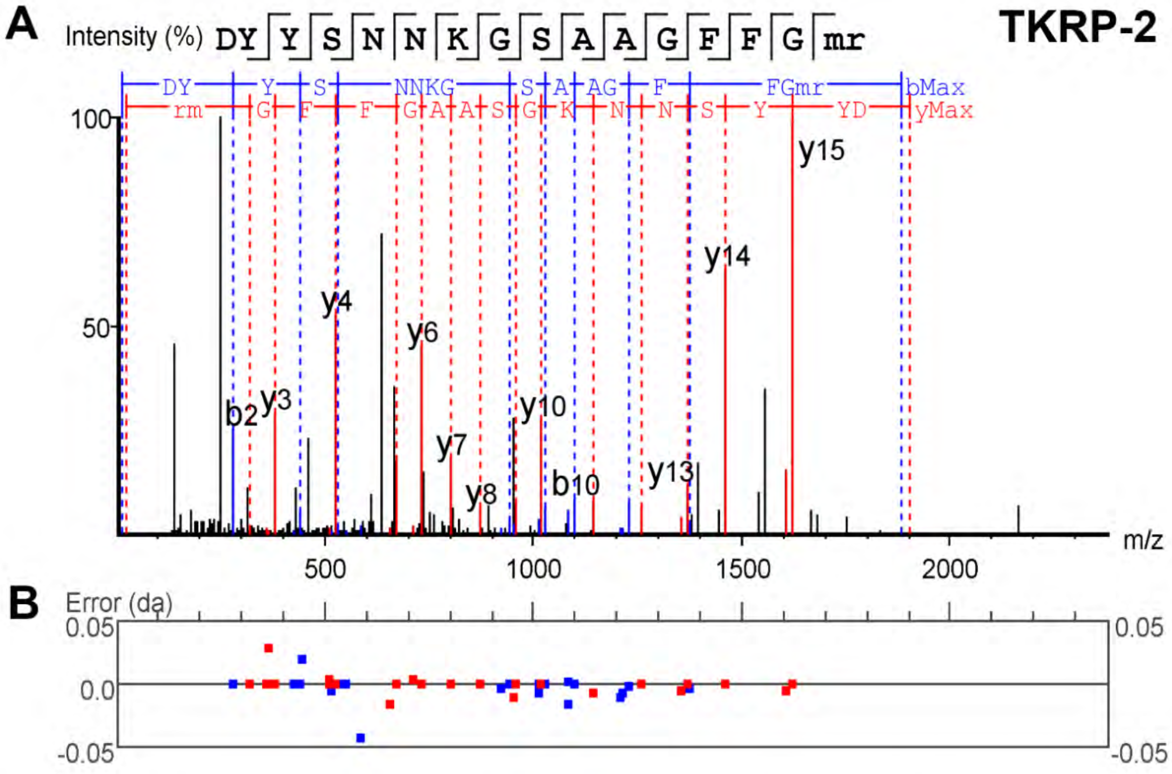

C

Ion Table

#	b	b-H2O	b-NH3	b (2+)	Seq	y	y-H2O	y-NH3	y (2+)	#
1	116.03	98.02	99.01	58.52	D					17
2	279.10	261.09	262.07	140.05	Y	1784.82	1766.81	1767.79	892.91	16
3	442.16	424.15	425.13	221.58	Y	1621.75	1603.74	1604.73	811.38	15
4	529.19	511.18	512.17	265.10	S	1458.69	1440.68	1441.66	729.85	14
5	643.24	625.23	626.21	322.12	N	1371.66	1353.65	1354.64	686.33	13
6	757.28	739.27	740.25	379.14	N	1257.62	1239.61	1240.59	629.31	12
7	885.37	867.36	868.35	443.17	K	1143.58	1125.56	1126.55	572.29	11
8	942.39	924.39	925.37	471.70	G	1015.48	997.47	998.45	508.23	10
9	1029.43	1011.42	1012.41	515.22	S	958.45	940.45	941.43	479.73	9
10	1100.47	1082.45	1083.45	550.73	A	871.42	853.41	854.40	436.21	8
11	1171.50	1153.49	1154.47	586.29	A	800.39	782.38	783.36	400.69	7
12	1228.53	1210.52	1211.50	614.76	G	729.35	711.34	712.32	365.15	6
13	1375.60	1357.58	1358.56	688.30	F	672.33	654.34	655.30	336.66	5
14	1522.66	1504.65	1505.63	761.83	F	525.26	507.25	508.23	263.13	4
15	1579.68	1561.67	1562.65	790.34	G	378.19	360.18	361.17	189.60	3
16	1726.72	1708.71	1709.69	863.86	M(+15.99)	321.17	303.16	304.14	161.09	2
17					R(-.98)	174.13	156.12	157.11	87.57	1

-10lgP: 74.72; Mass:1898.8369; Length: 17; ppm: -2.3 m/z: 633.9515; z: 3; RT: 45.80; Scan: 8925

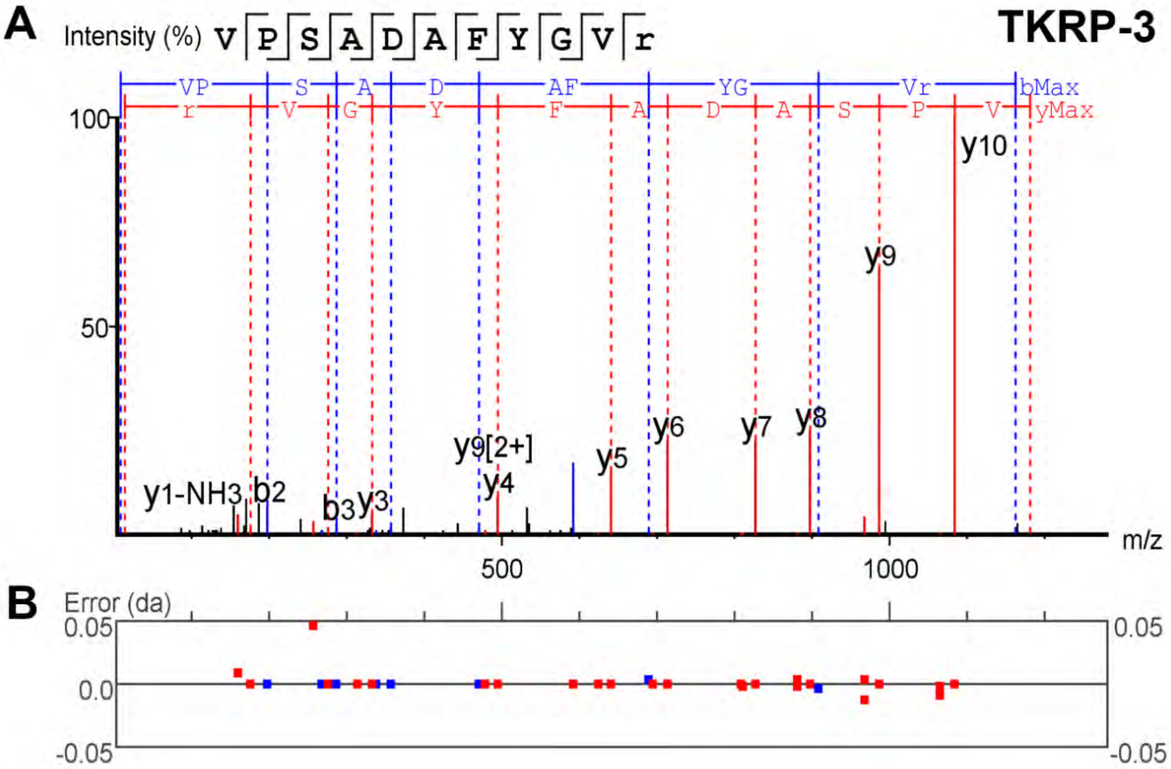

C

Ion Table

#	b	b-H2O	b-NH3	b (2+)	Seq	y	y-H2O	y-NH3	y (2+)	#
1	100.08	82.07	83.05	50.54	V					11
2	197.13	179.12	180.10	99.06	P	1081.54	1063.53	1064.52	541.27	10
3	284.16	266.15	267.13	142.58	S	984.49	966.47	967.48	492.75	9
4	355.20	337.19	338.17	178.10	A	897.46	879.44	880.43	449.23	8
5	470.22	452.21	453.20	235.61	D	826.42	808.41	809.40	413.71	7
6	541.26	523.25	524.24	271.13	A	711.39	693.38	694.37	356.20	6
7	688.33	670.32	671.30	344.67	F	640.36	622.35	623.33	320.68	5
8	851.39	833.38	834.37	426.20	Y	493.29	475.28	476.26	247.14	4
9	908.42	890.40	891.39	454.71	G	330.22	312.21	313.20	165.61	3
10	1007.48	989.47	990.46	504.24	V	273.20	255.19	256.13	137.10	2
11					R(-.98)	174.14	156.12	157.10	87.57	1

-10lgP: 70.92; Mass:1180.5876; Length: 11; ppm: 0.9 m/z: 591.3016; z: 2; RT: 48.21; Scan: 9436

TKRP-PP1

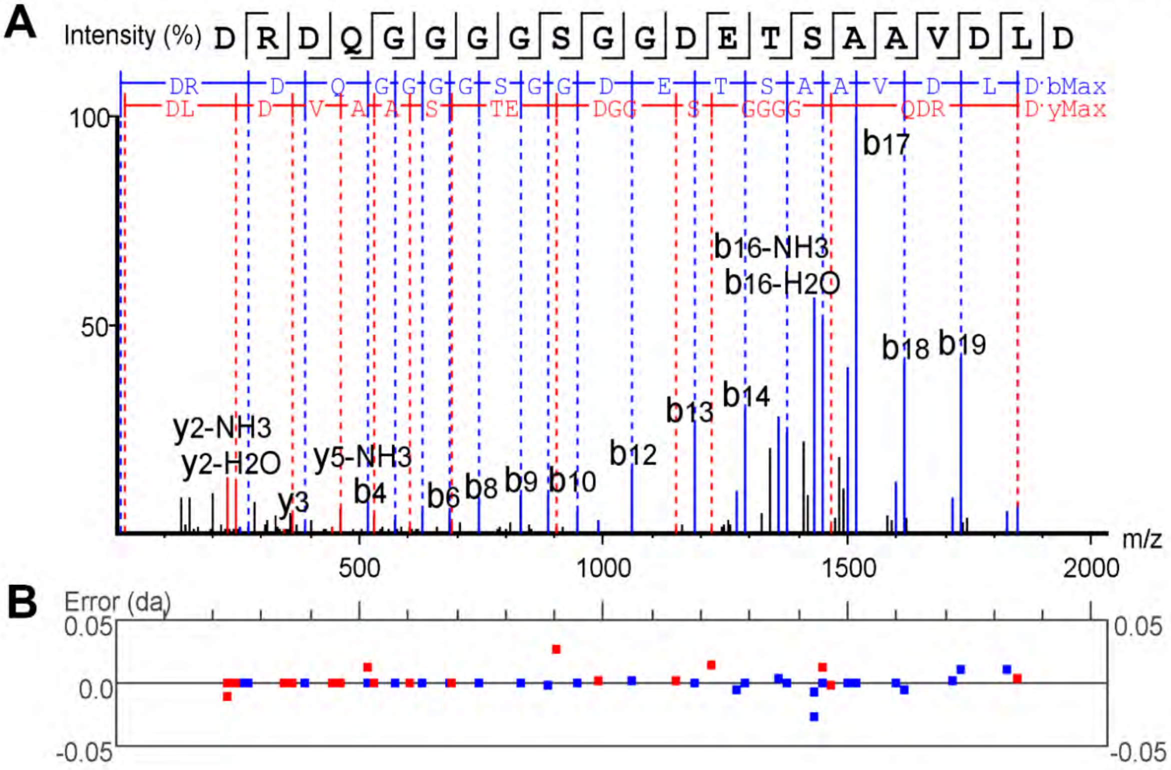

C

Ion Table

#	b	b-H2O	b-NH3	b (2+)	Seq	y	y-H2O	y-NH3	y (2+)	#
1	116.03	98.02	99.01	58.52	D					21
2	272.14	254.13	255.11	136.57	R	1863.80	1845.78	1846.77	932.40	20
3	387.16	369.15	370.14	194.08	D	1707.69	1689.68	1690.67	854.35	19
4	515.22	497.21	498.19	258.11	Q	1592.67	1574.66	1575.64	796.83	18
5	572.24	554.23	555.22	286.62	G	1464.61	1446.60	1447.57	732.80	17
6	629.26	611.25	612.24	315.13	G	1407.59	1389.58	1390.56	704.29	16
7	686.29	668.28	669.26	343.64	G	1350.57	1332.56	1333.54	675.78	15
8	743.31	725.30	726.28	372.15	G	1293.54	1275.53	1276.52	647.27	14
9	830.34	812.33	813.31	415.67	S	1236.52	1218.51	1219.48	618.76	13
10	887.36	869.35	870.33	444.18	G	1149.49	1131.48	1132.46	575.25	12
11	944.38	926.37	927.36	472.69	G	1092.47	1074.46	1075.44	546.73	11
12	1059.41	1041.40	1042.38	530.20	D	1035.45	1017.44	1018.42	518.22	10
13	1188.45	1170.44	1171.42	594.73	E	920.42	902.41	903.37	460.71	9
14	1289.50	1271.49	1272.47	645.25	T	791.38	773.37	774.35	396.19	8
15	1376.53	1358.52	1359.50	688.77	S	690.33	672.32	673.30	345.67	7
16	1447.57	1429.57	1430.57	724.28	A	603.30	585.29	586.27	302.15	6
17	1518.61	1500.60	1501.58	759.80	A	532.26	514.25	515.22	266.63	5
18	1617.68	1599.66	1600.65	809.34	V	461.22	443.21	444.20	231.11	4
19	1732.69	1714.69	1715.67	866.85	D	362.16	344.15	345.13	181.58	3
20	1845.78	1827.76	1828.76	923.39	L	247.13	229.12	230.11	124.06	2
21					D	134.04	116.03	117.02	67.52	1

-10lgP: 83.63; Mass:1977.8147; Length: 21; ppm: 0.4 m/z: 989.9150; z: 2; RT: 41.14; Scan: 7816

TKRP-PP2

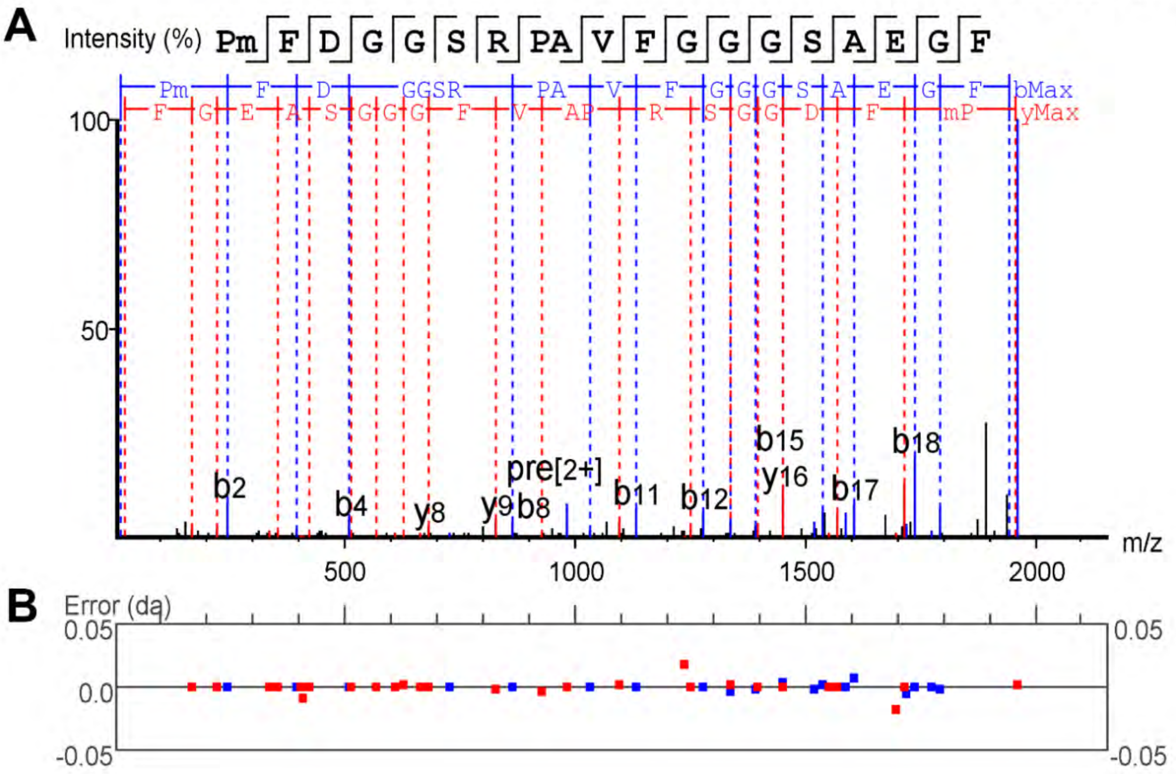

C

Ion Table

#	b	b-H2O	b-NH3	b (2+)	Seq	y	y-H2O	y-NH3	y (2+)	#
1	98.06	80.05	81.03	49.53	P					20
2	245.10	227.09	228.07	123.05	M(+15.99)	1861.82	1843.81	1844.79	931.41	19
3	392.16	374.15	375.14	196.58	F	1714.78	1696.77	1697.77	857.89	18
4	507.19	489.18	490.16	254.10	D	1567.71	1549.70	1550.69	784.36	17
5	564.21	546.20	547.19	282.61	G	1452.69	1434.68	1435.66	726.84	16
6	621.23	603.22	604.21	311.12	G	1395.66	1377.65	1378.64	698.33	15
7	708.27	690.26	691.24	354.63	S	1338.64	1320.63	1321.62	669.82	14
8	864.37	846.36	847.34	432.68	R	1251.61	1233.60	1234.57	626.31	13
9	961.42	943.41	944.39	481.21	P	1095.51	1077.50	1078.48	548.26	12
10	1032.46	1014.45	1015.43	516.73	A	998.46	980.45	981.43	499.73	11
11	1131.53	1113.52	1114.50	566.26	V	927.43	909.41	910.39	464.21	10
12	1278.59	1260.58	1261.57	639.80	F	828.35	810.34	811.33	414.68	9
13	1335.62	1317.60	1318.59	668.31	G	681.28	663.27	664.26	341.14	8
14	1392.64	1374.63	1375.61	696.82	G	624.26	606.25	607.24	312.63	7
15	1449.65	1431.65	1432.63	725.33	G	567.24	549.23	550.21	284.12	6
16	1536.69	1518.68	1519.66	768.85	S	510.22	492.21	493.19	255.61	5
17	1607.72	1589.72	1590.70	804.36	A	423.19	405.18	406.17	212.09	4
18	1736.77	1718.77	1719.74	868.89	E	352.15	334.14	335.12	176.58	3
19	1793.79	1775.78	1776.76	897.40	G	223.11	205.10	206.08	112.05	2
20					F	166.09	148.08	149.06	83.54	1

-10lgP: 82.06; Mass:1957.8628; Length: 20; ppm: 3.2; m/z: 979.9418; z: 2; RT: 56.39; Scan: 11161

5 Supporting Information S5:

BUSCO summary table for all analysed transcriptomes.

Sample	Complete (C)	Single-copy (S)	Duplicated (D)	Fragmented (F)	Missing (M)	Total BUSCOs (n)
SRR063706	41.6%	41.2%	0.4%	28.6%	29.8%	255
SRR7037537	84.7%	49.8%	34.9%	4.3%	11.0%	255
SRR7037541	80.4%	45.9%	34.5%	7.5%	12.2%	255
SRR9209839	96.9%	52.5%	44.3%	2.0%	1.2%	255
SRR9209842	97.6%	52.5%	45.1%	0.8%	1.6%	255
SRR12776646	77.6%	44.3%	33.3%	5.5%	16.9%	255
